# Global, regional, and national burden and trends of pancreatic cancer, 1990–2021: a systematic analysis for the Global Burden of Disease Study 2021

**DOI:** 10.3389/fonc.2025.1671856

**Published:** 2025-11-03

**Authors:** Chunlong Liu, Pengpeng Liu, Xue Liu, Wang Niu, Panpan Wu, Jiangtao Yu

**Affiliations:** ^1^ Department of Hepatobiliary and Pancreatic Surgery, Fuyang People’s Hospital, Anhui Medical University, Fuyang, China; ^2^ Department of Hepatobiliary and Pancreatic Surgery, Fuyang People’s Hospital, Bengbu Medical University, Fuyang, China

**Keywords:** pancreatic cancer, burden trends, Global Burden of Disease, risk factor, cross-national inequalities

## Abstract

**Background:**

To evaluate temporal trends, associated risk factors, and cross-national inequalities in the distribution of pancreatic cancer from 1990 to 2021.

**Methods:**

Temporal trends were measured using the average annual percent change and annual percent change. A comparative risk assessment framework was employed to estimate the proportion of disability-adjusted life years (DALYs) attributable to specific risk factors for pancreatic cancer. In addition, the unequal distribution of the global burden of pancreatic cancer was quantified using the inequality slope index and the concentration index, as recommended by the World Health Organization.

**Results:**

From 1990 to 2021, the global incidence, prevalence, mortality and DALYs associated with pancreatic cancer have increased in absolute numbers. At the regional level, Central Asia recorded the highest values for all four indicators, while Western Sub-Saharan Africa experienced the most pronounced escalation in disease burden. Notably, the burden of pancreatic cancer was consistently higher in males than in females throughout the study period. Cross-national inequalities analysis reveals that disparities in pancreatic cancer burden are concentrated in countries with relatively higher socio-demographic index. To date, the high fasting plasma glucose remained the major risk factor that influenced the DALYs of pancreatic cancer, followed by smoking and high body mass index.

**Conclusion:**

The global burden of pancreatic cancer is rising, particularly among males and in high-income regions. This trend highlights the urgent need for targeted prevention strategies and improved management of modifiable risk factors, with a specific focus on these high-risk populations.

## 1 Introduction

According to GLOBOCAN 2020, pancreatic cancer ranks as the 12th most common cancer globally and the seventh leading cause of cancer-related death (1). Pancreatic cancer has historically been recognized as a lethal malignancy characterized by poor prognosis, with a 5-year survival rate of only 11.5% (2). Additionally, there are significant regional differences in the incidence of pancreatic cancer, with approximately 5 times higher in countries with higher human development indexes compared to developing countries (3). Global data indicate a sustained increase in pancreatic cancer burden, which is projected to become the second leading cause of cancer death in Western countries (4). This increase partly reflects population aging and improved diagnostic capacity, particularly in high-income countries (5). In addition, regional disparities may result from variations in the prevalence of major risk factors such as smoking, diabetes, and alcohol consumption (6, 7). While a recent study by Liu et al. (8) explored risk factors and inequalities in pancreatic cancer burden based on DALYs, our study extends this work by simultaneously examining incidence, prevalence, mortality, and DALYs. This multidimensional assessment allows for a more comprehensive understanding of the epidemiological patterns and disparities in pancreatic cancer worldwide. Moreover, given the substantial challenges in achieving the targets set by the World Health Organization (WHO) and the United Nations Sustainable Development Goals, particularly the objective of reducing premature cancer mortality by one-third by 2030 (9). Therefore, an updated and comprehensive assessment of the global epidemiology of pancreatic cancer is urgently needed to inform targeted prevention and control strategies.

The Global Burden of Diseases (GBD), Injuries, and Risk Factors Study provides valuable data for quantifying disease epidemiology and burden (2). Data from the GBD also can be used to monitor health inequalities, which describe differences and changes in health indicators between countries based on national averages (10). In this study, we aimed to comprehensively assess the burden and spatiotemporal trends of pancreatic cancer globally, regionally, and nationally by geographic location, age, sex, and socio-demographic index (SDI). We further quantified the impact of major risk factors for pancreatic cancer, and evaluated cross-national inequalities along with the association between SDI and pancreatic cancer burden. These findings may help identify regions and populations most significantly affected by pancreatic cancer, thereby informing resource allocation and guiding medical policy development for healthcare providers.

## 2 Methods

### 2.1 Data source

GBD 2021 presented, for the first time, estimates of global health loss due to the COVID-19 pandemic, which estimated epidemiological quantity of interest for 288 causes of death by age-sex-location-year for 25 age groups (ranging birth to 95 years and older), sex (males, females, and both sexes combined), location (204 countries and territories grouped into 21 regions and seven super-regions), and every year from 1990 to 2021 (2, 11). Data on the incidence, prevalence, mortality, and disability-adjusted life years (DALYs) related to pancreatic cancer, including their 95% uncertainty intervals (UIs), were obtained from the GBD 2021 study. DALYs are the sum of years of life lost (YLLs) and years lived with disability (YLDs). YLDs were calculated by multiplying cause-age-sex-location-year-specific prevalence of sequelae by their respective disability weights, for each disease and injury. YLLs were calculated by multiplying cause-age-sex-location-year-specific deaths by the standard life expectancy at the age that death occurred. The Bayesian meta-regression modelling tool, DisMod-MR 2.1, was used to ensure internally consistency of all available data on incidence, prevalence, remission, and mortality for a disease (2). Detailed methods for estimating YLLs, YLDs and DALYs have been described in previous GBD studies (12). In this study, cancers were categorized according to the International Classification of Diseases, 9th and 10th revisions. Pancreatic cancer includes all diagnoses coded C25.0 to C25.9 (malignant neoplasm of the pancreas) (**Supplementary Table S1**).

In GBD 2021, “risk factors” are defined as attributes that are causally associated with an increased incidence or prevalence of diseases. The GBD Risk Factor Collaborators assessed 88 behavioral, environmental or occupational, and metabolic risks (11). We focused on three major risk factors for pancreatic cancer identified in the GBD 2021 study: smoking, high body mass index (BMI), and high fasting plasma glucose. Our analysis included data on pancreatic cancer-related DALYs attributable to these factors, with additional stratification by region to reveal geographical variations in their impact. This comprehensive approach not only quantifies the direct burden of pancreatic cancer but also underscores the contribution of modifiable risk factors, offering valuable insights into potential strategies for disease prevention and intervention.

The SDI is an index used to assess social development, which comprehensively measures a nation’s socioeconomic status based on per capita income, educational attainment and total fertility rate. SDI values range from 0 to 1, with higher values indicating higher levels of socioeconomic development. The geometric mean of these three indices yields the national-level SDI value. Based on SDI values, 204 countries and territories are categorized into five groups: low SDI (0-0.4658), low-middle SDI (0.4658-0.6188), middle SDI (0.6188-0.7120), high-middle SDI (0.7120-0.8103), and high SDI (0.8103-1) (13).

### 2.2 Age-standardized rates and temporal joinpoint analysis

In this research, age-standardized rates (ASRs) were employed to quantify the incidence, prevalence, mortality, and DALYs associated with pancreatic cancer. The ASR (per 100,000 population) was calculated using the following formula:


$$\text{ASR}=\frac{\sum_{i=1}^{A}a_{i}w_{i}}{\sum_{i=1}^{A}w_{i}}\times 100,000$$

where $a_{i}$ refers to the i^th^ age group and the number (or weight) ( $w_{i}$ ) of people in the same age subgroup i in the selected reference standard population. ASR was determined by adjusting the global age distribution to a standard population (14), which is crucial for comparing populations with varying age structures or analyzing changes in the age structure of a single group over time. Additionally, joinpoint regression analysis was employed to assess the temporal trends in ASR for pancreatic cancer, identifying significant inflection points within the time series. The short-term trends were expressed as an annual percentage change (APC) and long-term trends as an average annual percentage change (AAPC). Trends were classified as increasing or decreasing if the AAPC or APC was statistically significant (two-sided *P*-value < 0.05) and as stable otherwise. Temporal trends were analyzed using the Joinpoint Regression Program (version 5.4.0; https://surveillance.cancer.gov/joinpoint/), allowing a maximum of five joinpoints.

### 2.3 Cross-national inequality

The slope of inequality index and concentration index are two standard indicators of absolute and relative gradient inequality, respectively, used to quantify the inequality in the distribution of pancreatic cancer across countries (15). The slope index of inequality was calculated by regressing each country’s crude DALYs and crude mortality rates per 100,000 population (across all age groups) on its social-development rank. The concentration index is computed by numerically integrating the area under the Lorenz concentration curve (16). We estimated 95% UIs using a Monte Carlo simulation. For each country and year, 1,000 samples were drawn from the posterior distribution to recalculate inequality indicators, with the 2.5th and 97.5th percentiles representing the UIs. The larger the absolute values of concentration index and slope index, the higher the level of inequality (17). Studies have concluded that absolute values of concentration index ranging from 0.2 to 0.3 represent a relatively high level of inequality (18).

### 2.4 Statistical analysis

All statistical analyses were conducted using R software, version 4.3.1, and a two-sided P-value of less than 0.05 was deemed statistically significant.

## 3 Results

### 3.1 Global level

**Figure 1:**
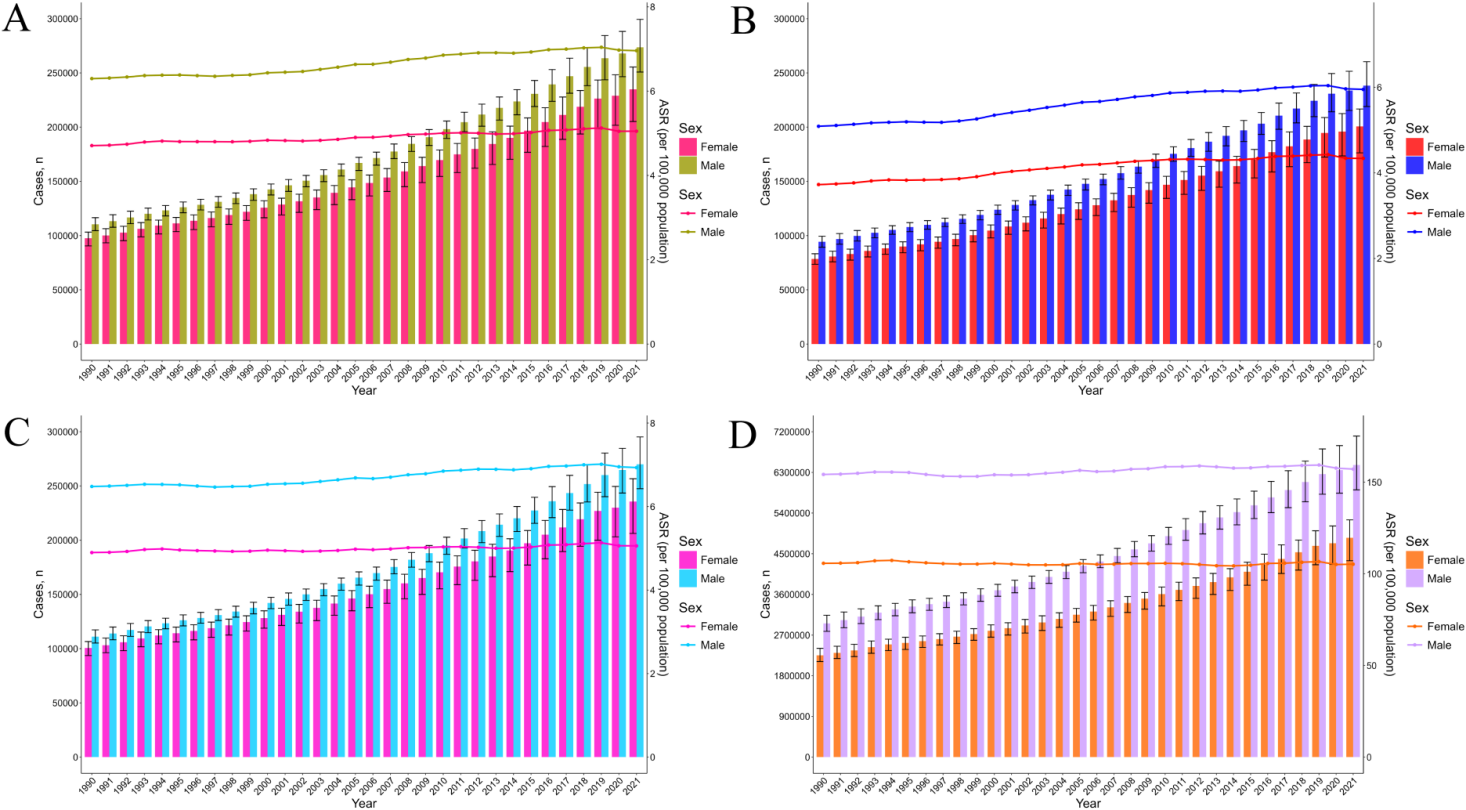
The trends in number and ASR of incidence **(A)**, prevalence **(B)**, mortality **(C)**, and DALYs **(D)** for pancreatic cancer among female and male, from 1990 to 2021. DALYs, disability-adjusted life years.

From 1990 to 2021, the global absolute incidence of pancreatic cancer increased by 144.6%, from 207,905.2 cases (95% uncertainty interval [UI]: 196,649.4–217,778.5) in 1990 to 508,532.7 cases (95% UI: 462,090.89–547,207.6) in 2021 (**Figure 1A**). Similarly, the age-standardized incidence rate (ASIR) increased by 9%, rising from 5.47 (95% UI: 5.16-5.73) to 5.96 (95% UI: 5.39-6.42) per 100,000 population (**Table 1**). Absolute prevalence rose by 154%, and the age-standardized prevalence rate (ASPR) increased by 16.6% (**Figure 1B**; **Supplementary Table S2**). Additionally, absolute mortality increased by 139%, accompanied by a 5.1% rise in the age-standardized mortality rate (ASMR) (**Figure 1C**; **Supplementary Table S3**). The global absolute number of DALYs, reflecting the disease burden, increased by 117.2%, whereas the age-standardized DALYs rates (ASDR) remained stable at approximately 130 per 100,000 population (**Figure 1D**; **Supplementary Table S4**).

**Table 1:** Incidence case numbers and ASRs of pancreatic cancer in 1990 and 2021, by sex, across 5 SDI regions and 21 GBD regions, with AAPC estimates from 1990 to 2021.

Location	Incidence				
	1990		2021		1990-2021
	Number (95% UI)	ASR (95% UI)	Number (95% UI)	ASR (95% UI)	AAPC (95% CI)
Global	207905.2 (196649.4-217778.5)	5.47 (5.16-5.73)	508532.7 (462090.9-547207.6)	5.96 (5.39-6.42)	0.27^*^ (0.19 to 0.36)
SDI regions					
High SDI	97522.0 (92265.8-100694.3)	8.75 (8.29-9.03)	217146.3 (193740.7-231692.7)	10.00 (9.08-10.61)	0.42^*^ (0.34 to 0.51)
High-middle SDI	66491.4 (62906.2-70237.6)	6.75 (6.38-7.14)	148297.1 (132453.8-164406.4)	7.47 (6.67-8.27)	0.36^*^ (0.26 to 0.45)
Middle SDI	32383.1 (29494.7-35579.9)	3.18 (2.92-3.48)	103575.8 (91436.1-116662.5)	3.88 (3.43-4.36)	0.65^*^ (0.53 to 0.76)
Low-middle SDI	8316.2 (7055.1-9705.6)	1.39 (1.18-1.62)	31147.9 (28918.9-33650.6)	2.20 (2.04-2.37)	1.5^*^ (1.38 to 1.62)
Low SDI	2902.2 (2251.0-3479.2)	1.31 (1.02-1.57)	7830.3 (6493.5-9394.4)	1.59 (1.33-1.90)	0.65^*^ (0.56 to 0.74)
GBD regions					
Andean Latin America	868.0 (729.1-1021.8)	4.38 (3.68-5.16)	3009.9 (2323.4-3797.0)	5.17 (3.99-6.52)	0.57^*^ (0.09 to 1.04)
Australasia	1815.4 (1721.9-1895.2)	7.70 (7.28-8.03)	4774.1 (4244.6-5131.2)	8.62 (7.77-9.23)	0.34 (-0.12 to 0.81)
Caribbean	1191.1 (1118.9-1265.9)	4.66 (4.37-4.95)	2747.3 (2426.6-3098.9)	5.09 (4.50-5.75)	0.28^*^ (0.02 to 0.54)
Central Asia	1416.7 (1249.6-1646.8)	3.00 (2.63-3.51)	3468.0 (3054.6-3893.0)	4.27 (3.77-4.77)	1.2^*^ (0.61 to 1.8)
Central Europe	12157.8 (11640.0-12603.1)	8.13 (7.78-8.44)	20702.8 (18929.7-22371.4)	9.27 (8.49-10.04)	0.43^*^ (0.25 to 0.62)
Central Latin America	3506.3 (3405.7-3595.2)	4.36 (4.21-4.48)	11283.7 (10098.0-12537.3)	4.54 (4.06-5.04)	0.11 (-0.11 to 0.34)
Central Sub-Saharan Africa	476.4 (381.5-579.1)	2.19 (1.78-2.64)	1208.5 (840.2-1666.0)	2.27 (1.57-3.17)	0.12^*^ (0 to 0.24)
East Asia	39030.0 (32969.7-45464.1)	4.51 (3.83-5.24)	122830.8 (98826.2-148855.0)	5.64 (4.56-6.80)	0.74^*^ (0.55 to 0.93)
Eastern Europe	20040.9 (19040.7-21303.8)	7.12 (6.76-7.57)	29093.9 (26856.1-31591.2)	8.28 (7.65-8.98)	0.58^*^ (0.3 to 0.86)
Eastern Sub-Saharan Africa	1195.0 (927.3-1460.5)	1.65 (1.28-2.01)	3268.9 (2665.9-4173.8)	2.01 (1.66-2.53)	0.64^*^ (0.56 to 0.72)
High-income Asia Pacific	18561.8 (17616.4-19277.7)	9.38 (8.84-9.76)	54773.4 (46040.3-59823.7)	10.69 (9.30-11.53)	0.44^*^ (0.35 to 0.53)
High-income North America	33069.7 (30983.6-34192.9)	9.31 (8.77-9.62)	68168.2 (62281.0-71457.3)	10.20 (9.38-10.65)	0.26^*^ (0.17 to 0.35)
North Africa and Middle East	4704.9 (3853.2-5554.0)	2.89 (2.35-3.40)	19612.4 (17208.6-22096.5)	4.52 (3.97-5.08)	1.47^*^ (1.3 to 1.65)
Oceania	50.9 (40.1-65.2)	1.85 (1.49-2.34)	162.6 (131.9-207.4)	2.28 (1.86-2.89)	0.68 (0.56 to 0.79)
South Asia	5708.0 (4495.0-6903.4)	1.00 (0.78-1.21)	20577.4 (18297.4-22728.5)	1.41 (1.25-1.56)	1.13^*^ (0.96 to 1.3)
Southeast Asia	5583.9 (4807.6-6413.2)	2.20 (1.90-2.52)	21701.9 (18693.9-25153.8)	3.33 (2.88-3.87)	1.37^*^ (1.3 to 1.44)
Southern Latin America	4075.5 (3834.6-4335.3)	8.87 (8.34-9.44)	7590.8 (7019.0-8087.8)	8.61 (7.98-9.17)	-0.09 (-0.3 to 0.12)
Southern Sub-Saharan Africa	1002.6 (861.4-1235.3)	3.80 (3.25-4.74)	3231.2 (2842.6-3577.2)	5.76 (5.09-6.36)	1.37^*^ (1.04 to 1.7)
Tropical Latin America	4184.4 (3992.4-4325.1)	4.75 (4.49-4.92)	15016.0 (13928.1-15736.5)	5.86 (5.42-6.16)	0.72^*^ (0.42 to 1.02)
Western Europe	48414.4 (45663.4-50531.5)	8.27 (7.83-8.62)	91720.5 (82405.3-98443.8)	9.54 (8.71-10.16)	0.47^*^ (0.34 to 0.6)
Western Sub-Saharan Africa	851.4 (726.9-980.9)	1.00 (0.86-1.15)	3590.3 (2986.0-4167.5)	1.92 (1.62-2.22)	2.13^*^ (2.05 to 2.21)

ASR, age-standardized rate; SDI, sociodemographic index; GBD, Global Burden of Disease; AAPC, average annual percentage change; UI, uncertainty interval; CI, confidence interval. * Statistically significant, P < .05.

A detailed analysis using APC and AAPC was conducted to examine temporal trends globally. The ASIR exhibited the most substantial increase during 2002–2010 (APC = 0.62, 95% confidence interval [CI]: 0.52 to 0.72), followed by a significant decrease during 2019–2021 (APC = −0.80, 95% CI: −1.52 to −0.07). Similarly, the ASPR showed a parallel trend, with a notable decrease (APC = −0.94, 95% CI: −1.84 to −0.02) during 2019–2021. The ASMR demonstrated a significant decline solely during the period 2019–2021 (APC = −0.79, 95% CI: −1.54 to −0.04). The ASDR exhibited minor fluctuations, but the overall AAPC was close to zero, indicating relative stability over the studied period (**Supplementary Figure S1**).

### 3.2 Regional and national levels

Significant differences in the burden of pancreatic cancer were observed across the 21 GBD regions. In 2021, the highest ASIR and ASPR occurred in the High-income Asia Pacific region, at 10.69 (95% UI: 9.30–11.53) and 11.20 (95% UI: 9.69–12.17) per 100,000 population, respectively. Conversely, the lowest rates were found in South Asia, with an ASIR of 1.41 (95% UI: 1.25–1.56) and an ASPR of 1.08 (95% UI: 0.96–1.19) per 100,000 population. Notably, no significant declining trends in ASIR or ASPR were detected across the 21 regions. Among these, Western Sub-Saharan Africa experienced the most pronounced increases in ASIR and ASPR, with average AAPCs of 2.13 (95% CI: 2.05–2.21) and 2.13 (95% CI: 2.08–2.19), respectively. Similarly, the ASMR and ASDR across the 21 GBD regions exhibited upward or stable trends over the past three decades. Western Sub-Saharan Africa again recorded the largest increases in ASMR and ASDR, with AAPCs of 2.13 (95% CI: 2.05–2.21) and 2.05 (95% CI: 1.95–2.14), respectively (**Supplementary Figures S2**, **S3**).

**Figure 2:**
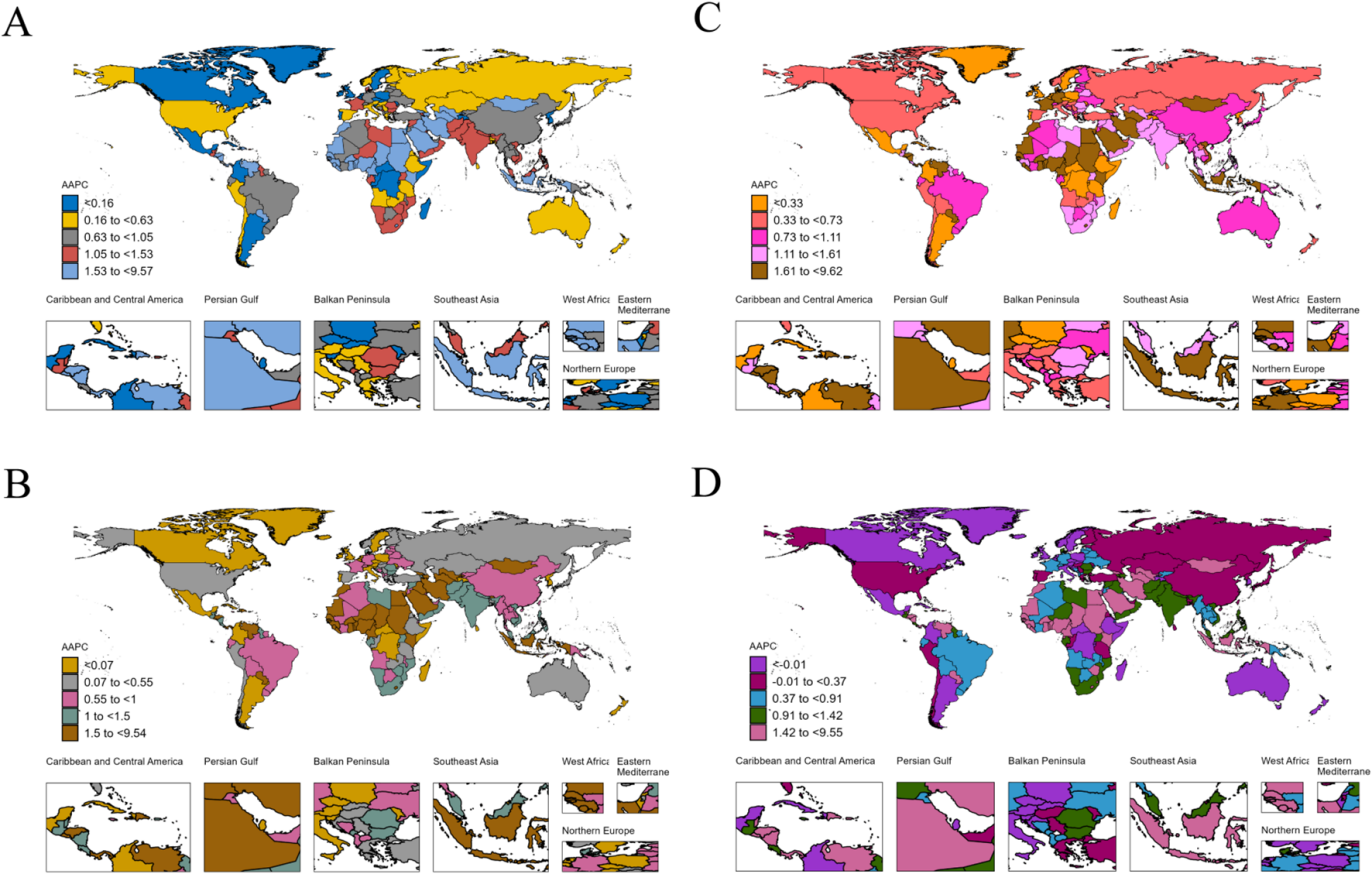
The AAPC of incidence **(A)**, prevalence **(B)**, mortality **(C)**, and DALYs **(D)** for pancreatic cancer worldwide from 1990 to 2021. DALYs, disability-adjusted life years; AAPC, average annual percentage change.

The absolute numbers, ASRs, and their temporal trends demonstrated substantial regional disparities across 204 countries and territories (**Figure 2**, **Supplementary Figures S4**, **S5**). China, the United States, and Japan reported the highest case counts in terms of incidence, prevalence, mortality, and DALYs. Regarding incidence, the highest ASIRs in 2021 were observed in Greenland, Monaco, and Uruguay, at 15.21 (95% UI: 12.4–18.51), 13.27 (95% UI: 8.78–19.3), and 12.55 (95% UI: 11.38–13.72) per 100,000 population, respectively. Between 1990 and 2021, 67.6% of countries and territories exhibited an increasing trend in ASIR. In terms of prevalence, Germany and Malaysia recorded the highest and lowest ASPRs in 2021, at 14.11 (95% UI: 12.98–15.17) and 2.04 (95% UI: 1.75–2.36) per 100,000 population, respectively. Overall, 75.5% of countries and territories showed an increasing trend in ASPR during the study period. Trends in mortality and DALYs varied by country, with 65.2% and 62.7% of countries and territories demonstrating increasing trends in the ASMR and ASDR, respectively. Further detailed findings and national-level analyses are available in the online **Supplementary Materials** (**Supplementary Tables S5–S8**).

### 3.3 Age and sex-specific trends

**Figure 3:**
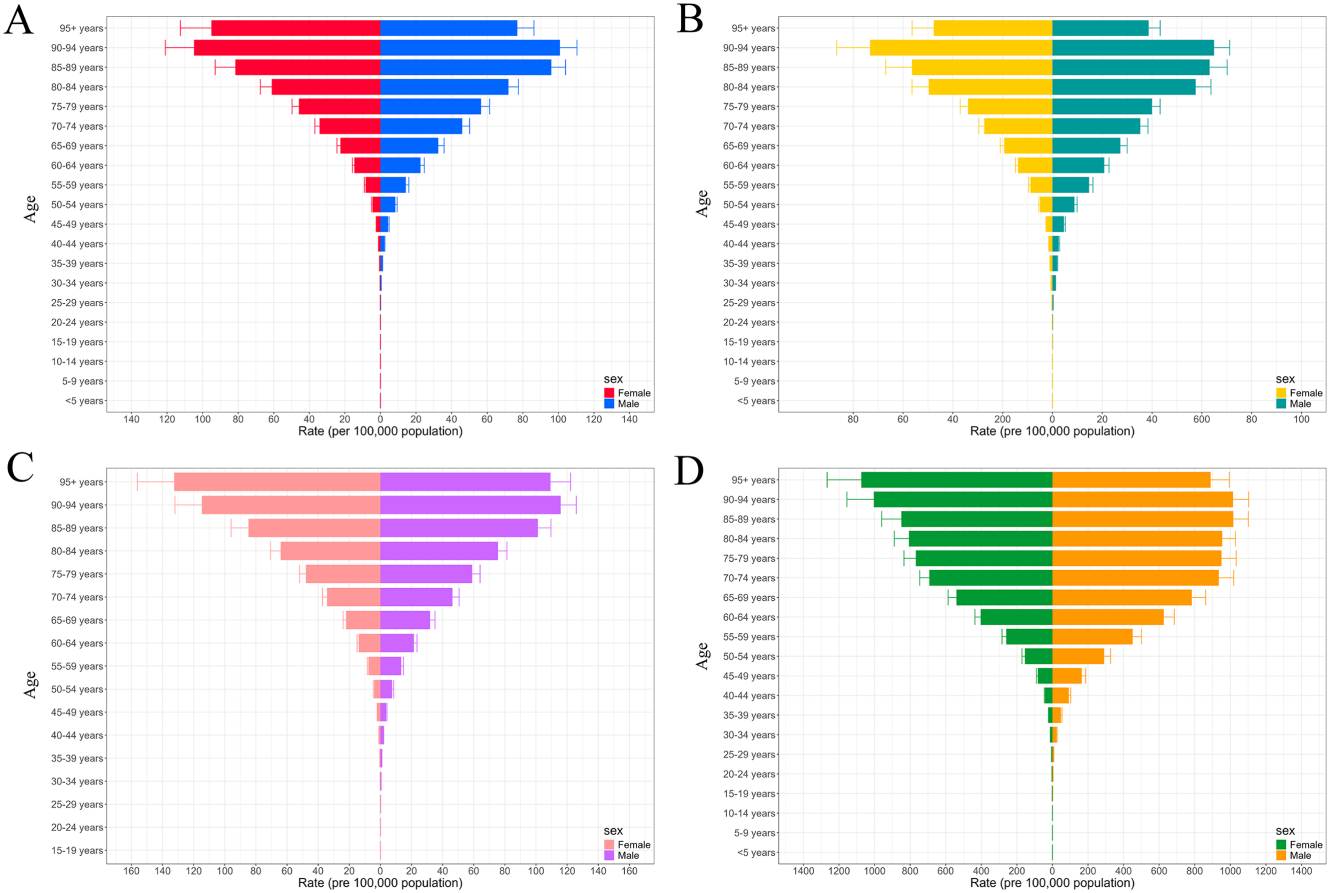
The trends in rate of incidence **(A)**, prevalence **(B)**, mortality **(C)**, and DALYs **(D)** for pancreatic cancer across different sexes by age groups ranging from under 5 years to 95+ years. DALYs, disability-adjusted life years.

In 2021, the global number of female individuals affected by pancreatic cancer was approximately 200,700.4 (95% UI: 176,302–216,717.9), with an estimated 234,915.6 new cases (95% UI: 205,148.7–255,434.6). Female deaths were approximately 235,714.7 (95% UI: 206,198.6–256,636.8), while total DALYs among females reached around 4,854,242.7 (95% UI: 4,346,324.1–5,249,520.9). Compared with females, male patients exhibited higher mortality (270,037.4, 95% UI: 247,469.9–295,172.9) and DALYs (6,462,720.6, 95% UI: 5,913,394.6–7,103,691.3) (**Supplementary Tables S9**, **S10**). In addition, incidence and prevalence rates displayed similar age-specific trends, rising with age and peaking in the 90–94 age group (**Figure 3**). With the exception of the 90–94 and 95+ age groups, males consistently showed higher incidence and prevalence rates than females across all other age groups (**Supplementary Tables S11**, **S12**). Furthermore, female mortality and DALYs rates also increased with age, reaching their highest levels in the 95+ age group. In contrast, male mortality and DALYs rates peaked in the 90–94 and 85–89 age groups, respectively (**Supplementary Tables S13**, **S14**). In terms of absolute numbers, the majority of incidence, prevalence, mortality, and DALYs cases were concentrated in the 65–69 and 70–74 age groups, with males consistently accounting for a greater share than females (**Supplementary Figure S6**).

### 3.4 Risk factors

**Figure 4:**
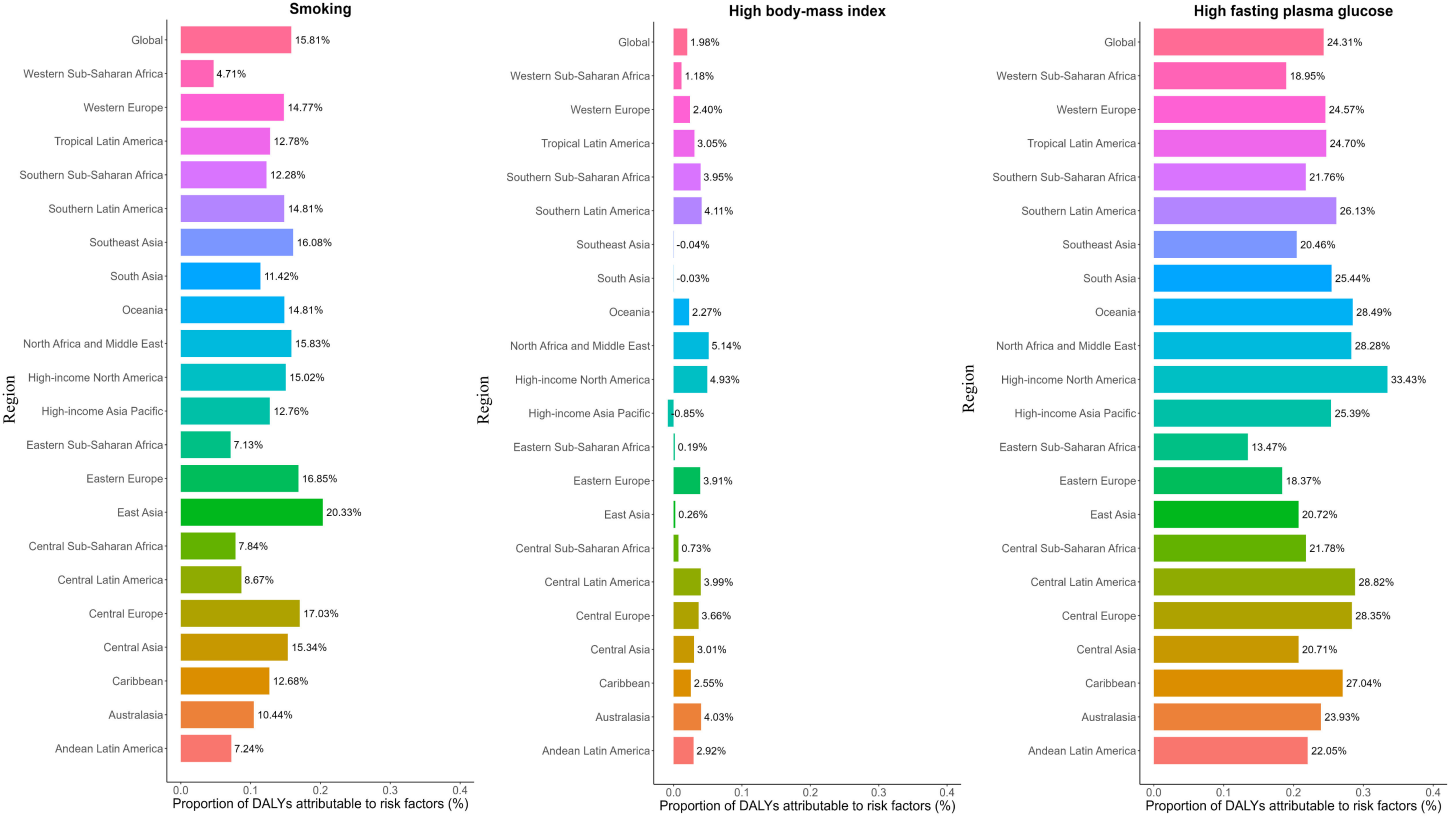
Percentage contribution of risk factors to all-age DALYs of pancreatic cancer in 2021, for both sexes, globally and by regions. DALYs, disability-adjusted life years.

**Figure 4** illustrates the contribution of three key risk factors to all-age DALYs due to pancreatic cancer across 21 GBD regions in 2021, for both sexes combined. Globally, high fasting plasma glucose (24.31%), smoking (15.81%), and high BMI (1.98%) were the primary contributors to pancreatic cancer DALYs, although the relative contribution of each risk factor varied by regional development status (**Supplementary Table S15**). For example, the burden attributable to smoking was highest in Eastern Europe (20.33%) and Central Europe (17.00%), where tobacco use remains prevalent, and lowest in Western Sub-Saharan Africa (4.71%). Similarly, the contribution of high fasting plasma glucose was greatest in High-income North America (33.43%) and lowest in Eastern Sub-Saharan Africa (13.47%). These differences likely reflect regional variations in exposure and health system capacity.

### 3.5 Cross-country inequalities

**Figure 5:**
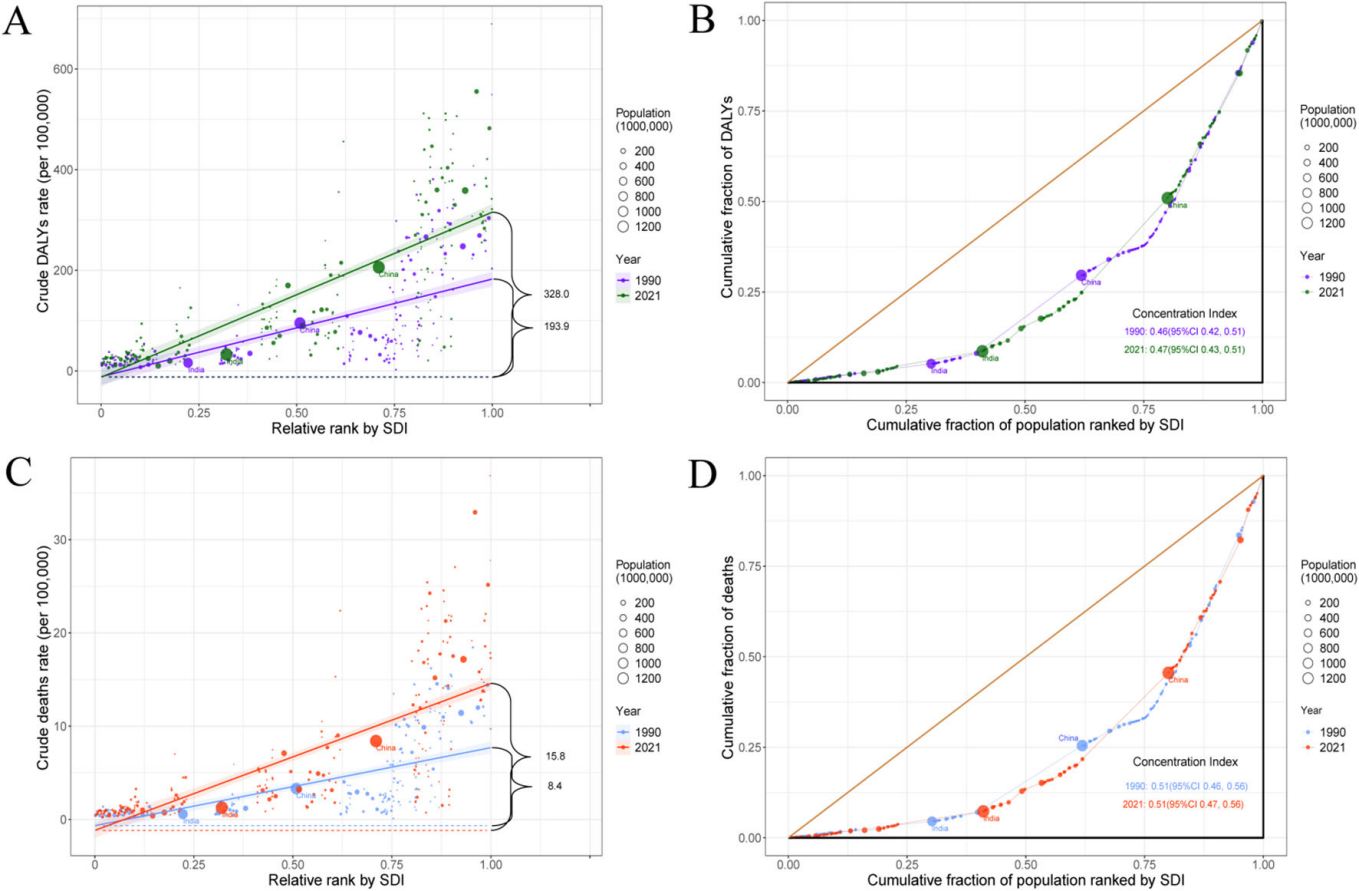
SDI-related health inequality slope index and concentration index for DALYs **(A, B)** and deaths **(C, D)** of pancreatic cancer worldwide, 1990 and 2021. SDI, socio-demographic index; DALYs, disability-adjusted life years.

A significant absolute and relative inequality in the burden of pancreatic cancer associated with SDI was observed, and both increased substantially over time (**Supplementary Table S16**). Notably, DALYs were disproportionately concentrated in countries with higher levels of sociodemographic development. According to the slope index of inequality, in 1990, the disparity in DALYs per 100,000 population between countries with the highest and lowest levels of SDI was 193.9 (95% CI: 162.0 to 225.9), which increased to 328.0 (95% CI: 290.7 to 365.2) by 2021 (**Figure 5A**). The concentration index which reflects relative inequality was 0.46 (95% CI: 0.42 to 0.51) in 1990 and 0.47 (95% CI: 0.43 to 0.51) in 2021, indicating a persistent imbalance in the distribution of disease burden across countries with varying SDI levels (**Figure 5B**). Similar patterns of inequality were also observed for pancreatic cancer mortality (**Figures 5C, D**).

## 4 Discussion

This study presents the most recent global, regional, and national data on the incidence, prevalence, mortality, and DALYs related to pancreatic cancer from 1990 to 2021. A comprehensive assessment was conducted across dimensions of age, sex, temporal trends, risk factors, and health inequalities. Joinpoint regression showed a marked global increase across all indicators, with incidence, prevalence, mortality, and DALYs more than doubling since 1990. Notably, despite this overall upward trajectory, there was a slight but encouraging decline in recent years, possibly influenced by disruptions related to the COVID-19 pandemic. Furthermore, age and sex analyses indicated that the burden of pancreatic cancer increases with age, with males exhibiting a higher burden than females. These demographic patterns should be considered when designing targeted screening and prevention strategies. Furthermore, our risk factor assessment identified high fasting plasma glucose was the leading modifiable risk factor, accounting for nearly one-quarter of DALYs globally. This underscores the importance of integrating metabolic control into cancer prevention and management. The findings also emphasize substantial and worsening socioeconomic disparities in pancreatic cancer burden, especially in regions with higher SDI. This underscores the necessity for oncologists and healthcare policymakers to prioritize early detection strategies and metabolic risk management programs, tailored specifically to high-risk populations and regions experiencing disproportionate disease burdens. Integrating targeted metabolic screening and early intervention protocols into clinical oncology practice may substantially mitigate the rising global burden of pancreatic cancer.

Consistent with prior studies, our findings confirm a strong association between pancreatic cancer incidence and socioeconomic development. High-income regions bear the greatest burden, reflecting aging populations and improved diagnostic capacity. Higher exposure to risk factors and broader access to early screening likely contribute to the greater burden observed in high-SDI regions. Firstly, populations in high-income regions generally place a higher priority on personal health, including early diagnostic practices, whereas low- and middle-income countries often lack access to effective diagnostic tools (19–21). Secondly, high-income countries often show higher prevalence of smoking and metabolic syndrome, driven by aging demographics and lifestyle factors. For example, the prevalence of central obesity is 44.7% in high-income countries compared to 30.6% in low-income countries (22). In addition, we observed a general upward trend in the burden of pancreatic cancer over the past three decades, particularly in low and low-middle SDI regions. This trend underscores disparities in the formulation and implementation of cancer prevention strategies across countries. Specifically, smoking is a major risk factor for pancreatic cancer, and bans and health education related to smoking behaviors have been shown to be effective in reducing the incidence of pancreatic cancer (23). As early as 2009, the WHO recommended that countries adopt comprehensive smoke-free policies, but such tobacco control measures were implemented only in a small number of high- and middle-income countries. According to the latest edition of the 2023 WHO report on the global tobacco epidemic, tobacco control policies in some low-income countries remain insufficient due to weak policy enforcement and limited public health awareness (24, 25). Moreover, constrained health expenditures significantly hinder cancer control efforts in low- and middle-income countries. For example, in 2021, the average per capita health expenditure was approximately USD 4,000 in high-income countries, compared to just USD 45 in low-income countries, highlighting a stark disparity in available resources for cancer prevention and care (26). Limited investment restricts access to high-quality screening and treatment, leading to missed opportunities for early diagnosis and timely care. Furthermore, a potential contributing factor to the rising incidence of pancreatic cancer is the increased detection of pancreatic cystic neoplasms—a disease entity that has gained clinical attention over the past two decades. Advances in imaging technology have led to greater recognition of these lesions, which may partially account for the upward trend in reported incidence (27–29).

Age is a well-established non-modifiable risk factor, and pancreatic cancer predominantly affects older adults. Although pancreatic cancer remains exceedingly rare in individuals under 40 years of age, its incidence increases markedly thereafter, reflecting the cumulative exposure to both intrinsic aging processes and long-term environmental and metabolic risk factors (5). With rising global life expectancy, population aging is expected to markedly increase the number of individuals at risk. According to the United Nations’ World Population Prospects 2019, the number of people aged 65 years and older is expected to double by 2050, reaching 1.5 billion (30). This demographic group represents the population segment at the highest risk for pancreatic cancer. Importantly, this demographic shift is not confined to high-income countries. In regions such as sub-Saharan Africa, where the current incidence of pancreatic cancer remains low largely due to underdiagnosis and limited healthcare infrastructure, the proportion of older adults is expected to rise substantially in the coming decades. As diagnostic capacity improves alongside population aging, these regions may experience a delayed yet significant increase in pancreatic cancer incidence. A consistent sex disparity was also observed, with males showing higher incidence, mortality, and DALYs than females. Several plausible mechanisms may underlie this persistent sex difference. From a biological perspective, female sex hormones (especially estrogen) may be protective against the development of pancreatic cancer. Experimental evidence suggests that estrogen may inhibit tumor proliferation through G-protein-coupled estrogen receptor activation, potentially enhancing immune response (31, 32). These hormonal influences may partially explain the lower burden observed among females, especially during their premenopausal years. Behavioral and environmental exposures also likely contribute. Males generally exhibit higher rates of tobacco smoking and excessive alcohol consumption, both of which are well-established risk factors for pancreatic cancer. Occupational exposures to carcinogens, more common in male-dominated industries, may further compound this risk.

Our analysis confirmed smoking, high fasting plasma glucose, and high BMI as the major modifiable risk factors, consistent with previous studies (5). Notably, the leading contributor has shifted from smoking (GBD 2017) to high fasting plasma glucose (GBD 2021), reflecting the global decline in smoking and the growing burden of metabolic disorders (5). This shift likely reflects the global decline in smoking prevalence over recent decades. Between 1990 and 2015, the ASPR of daily smoking decreased by 28.4% for males and 34.4% for females globally (33). In high-income countries such as the United States, long-term declines in smoking rates have contributed to recent stabilization or modest declines in pancreatic cancer incidence (34). This contrasts with trends observed in many low- and middle-income countries, especially in parts of Asia such as China and India, where smoking prevalence remains high or is increasing among certain groups. For example, in China, the smoking rate among men remains close to 50% (35), and our findings indicate a substantial increase in both the incidence and mortality of pancreatic cancer from 1990 to 2021. These patterns underscore the importance of tobacco control. Additionally, we found that in 2021, 24.31% of pancreatic cancer DALYs were attributable to high fasting plasma glucose. According to the United States National Cancer Institute, diabetes is associated with a 1.8-fold increased risk of pancreatic cancer (36). Another study reported that each 0.56 mmol/L increase in fasting plasma glucose corresponds to a 14% increase in pancreatic cancer incidence (37). Over the past few decades, the global prevalence of diabetes has risen from 4.3% in 1980 to 9.0% in 2014, with the number of adults living with diabetes nearly quadrupling (38). These trends indicate that diabetes will likely play an increasingly important role in the future burden of pancreatic cancer. High BMI is another important modifiable risk factor. Obesity is associated with dysfunctional adipose tissue, adipocyte death, and chronic low-grade inflammation, all of which contribute to a tumor-promoting microenvironment (39). The global prevalence of obesity (BMI ≥30 kg/m²) is rising rapidly. By 2025, it is projected that 18% of men and over 21% of women will be classified as obese, while severe obesity may affect more than 6% of men and 9% of women (40). The distribution of high BMI also reflects patterns of socioeconomic development: regions such as Europe, high-income North America, and Australasia show higher rates of overweight and obesity, whereas high-income Asia Pacific countries bear a relatively lower burden (41). Overall, both genetic predisposition and modifiable risk factors, whether acting independently or synergistically, play critical roles in the development of pancreatic cancer. As detection capabilities continue to advance, understanding the complex interplay among these risk factors will be essential for guiding preventive strategies. This includes primary prevention efforts aimed at reducing exposure and identifying populations at highest risk of developing this typically fatal disease.

This study has several limitations. First, data from low-income countries are often incomplete, which may lead to reporting bias. In particular, the COVID-19 pandemic had a profound impact on healthcare systems, diagnostic practices, and disease reporting. These disruptions may have resulted in delayed diagnoses and altered disease burden estimates, thereby influencing the observed trends. These disruptions should be considered when interpreting recent trends. Second, although pancreatic cancer comprises multiple pathological subtypes and clinical stages, this study evaluated only the overall burden of pancreatic cancer. Future research should explore the burden associated with different histological subtypes and disease stages. Finally, our analysis focused on three major modifiable risk factors, namely smoking, high BMI, and high fasting plasma glucose, while other potentially relevant environmental and genetic factors were not included. These unmeasured variables may also play important roles and warrant further investigation.

## 5 Conclusion

Over the past three decades, the global burden of pancreatic cancer has shown a consistent upward trend, particularly in developed regions such as High-income Asia Pacific, Central Europe, and Western Europe. The primary risk factors for pancreatic cancer, including smoking, diabetes, and obesity, are modifiable and therefore present a valuable opportunity for prevention. In light of this growing burden, preventive strategies should prioritize the control of modifiable risk factors through globally coordinated efforts. These efforts should include comprehensive tobacco control policies and targeted interventions to reduce the prevalence of obesity and diabetes.

## Data Availability

The original contributions presented in the study are included in the article/**Supplementary Material**. Further inquiries can be directed to the corresponding authors.
