# Supplementary material for: Global, regional, and national burden and trends of pancreatic cancer, 1990–2021: a systematic analysis for the Global Burden of Disease Study 2021
Source: Front Oncol. 2025 Nov 3;15:1671856. doi: 10.3389/fonc.2025.1671856 (PMC12620227; doi:10.3389/fonc.2025.1671856)
Supplement: Supplementary file 1 [file DataSheet1.docx]

**Supplementary materials**

**Abbreviations**

AAPC: Average annual percentage change

APC: Annual percentage change

ASR: Age-standardized rate

ASDR: Age-standardized DALYs rate

ASIR: Age-standardized incidence rate

ASMR: Age-standardized mortality rate

ASPR: Age-standardized prevalence rate

BMI: Body mass index

CI: Confidence interval

DALYs: Disability-adjusted life years

GBD: Global Burden of Disease

ICD: International Classification of Diseases

SDI: Socio-demographic index

UI: Uncertainty intervals

**Contents**

[Table S1. List of ICD codes and description corresponding to pancreatic cancer in GBD 2021. 4](#_Toc5109)

[Table S2. Prevalence case numbers and ASRs of pancreatic cancer in 1990 and 2021, by sex, across 5 SDI regions and 21 GBD regions, with AAPC estimates from 1990 to 2021. 6](#_Toc4353)

[Table S3. Mortality case numbers and ASRs of pancreatic cancer in 1990 and 2021, by sex, across 5 SDI regions and 21 GBD regions, with AAPC estimates from 1990 to 2021. 9](#_Toc20677)

[Table S4. DALYs and ASRs of pancreatic cancer in 1990 and 2021, by sex, across 5 SDI regions and 21 GBD regions, with AAPC estimates from 1990 to 2021. 12](#_Toc10611)

[Table S5. Case numbers and ASRs of incidence of pancreatic cancer in 1990 and 2021 for both sexes in 204 countries and AAPC from 1990 to 2021. 15](#_Toc24939)

[Table S6. Case numbers and ASRs of prevalence of pancreatic cancer in 1990 and 2021 for both sexes in 204 countries and AAPC from 1990 to 2021. 32](#_Toc5546)

[Table S7. Case numbers and ASRs of mortality of pancreatic cancer in 1990 and 2021 for both sexes in 204 countries and AAPC from 1990 to 2021. 49](#_Toc15847)

[Table S8. Case numbers and ASRs of DALYs of pancreatic cancer in 1990 and 2021 for both sexes in 204 countries and AAPC from 1990 to 2021. 66](#_Toc10740)

[Table S9. Case numbers and ASRs of incidence and prevalence of pancreatic cancer from 1990 to 2021 for both sexes globally. 83](#_Toc23663)

[Table S10. Case numbers and ASRs of mortality and DALYs of pancreatic cancer from 1990 to 2021 for both sexes globally. 86](#_Toc12871)

[Table S11. Age distribution of pancreatic cancer case numbers and rates per 100,000 population of incidence by sex in 2021. 89](#_Toc13653)

[Table S12. Age distribution of pancreatic cancer case numbers and rates per 100,000 population of prevalence by sex in 2021. 91](#_Toc31000)

[Table S13. Age distribution of pancreatic cancer case numbers and rates per 100,000 population of mortality by sex in 2021. 93](#_Toc30461)

[Table S14. Age distribution of pancreatic cancer case numbers and rates per 100,000 population of DALYs by sex in 2021. 95](#_Toc20978)

[Table S15. Case numbers and percentage contribution of risk factors to all-age DALYs of pancreatic cancer in 2021, for both sexes, globally and by regions. 97](#_Toc29331)

[Table S16. The slope index of inequality and concentration index of pancreatic cancer mortality and DALYs related to SDI. 100](#_Toc11742)

[Figure S1. The APC and AAPC of ASR for incidence (A), prevalence (B), mortality (C) and DALYs (D) pancreatic cancer at the global level based on the joinpoint analysis. 101](#_Toc20324)

[Figure S2. The ASRs burden of pancreatic cancer incidence (A) and prevalence (C) in 2021 across the 21 GBD regions globally and their AAPCs (B, D) from 1990 to 2021. 102](#_Toc12240)

[Figure S3. The ASRs burden of pancreatic cancer mortality (A) and DALYs (C) in 2021 across the 21 GBD regions globally and their AAPCs (B, D) from 1990 to 2021. 103](#_Toc16899)

[Figure S4. The numbers of incidence (A), prevalence (B), mortality (C), and DALYs (D) for pancreatic cancer worldwide in 2021. 104](#_Toc10678)

[Figure S5. The ASRs of incidence (A), prevalence (B), mortality (C), and DALYs (D) for pancreatic cancer worldwide in 2021. 105](#_Toc11687)

[Figure S6. The trends in numbers of incidence (A), prevalence (B), mortality (C), and DALYs (D) for pancreatic cancer across different genders by age groups ranging from under 5 years to 95+ years. 106](#_Toc28389)

# Table S1. List of ICD codes and description corresponding to pancreatic cancer in GBD 2021.

| **ICD10 Code** | **Carcinoma** |  | **ICD9 Code** | **Carcinoma** |
| --- | --- | --- | --- | --- |
| C25 | Malignant neoplasm of pancreas |  | 157 | Malignant neoplasm of pancreas |
| C25.0 | Malignant neoplasm of head of pancreas |  | 157.0 | Malignant neoplasm of head of pancreas |
| C25.1 | Malignant neoplasm of body of pancreas |  | 157.1 | Malignant neoplasm of body of pancreas |
| C25.2 | Malignant neoplasm of tail of pancreas |  | 157.2 | Malignant neoplasm of tail of pancreas |
| C25.3 | Malignant neoplasm of pancreatic duct |  | 157.3 | Malignant neoplasm of pancreatic duct |
| C25.4 | Malignant neoplasm of endocrine pancreas |  | 157.4 | Malignant neoplasm of islets of langerhans |
| C25.7 | Malignant neoplasm of other parts of pancreas |  | 157.8 | Malignant neoplasm of other specified sites of pancreas |
| C25.8 | Malignant neoplasm of overlapping sites of pancreas |  | 157.9 | Malignant neoplasm of pancreas, part unspecified |
| C25.9 | Malignant neoplasm of pancreas, unspecified |  |  |  |

*Abbreviations: ICD, international classification of diseases; GBD, Global Burden of Disease.*

# **Table S2.** Prevalence case numbers and ASRs of pancreatic cancer in 1990 and 2021, by sex, across 5 SDI regions and 21 GBD regions, with AAPC estimates from 1990 to 2021.

| **Location** | **Prevalence** | | | | |
| --- | --- | --- | --- | --- | --- |
|  | **1990** | | **2021** | | **1990-2021** |
|  | **Number**  **(95 % UI)** | **ASR**  **(95 % UI)** | **Number**  **(95 % UI)** | **ASR**  **(95 % UI)** | **AAPC**  **(95% CI)** |
| **Global** | 172808.7 (164345.8-180916.4) | 4.39 (4.15-4.60) | 439000.6 (401738.8-471000.0) | 5.12 (4.66-5.50) | 0.49^*^ (0.36 to 0.61) |
| **SDI regions** |  |  |  |  |  |
| **High SDI** | 82918.6 (79110.9-85552.8) | 7.54 (7.20-7.77) | 205269.7 (184642.2-218778.6) | 9.89 (9.05-10.45) | 0.87^*^ (0.76 to 0.97) |
| **High-middle SDI** | 53742.1 (50834.9-56703.1) | 5.32 (5.03-5.61) | 118826.0 (106487.2-131768.9) | 6.04 (5.41-6.68) | 0.41^*^ (0.29 to 0.54) |
| **Middle SDI** | 26835.0 (24437.5-29568.4) | 2.45 (2.24-2.69) | 83175.1 (73143.6-93872.0) | 3.03 (2.68-3.42) | 0.69^*^ (0.59 to 0.78) |
| **Low-middle SDI** | 6766.7 (5731.2-7912.9) | 1.06 (0.90-1.23) | 24972.2 (23155.7-27001.5) | 1.68 (1.56-1.82) | 1.53^*^ (1.4 to 1.65) |
| **Low SDI** | 2319.7 (1800.5-2782.0) | 0.98 (0.76-1.17) | 6332.4 (5229.4-7657.5) | 1.19 (0.99-1.43) | 0.65^*^ (0.59 to 0.72) |
| **GBD regions** |  |  |  |  |  |
| **Andean Latin America** | 675.9 (562.4-794.8) | 3.25 (2.72-3.82) | 2316.9 (1788.3-2917.0) | 3.91 (3.01-4.92) | 0.64^*^ (0.14 to 1.14) |
| **Australasia** | 1610.8 (1529.1-1683.9) | 6.87 (6.51-7.18) | 4946.1 (4444.9-5342.6) | 9.27 (8.46-9.95) | 0.97^*^ (0.76 to 1.18) |
| **Caribbean** | 895.9 (840.2-952.4) | 3.44 (3.23-3.65) | 2132.7 (1876.8-2411.3) | 3.96 (3.49-4.48) | 0.47^*^ (0.22 to 0.72) |
| **Central Asia** | 1167.2 (1030.4-1352.8) | 2.37 (2.09-2.76) | 2846.5 (2500.8-3198.7) | 3.32 (2.92-3.72) | 1.14^*^ (0.55 to 1.74) |
| **Central Europe** | 9440.3 (9064.5-9778.8) | 6.27 (6.02-6.50) | 15647.9 (14349.5-16966.6) | 7.28 (6.68-7.90) | 0.49^*^ (0.31 to 0.66) |
| **Central Latin America** | 2791.8 (2722.0-2854.2) | 3.26 (3.17-3.35) | 8887.6 (7967.5-9876.8) | 3.51 (3.14-3.90) | 0.22 (-0.01 to 0.44) |
| **Central Sub-Saharan Africa** | 384.6 (306.2-470.3) | 1.62 (1.31-1.96) | 1001.8 (695.6-1389.3) | 1.70 (1.18-2.36) | 0.16^*^ (0.03 to 0.29) |
| **East Asia** | 32771.1 (27633.6-38213.5) | 3.53 (2.99-4.11) | 99055.9 (79103.1-120447.8) | 4.53 (3.64-5.49) | 0.83^*^ (0.68 to 0.97) |
| **Eastern Europe** | 16231.3 (15424.7-17357.9) | 5.75 (5.46-6.15) | 22724.8 (20951.8-24669.2) | 6.63 (6.11-7.18) | 0.57^*^ (0.27 to 0.86) |
| **Eastern Sub-Saharan Africa** | 952.3 (738.0-1164.1) | 1.22 (0.95-1.49) | 2670.9 (2159.2-3449.0) | 1.50 (1.22-1.90) | 0.67^*^ (0.58 to 0.75) |
| **High-income Asia Pacific** | 17307.7 (16408.1-18058.5) | 8.66 (8.17-9.06) | 55461.0 (46296.4-61060.8) | 11.20 (9.69-12.17) | 0.81^*^ (0.71 to 0.9) |
| **High-income North America** | 28324.9 (26851.7-29219.2) | 8.17 (7.79-8.41) | 62155.1 (57205.4-64930.3) | 9.67 (8.98-10.08) | 0.51^*^ (0.39 to 0.63) |
| **North Africa and Middle East** | 3824.5 (3144.4-4527.3) | 2.18 (1.78-2.57) | 15885.8 (13916.8-17926.2) | 3.43 (3.01-3.86) | 1.49^*^ (1.4 to 1.58) |
| **Oceania** | 42.0 (32.9-53.7) | 1.37 (1.10-1.75) | 135.3 (109.0-172.3) | 1.72 (1.40-2.17) | 0.72^*^ (0.6 to 0.84) |
| **South Asia** | 4665.2 (3680.2-5634.6) | 0.76 (0.60-0.92) | 16349.5 (14469.9-18079.1) | 1.08 (0.96-1.19) | 1.13^*^ (1.01 to 1.26) |
| **Southeast Asia** | 4686.2 (4030.9-5380.9) | 1.71 (1.47-1.96) | 17948.0 (15469.7-20857.7) | 2.63 (2.27-3.05) | 1.41^*^ (1.36 to 1.46) |
| **Southern Latin America** | 3060.6 (2893.8-3252.6) | 6.60 (6.23-7.00) | 5739.5 (5335.2-6108.5) | 6.63 (6.18-7.07) | 0.03 (-0.19 to 0.24) |
| **Southern Sub-Saharan Africa** | 812.2 (704.5-984.6) | 2.89 (2.48-3.55) | 2562.4 (2261.3-2845.0) | 4.31 (3.79-4.76) | 1.3^*^ (0.97 to 1.63) |
| **Tropical Latin America** | 3324.6 (3189.9-3429.0) | 3.55 (3.39-3.67) | 11681.6 (10991.0-12194.5) | 4.51 (4.22-4.71) | 0.8^*^ (0.51 to 1.09) |
| **Western Europe** | 39171.7 (37292.2-40842.4) | 6.93 (6.62-7.21) | 85952.5 (78065.2-91846.1) | 9.74 (8.99-10.33) | 1.1^*^ (0.96 to 1.23) |
| **Western Sub-Saharan Africa** | 668.1 (568.0-771.9) | 0.74 (0.63-0.85) | 2898.9 (2383.9-3410.4) | 1.42 (1.18-1.64) | 2.13^*^ (2.08 to 2.19) |

*Abbreviations: ASR, age-standardized rate; SDI, sociodemographic index; GBD, Global Burden of Disease; AAPC, average annual percentage change; UI, uncertainty interval; CI, confidence interval.*

# **Table S3.** Mortality case numbers and ASRs of pancreatic cancer in 1990 and 2021, by sex, across 5 SDI regions and 21 GBD regions, with AAPC estimates from 1990 to 2021.

| **Location** | **Mortality** | | | | |
| --- | --- | --- | --- | --- | --- |
|  | **1990** | | **2021** | | **1990-2021** |
|  | **Number**  **(95 % UI)** | **ASR**  **(95 % UI)** | **Number**  **(95 % UI)** | **ASR**  **(95 % UI)** | **AAPC**  **(95% CI)** |
| **Global** | 211612.7 (199990.3-221951.0) | 5.66 (5.33-5.93) | 505752.2 (461224.4-543899.4) | 5.95 (5.40-6.41) | 0.16^*^ (0.06 to 0.27) |
| **SDI regions** |  |  |  |  |  |
| **High SDI** | 97179.5 (91895.6-100353.4) | 8.70 (8.22-8.98) | 206593.2 (184625.8-220561.5) | 9.37 (8.52-9.96) | 0.23^*^ (0.13 to 0.33) |
| **High-middle SDI** | 68842.5 (65128.3-72746.2) | 7.09 (6.69-7.50) | 151079.9 (135113.2-166837.2) | 7.61 (6.80-8.40) | 0.26^*^ (0.14 to 0.37) |
| **Middle SDI** | 33583.8 (30682.5-36872.1) | 3.42 (3.14-3.72) | 106766.5 (94341.7-119905.9) | 4.06 (3.60-4.54) | 0.58^*^ (0.46 to 0.7) |
| **Low-middle SDI** | 8671.5 (7355.3-10120.7) | 1.50 (1.27-1.74) | 32553.1 (30234.8-35163.5) | 2.35 (2.17-2.53) | 1.47^*v^ (1.32 to 1.61) |
| **Low SDI** | 3029.1 (2350.8-3633.5) | 1.41 (1.10-1.70) | 8198.6 (6799.6-9862.4) | 1.73 (1.45-2.07) | 0.67^*^ (0.58 to 0.76) |
| **GBD regions** |  |  |  |  |  |
| **Andean Latin America** | 930.5 (784.1-1096.2) | 4.78 (4.04-5.63) | 3216.3 (2487.3-4027.2) | 5.56 (4.30-6.97) | 0.52^*^ (0.04 to 0.99) |
| **Australasia** | 1747.0 (1660.7-1822.5) | 7.41 (7.03-7.75) | 4327.4 (3860.6-4637.7) | 7.73 (6.97-8.24) | 0.1 (-0.28 to 0.49) |
| **Caribbean** | 1282.5 (1205.0-1361.9) | 5.08 (4.77-5.38) | 2919.9 (2574.9-3301.7) | 5.40 (4.76-6.11) | 0.19 (-0.08 to 0.47) |
| **Central Asia** | 1473.4 (1295.1-1715.7) | 3.16 (2.77-3.71) | 3596.0 (3169.5-4041.1) | 4.53 (4.01-5.08) | 1.21^*^ (0.74 to 1.67) |
| **Central Europe** | 12841.1 (12286.5-13313.7) | 8.66 (8.28-8.99) | 21977.5 (20086.5-23743.0) | 9.72 (8.91-10.52) | 0.38^*^ (0.2 to 0.56) |
| **Central Latin America** | 3722.3 (3610.7-3818.5) | 4.74 (4.58-4.87) | 11918.2 (10667.3-13218.6) | 4.83 (4.33-5.36) | 0.05 (-0.18 to 0.27) |
| **Central Sub-Saharan Africa** | 493.4 (397.8-603.2) | 2.36 (1.93-2.85) | 1248.6 (862.7-1745.5) | 2.46 (1.68-3.49) | 0.13^*^ (0.03 to 0.22) |
| **East Asia** | 40114.6 (33980.7-46655.9) | 4.79 (4.09-5.56) | 123819.5 (99918.4-149380.5) | 5.72 (4.63-6.87) | 0.59^*^ (0.37 to 0.8) |
| **Eastern Europe** | 20666.1 (19658.4-21939.0) | 7.37 (7.00-7.83) | 30117.6 (27830.8-32639.7) | 8.52 (7.88-9.23) | 0.56^*^ (0.29 to 0.83) |
| **Eastern Sub-Saharan Africa** | 1250.0 (969.0-1525.2) | 1.78 (1.39-2.18) | 3416.6 (2783.0-4358.4) | 2.18 (1.80-2.75) | 0.65^*^ (0.57 to 0.72) |
| **High-income Asia Pacific** | 17589.4 (16680.6-18265.5) | 8.93 (8.41-9.29) | 49615.9 (41694.5-54352.8) | 9.56 (8.34-10.34) | 0.19^*^ (0.03 to 0.35) |
| **High-income North America** | 31853.9 (29802.6-32996.7) | 8.91 (8.37-9.22) | 63038.5 (57497.5-66152.9) | 9.32 (8.56-9.75) | 0.12 (-0.05 to 0.3) |
| **North Africa and Middle East** | 4868.3 (3971.9-5751.3) | 3.09 (2.50-3.64) | 19785.2 (17372.7-22240.5) | 4.69 (4.12-5.26) | 1.37^*^ (1.19 to 1.56) |
| **Oceania** | 52.7 (41.7-67.5) | 2.01 (1.62-2.55) | 167.8 (136.1-214.1) | 2.46 (2.00-3.12) | 0.65^*^ (0.54 to 0.76) |
| **South Asia** | 5923.8 (4666.5-7178.7) | 1.07 (0.84-1.29) | 21593.0 (19209.5-23811.3) | 1.51 (1.35-1.67) | 1.14^*^ (0.96 to 1.33) |
| **Southeast Asia** | 5790.6 (4994.9-6651.3) | 2.35 (2.03-2.70) | 22413.6 (19334.7-26000.6) | 3.53 (3.04-4.10) | 1.33^*^ (1.22 to 1.45) |
| **Southern Latin America** | 4364.0 (4096.6-4641.2) | 9.58 (8.99-10.20) | 8097.6 (7453.5-8633.2) | 9.13 (8.42-9.72) | -0.15 (-0.36 to 0.06) |
| **Southern Sub-Saharan Africa** | 1056.3 (902.6-1308.3) | 4.12 (3.51-5.16) | 3386.1 (2979.9-3744.4) | 6.22 (5.49-6.86) | 1.37^*^ (1.06 to 1.68) |
| **Tropical Latin America** | 4417.2 (4210.3-4567.5) | 5.15 (4.85-5.35) | 15957.5 (14734.4-16749.3) | 6.27 (5.76-6.59) | 0.67^*^ (0.36 to 0.97) |
| **Western Europe** | 50274.6 (47367.2-52445.7) | 8.52 (8.05-8.88) | 91365.6 (81860.9-97826.1) | 9.26 (8.43-9.84) | 0.28^*^ (0.2 to 0.35) |
| **Western Sub-Saharan Africa** | 901.3 (772.2-1036.5) | 1.09 (0.93-1.25) | 3774.0 (3151.5-4388.1) | 2.09 (1.77-2.42) | 2.13^*^ (2.05 to 2.21) |

*Abbreviations: ASR, age-standardized rate; SDI, sociodemographic index; GBD, Global Burden of Disease; AAPC, average annual percentage change; UI, uncertainty interval; CI, confidence interval.*

# **Table S4.** DALYs and ASRs of pancreatic cancer in 1990 and 2021, by sex, across 5 SDI regions and 21 GBD regions, with AAPC estimates from 1990 to 2021.

| **Location** | **DALYs** | | | | |
| --- | --- | --- | --- | --- | --- |
|  | **1990** | | **2021** | | **1990-2021** |
|  | **Number**  **(95 % UI)** | **ASR**  **(95 % UI)** | **Number**  **(95 % UI)** | **ASR**  **(95 % UI)** | **AAPC**  **(95% CI)** |
| **Global** | 5210478.7 (4967404.9-5481660.6) | 129.32 (122.98-135.98) | 11316963.4 (10464696.9-12169335.9) | 130.33 (120.52-140.13) | 0.02 (-0.07 to 0.12) |
| **SDI regions** |  |  |  |  |  |
| **High SDI** | 2148520.4 (2062652.9-2203707.9) | 197.13 (189.39-202.05) | 4053784.3 (3742274.5-4283638.6) | 202.04 (188.27-212.61) | 0.07 (-0.03 to 0.18) |
| **High-middle SDI** | 1783424.0 (1682491.7-1890335.3) | 174.68 (164.79-185.04) | 3486678.8 (3118892.7-3861181.0) | 176.70 (158.07-195.43) | 0.04 (-0.1 to 0.19) |
| **Middle SDI** | 944707.7 (858505.5-1044034.0) | 84.60 (77.10-92.93) | 2684710.7 (2362750.0-3027671.9) | 96.61 (85.18-108.88) | 0.43^*^ (0.34 to 0.52) |
| **Low-middle SDI** | 241906.3 (204321.9-283539.0) | 36.99 (31.34-43.21) | 855268.0 (792107.8-926182.9) | 56.78 (52.66-61.42) | 1.4^*^ (1.3 to 1.5) |
| **Low SDI** | 84472.6 (65503.3-101441.4) | 34.82 (27.01-41.70) | 224046.8 (184706.5-272777.8) | 41.21 (34.19-49.79) | 0.55^*^ (0.49 to 0.61) |
| **GBD regions** |  |  |  |  |  |
| **Andean Latin America** | 23368.7 (19361.3-27425.4) | 110.79 (92.10-130.15) | 74699.2 (57561.5-93984.9) | 125.21 (96.38-157.38) | 0.43 (-0.07 to 0.92) |
| **Australasia** | 38526.9 (36829.1-40154.9) | 164.74 (157.34-171.52) | 85063.2 (78185.6-90378.6) | 164.91 (153.05-175.07) | -0.01 (-0.39 to 0.38) |
| **Caribbean** | 29511.0 (27572.0-31442.7) | 112.40 (105.01-119.69) | 68060.4 (59618.8-77425.5) | 126.50 (110.70-144.06) | 0.41^*^ (0.14 to 0.68) |
| **Central Asia** | 40751.3 (35992.8-47069.1) | 82.67 (72.92-95.77) | 97305.1 (85156.0-109802.6) | 111.64 (97.94-125.60) | 1.02^*^ (0.47 to 1.58) |
| **Central Europe** | 319916.9 (307314.3-331300.9) | 211.89 (203.47-219.53) | 481394.7 (442737.8-521330.0) | 227.27 (208.75-246.43) | 0.23^*^ (0.03 to 0.44) |
| **Central Latin America** | 95747.0 (93525.2-97835.1) | 110.06 (107.26-112.70) | 289852.5 (260613.5-321737.2) | 113.65 (102.01-126.19) | 0.06 (-0.25 to 0.37) |
| **Central Sub-Saharan Africa** | 14126.2 (11275.9-17396.0) | 57.94 (47.03-70.38) | 36109.1 (24941.0-50577.6) | 59.43 (41.04-83.01) | 0.08 (-0.05 to 0.21) |
| **East Asia** | 1155643.7 (974050.0-1347640.7) | 122.31 (103.25-142.29) | 3032992.9 (2404587.3-3684550.0) | 137.21 (109.16-166.03) | 0.38^*^ (0.21 to 0.55) |
| **Eastern Europe** | 552767.1 (526183.9-590425.5) | 195.95 (186.46-209.44) | 723795.9 (666446.6-786751.6) | 212.43 (195.58-230.76) | 0.37^*^ (0.07 to 0.67) |
| **Eastern Sub-Saharan Africa** | 34756.3 (26870.1-42510.2) | 43.35 (33.59-53.07) | 94803.5 (76368.3-123274.7) | 51.69 (41.97-66.27) | 0.57^*^ (0.5 to 0.64) |
| **High-income Asia Pacific** | 407045.6 (391117.2-421297.8) | 199.08 (191.03-206.30) | 864942.8 (760424.5-931628.8) | 197.60 (178.87-210.60) | -0.03 (-0.13 to 0.07) |
| **High-income North America** | 692391.0 (661106.9-710740.9) | 202.85 (194.36-207.98) | 1309238.8 (1232008.1-1359961.4) | 205.41 (194.75-212.77) | 0 (-0.17 to 0.18) |
| **North Africa and Middle East** | 133124.5 (109673.9-158604.8) | 74.28 (60.93-88.16) | 506969.9 (444632.8-573272.7) | 106.74 (93.76-120.32) | 1.18^*^ (1.1 to 1.26) |
| **Oceania** | 1506.2 (1174.3-1959.5) | 47.84 (37.93-61.42) | 4744.3 (3789.1-6109.2) | 58.56 (47.49-74.75) | 0.65^*^ (0.54 to 0.76) |
| **South Asia** | 168440.4 (132874.3-203932.6) | 26.94 (21.24-32.56) | 561188.5 (496660.8-621786.4) | 36.38 (32.29-40.20) | 0.98^*^ (0.87 to 1.1) |
| **Southeast Asia** | 165073.7 (142221.4-189672.7) | 59.32 (51.06-68.04) | 597956.0 (515406.9-697938.1) | 85.92 (73.82-99.74) | 1.21^*^ (1.16 to 1.25) |
| **Southern Latin America** | 101652.2 (96226.3-107999.7) | 217.82 (206.07-231.18) | 176421.9 (165592.4-187883.8) | 205.04 (192.65-218.30) | -0.19 (-0.42 to 0.05) |
| **Southern Sub-Saharan Africa** | 28498.9 (24725.1-34486.3) | 99.65 (85.59-122.16) | 89509.5 (78897.1-99669.7) | 147.28 (129.76-162.99) | 1.26^*^ (0.92 to 1.6) |
| **Tropical Latin America** | 115107.9 (110620.3-118750.1) | 120.69 (115.51-124.62) | 381975.9 (361667.4-398097.3) | 146.52 (138.52-152.83) | 0.66^*^ (0.39 to 0.93) |
| **Western Europe** | 1068715.0 (1021579.4-1109467.0) | 190.87 (183.00-197.87) | 1738600.4 (1598781.1-1842151.6) | 199.42 (185.68-210.22) | 0.13^*^ (0.03 to 0.22) |
| **Western Sub-Saharan Africa** | 23808.2 (20239.1-27490.0) | 25.84 (22.02-29.74) | 101338.9 (83266.4-119898.6) | 48.35 (40.20-56.31) | 2.05^*^ (1.95 to 2.14) |

*Abbreviations: ASR, age-standardized rate; SDI, sociodemographic index; GBD, Global Burden of Disease; AAPC, average annual percentage change; DALYs, disability-adjusted life years; UI, uncertainty interval; CI, confidence interval.*

# Table S5. Case numbers and ASRs of incidence of pancreatic cancer in 1990 and 2021 for both sexes in 204 countries and AAPC from 1990 to 2021.

| **Location** | **1990** | |  | **2021** | |  | **1990-2021** |
| --- | --- | --- | --- | --- | --- | --- | --- |
|  | **Number**  **(95 % UI)** | **ASR**  **(95 % UI)** |  | **Number**  **(95 % UI)** | **ASR**  **(95 % UI)** |  | **AAPC**  **(95% CI)** |
| **Afghanistan** | 130.7 (67.2-247.3) | 1.87 (0.98-3.45) |  | 258.8 (156.4-456.6) | 2.71 (1.74-4.64) |  | 1.18 (1.12 to 1.25) |
| **Albania** | 77.4 (60.7-95.1) | 3.89 (3.07-4.76) |  | 219.8 (153.3-298.1) | 4.98 (3.48-6.76) |  | 0.73 (0.36 to 1.1) |
| **Algeria** | 137.5 (110.9-169.2) | 1.27 (1.03-1.56) |  | 563.8 (438.8-725.0) | 1.73 (1.37-2.20) |  | 1.02 (0.85 to 1.19) |
| **American Samoa** | 0.7 (0.6-0.9) | 3.32 (2.72-3.98) |  | 2.3 (1.9-2.8) | 4.89 (4.06-5.90) |  | 1.32 (1.05 to 1.59) |
| **Andorra** | 7.6 (5.3-10.7) | 13.31 (9.47-18.58) |  | 16.1 (11.1-21.4) | 10.29 (7.05-13.61) |  | -0.96 (-1.32 to -0.6) |
| **Angola** | 82.6 (61.0-112.6) | 2.16 (1.63-2.86) |  | 299.6 (183.7-429.3) | 2.62 (1.59-3.77) |  | 0.63 (0.46 to 0.81) |
| **Antigua and Barbuda** | 2.3 (2.1-2.5) | 4.18 (3.85-4.50) |  | 5.6 (5.3-5.9) | 5.32 (5.05-5.64) |  | 0.83 (-0.39 to 2.06) |
| **Argentina** | 3137.1 (2923.5-3368.8) | 9.75 (9.09-10.46) |  | 5218.7 (4791.1-5591.0) | 9.24 (8.51-9.90) |  | -0.15 (-0.43 to 0.14) |
| **Armenia** | 191.2 (163.8-227.2) | 7.06 (6.06-8.41) |  | 430.2 (359.9-509.7) | 9.82 (8.23-11.63) |  | 1.14 (0.37 to 1.91) |
| **Australia** | 1522.8 (1439.7-1592.2) | 7.76 (7.34-8.12) |  | 4093.8 (3624.2-4405.1) | 8.76 (7.85-9.41) |  | 0.38 (-0.07 to 0.84) |
| **Austria** | 1162.4 (1095.2-1216.4) | 9.67 (9.13-10.09) |  | 1938.0 (1702.8-2088.2) | 10.37 (9.29-11.13) |  | 0.24 (-0.18 to 0.66) |
| **Azerbaijan** | 133.3 (99.9-185.7) | 2.64 (1.98-3.63) |  | 431.1 (307.1-619.1) | 4.21 (3.07-5.95) |  | 1.53 (1.23 to 1.84) |
| **Bahamas** | 5.9 (5.2-6.5) | 3.80 (3.41-4.22) |  | 17.1 (13.9-20.7) | 4.24 (3.49-5.15) |  | 0.44 (-0.02 to 0.89) |
| **Bahrain** | 9.8 (8.4-11.2) | 6.41 (5.46-7.36) |  | 50.3 (38.5-69.8) | 6.67 (5.25-8.92) |  | 0.14 (-0.14 to 0.41) |
| **Bangladesh** | 471.7 (275.2-645.7) | 1.01 (0.59-1.39) |  | 1543.6 (1045.0-2214.0) | 1.14 (0.78-1.61) |  | 0.48 (0.2 to 0.76) |
| **Barbados** | 19.5 (17.6-21.3) | 6.59 (5.97-7.19) |  | 36.9 (29.0-45.4) | 7.08 (5.57-8.72) |  | 0.24 (-0.09 to 0.56) |
| **Belarus** | 722.0 (666.5-778.5) | 5.52 (5.10-5.96) |  | 1173.0 (960.6-1432.0) | 7.30 (5.99-8.85) |  | 0.89 (0.43 to 1.35) |
| **Belgium** | 1339.5 (1242.0-1409.4) | 8.57 (7.97-9.01) |  | 2143.9 (1883.9-2333.9) | 8.98 (8.08-9.71) |  | 0.09 (-0.58 to 0.77) |
| **Belize** | 3.1 (2.7-3.9) | 3.33 (2.87-4.19) |  | 15.1 (13.4-17.2) | 5.11 (4.51-5.78) |  | 1.43 (0.86 to 2) |
| **Benin** | 27.9 (23.0-33.2) | 1.43 (1.19-1.71) |  | 129.5 (92.5-173.4) | 2.62 (1.90-3.44) |  | 1.97 (1.85 to 2.09) |
| **Bermuda** | 6.4 (5.0-7.5) | 10.59 (8.29-12.25) |  | 11.5 (9.7-14.1) | 8.16 (6.97-10.01) |  | -0.97 (-1.54 to -0.39) |
| **Bhutan** | 2.2 (1.0-3.6) | 0.93 (0.41-1.47) |  | 8.8 (4.9-12.5) | 1.48 (0.83-2.10) |  | 1.52 (1.37 to 1.67) |
| **Bolivia (Plurinational State of)** | 161.2 (105.8-207.5) | 5.21 (3.46-6.64) |  | 567.4 (376.2-800.8) | 6.43 (4.29-9.04) |  | 0.69 (0.52 to 0.85) |
| **Bosnia and Herzegovina** | 268.4 (228.4-307.3) | 6.61 (5.62-7.57) |  | 525.4 (423.8-649.3) | 8.30 (6.66-10.27) |  | 0.8 (0.59 to 1.01) |
| **Botswana** | 18.5 (13.2-24.1) | 3.44 (2.48-4.48) |  | 64.5 (45.3-88.7) | 4.65 (3.34-6.17) |  | 0.93 (0.32 to 1.53) |
| **Brazil** | 4135.9 (3947.7-4272.0) | 4.81 (4.54-4.98) |  | 14717.1 (13651.1-15441.2) | 5.88 (5.44-6.18) |  | 0.68 (0.38 to 0.98) |
| **Brunei Darussalam** | 5.1 (4.0-6.4) | 5.30 (4.15-6.61) |  | 18.2 (14.9-22.0) | 5.63 (4.60-6.85) |  | 0.13 (-0.07 to 0.33) |
| **Bulgaria** | 852.2 (777.6-937.2) | 7.00 (6.42-7.66) |  | 1461.5 (1232.1-1705.2) | 10.33 (8.68-12.09) |  | 1.29 (0.59 to 1.98) |
| **Burkina Faso** | 52.2 (39.3-67.0) | 1.24 (0.94-1.59) |  | 204.3 (145.2-279.9) | 2.30 (1.65-3.13) |  | 1.99 (1.7 to 2.28) |
| **Burundi** | 50.1 (38.6-63.1) | 2.17 (1.69-2.73) |  | 77.5 (51.6-117.1) | 1.64 (1.11-2.44) |  | -0.92 (-1.1 to -0.73) |
| **Cabo Verde** | 1.5 (1.3-1.8) | 0.65 (0.54-0.78) |  | 36.5 (26.8-46.0) | 8.55 (6.25-10.96) |  | 8.81 (8.07 to 9.56) |
| **Cambodia** | 106.1 (75.5-152.4) | 2.34 (1.66-3.32) |  | 396.2 (297.7-508.6) | 3.23 (2.48-4.06) |  | 1.06 (0.98 to 1.14) |
| **Cameroon** | 90.7 (71.8-113.3) | 2.12 (1.69-2.66) |  | 455.8 (315.4-633.0) | 3.75 (2.66-5.23) |  | 1.86 (1.76 to 1.96) |
| **Canada** | 2991.0 (2803.7-3146.7) | 9.16 (8.58-9.64) |  | 6816.5 (6012.7-7388.7) | 9.05 (8.06-9.77) |  | -0.08 (-0.3 to 0.14) |
| **Central African Republic** | 26.1 (18.0-33.1) | 2.30 (1.72-2.84) |  | 47.4 (34.1-66.3) | 2.14 (1.56-2.90) |  | -0.23 (-0.34 to -0.13) |
| **Chad** | 25.9 (19.4-33.4) | 0.93 (0.70-1.20) |  | 112.3 (85.0-143.4) | 2.00 (1.52-2.56) |  | 2.55 (2.48 to 2.62) |
| **Chile** | 546.0 (513.6-576.8) | 5.57 (5.24-5.90) |  | 1666.8 (1528.1-1804.0) | 6.42 (5.89-6.94) |  | 0.43 (-0.03 to 0.89) |
| **China** | 37817.7 (31791.4-44068.3) | 4.54 (3.84-5.29) |  | 118665.4 (94622.7-144663.1) | 5.64 (4.52-6.84) |  | 0.71 (0.51 to 0.92) |
| **Colombia** | 865.6 (821.2-903.1) | 5.05 (4.78-5.27) |  | 2373.9 (1984.1-2802.5) | 4.31 (3.61-5.09) |  | -0.56 (-1.17 to 0.07) |
| **Comoros** | 4.9 (3.7-6.5) | 2.53 (1.91-3.34) |  | 13.0 (9.2-18.0) | 2.73 (1.97-3.73) |  | 0.23 (0.06 to 0.39) |
| **Congo** | 36.9 (24.1-49.8) | 3.52 (2.39-4.68) |  | 95.0 (63.2-137.3) | 3.59 (2.37-5.14) |  | 0.1 (-0.12 to 0.32) |
| **Cook Islands** | 0.4 (0.4-0.5) | 3.50 (2.99-4.12) |  | 1.0 (0.8-1.2) | 3.76 (2.98-4.63) |  | 0.25 (0.11 to 0.39) |
| **Costa Rica** | 67.0 (62.1-72.0) | 3.91 (3.62-4.20) |  | 296.7 (262.4-334.8) | 5.41 (4.79-6.11) |  | 1.05 (0.35 to 1.75) |
| **Coted'Ivoire** | 50.0 (39.4-63.5) | 1.33 (1.08-1.63) |  | 182.2 (128.0-257.5) | 1.71 (1.24-2.33) |  | 0.82 (0.63 to 1.01) |
| **Croatia** | 519.2 (470.6-561.0) | 8.74 (7.90-9.46) |  | 885.2 (768.1-1017.7) | 9.55 (8.29-10.98) |  | 0.31 (-0.56 to 1.19) |
| **Cuba** | 584.5 (547.0-622.5) | 5.65 (5.30-6.01) |  | 1000.3 (868.8-1147.1) | 5.07 (4.40-5.82) |  | -0.4 (-0.94 to 0.15) |
| **Cyprus** | 43.7 (34.9-53.6) | 6.11 (4.85-7.54) |  | 138.9 (109.4-170.7) | 6.78 (5.37-8.25) |  | 0.31 (0.05 to 0.58) |
| **Czechia** | 1593.7 (1469.7-1733.6) | 11.46 (10.56-12.49) |  | 2580.0 (2195.4-2981.0) | 11.73 (9.97-13.47) |  | 0.09 (-0.01 to 0.19) |
| **Democratic People's Republic of Korea** | 552.5 (393.0-758.2) | 3.36 (2.44-4.56) |  | 1164.6 (746.7-1572.5) | 3.47 (2.22-4.67) |  | 0.11 (0.06 to 0.15) |
| **Democratic Republic of the Congo** | 305.7 (241.8-378.8) | 2.02 (1.61-2.50) |  | 693.4 (430.9-1011.3) | 1.95 (1.20-2.87) |  | -0.13 (-0.24 to -0.03) |
| **Denmark** | 545.4 (494.5-612.2) | 6.72 (6.15-7.48) |  | 1260.6 (1133.7-1364.4) | 10.18 (9.24-10.97) |  | 1.41 (0.72 to 2.12) |
| **Djibouti** | 2.9 (1.8-4.1) | 2.22 (1.38-3.06) |  | 17.0 (10.3-25.5) | 2.78 (1.75-4.05) |  | 0.73 (0.65 to 0.8) |
| **Dominica** | 3.4 (2.7-4.4) | 5.69 (4.61-7.32) |  | 6.4 (5.1-8.2) | 7.75 (6.21-9.84) |  | 1.01 (0.9 to 1.12) |
| **Dominican Republic** | 110.5 (89.5-137.3) | 3.08 (2.50-3.81) |  | 527.8 (413.7-681.3) | 5.30 (4.14-6.83) |  | 1.84 (1.39 to 2.29) |
| **Ecuador** | 174.4 (161.6-187.7) | 3.44 (3.17-3.70) |  | 712.7 (554.3-885.5) | 4.41 (3.45-5.47) |  | 0.72 (0.05 to 1.4) |
| **Egypt** | 556.4 (489.0-638.8) | 2.10 (1.85-2.40) |  | 3268.8 (2693.4-4000.5) | 5.39 (4.48-6.55) |  | 3.1 (2.89 to 3.31) |
| **El Salvador** | 69.8 (63.0-77.2) | 2.36 (2.12-2.61) |  | 271.5 (220.0-335.6) | 4.40 (3.56-5.43) |  | 2.2 (1.81 to 2.6) |
| **Equatorial Guinea** | 4.5 (3.2-5.8) | 2.32 (1.71-2.95) |  | 21.4 (12.4-31.2) | 4.37 (2.54-6.23) |  | 2.08 (1.75 to 2.4) |
| **Eritrea** | 23.5 (18.1-29.7) | 2.09 (1.64-2.58) |  | 62.2 (38.2-88.0) | 2.33 (1.46-3.27) |  | 0.34 (0.23 to 0.44) |
| **Estonia** | 176.4 (160.5-193.7) | 8.59 (7.82-9.44) |  | 288.1 (242.8-327.6) | 10.47 (8.92-11.87) |  | 0.61 (-0.27 to 1.49) |
| **Eswatini** | 12.5 (8.5-18.2) | 4.52 (3.07-6.53) |  | 39.7 (24.2-59.6) | 7.16 (4.39-10.53) |  | 1.55 (1.28 to 1.81) |
| **Ethiopia** | 199.7 (93.6-311.0) | 1.02 (0.47-1.63) |  | 450.7 (269.7-706.8) | 1.07 (0.64-1.68) |  | 0.19 (0.11 to 0.27) |
| **Fiji** | 7.9 (6.4-9.5) | 2.26 (1.83-2.73) |  | 23.1 (17.2-30.6) | 3.10 (2.36-4.05) |  | 1 (0.9 to 1.1) |
| **Finland** | 794.5 (740.7-835.0) | 10.95 (10.21-11.51) |  | 1626.3 (1419.8-1785.0) | 12.15 (10.80-13.25) |  | 0.34 (0 to 0.69) |
| **France** | 6025.0 (5625.3-6552.3) | 7.29 (6.83-7.88) |  | 15011.3 (13144.1-16820.4) | 10.51 (9.42-11.64) |  | 1.2 (1.07 to 1.34) |
| **Gabon** | 20.6 (13.8-31.2) | 3.66 (2.44-5.55) |  | 51.7 (36.4-69.3) | 5.10 (3.64-6.71) |  | 1.09 (0.89 to 1.29) |
| **Gambia** | 4.4 (3.4-5.6) | 1.29 (0.99-1.60) |  | 18.7 (13.3-24.5) | 1.96 (1.40-2.57) |  | 1.42 (0.94 to 1.9) |
| **Georgia** | 125.6 (101.9-154.3) | 1.95 (1.58-2.39) |  | 387.8 (336.9-446.0) | 6.57 (5.72-7.53) |  | 4.32 (2.7 to 5.97) |
| **Germany** | 11097.6 (10254.7-11736.7) | 8.61 (8.01-9.12) |  | 21956.1 (19693.3-23713.8) | 11.09 (10.12-11.90) |  | 0.8 (0.52 to 1.07) |
| **Ghana** | 82.9 (56.7-118.1) | 1.37 (0.95-1.97) |  | 621.5 (446.6-873.2) | 3.89 (2.81-5.53) |  | 3.42 (3.31 to 3.54) |
| **Greece** | 1389.6 (1307.8-1453.8) | 9.04 (8.51-9.44) |  | 2436.5 (2200.9-2585.6) | 10.00 (9.17-10.53) |  | 0.31 (-0.01 to 0.63) |
| **Greenland** | 6.2 (5.3-7.2) | 19.19 (16.17-22.12) |  | 10.5 (8.6-12.8) | 15.21 (12.40-18.51) |  | -0.72 (-1.04 to -0.39) |
| **Grenada** | 4.7 (4.1-5.3) | 6.46 (5.67-7.31) |  | 10.2 (8.9-11.4) | 9.10 (7.99-10.14) |  | 1.16 (0.66 to 1.66) |
| **Guam** | 2.5 (2.2-2.9) | 3.62 (3.12-4.18) |  | 8.1 (6.9-9.4) | 3.90 (3.31-4.49) |  | 0.16 (-1.05 to 1.38) |
| **Guatemala** | 61.4 (58.7-64.2) | 1.87 (1.79-1.96) |  | 344.5 (297.2-399.2) | 3.16 (2.74-3.65) |  | 1.45 (0.4 to 2.51) |
| **Guinea** | 29.0 (22.7-36.9) | 0.89 (0.70-1.13) |  | 79.4 (59.5-107.4) | 1.44 (1.08-1.93) |  | 1.56 (1.45 to 1.66) |
| **Guinea-Bissau** | 7.2 (4.7-9.7) | 1.84 (1.23-2.45) |  | 21.8 (15.2-29.1) | 3.09 (2.18-4.07) |  | 1.69 (1.61 to 1.77) |
| **Guyana** | 12.8 (11.4-14.4) | 3.43 (3.05-3.86) |  | 31.4 (24.3-40.2) | 4.88 (3.82-6.18) |  | 1.29 (0.67 to 1.92) |
| **Haiti** | 119.1 (79.0-154.3) | 3.80 (2.62-4.88) |  | 260.5 (178.5-360.1) | 3.74 (2.58-5.14) |  | -0.01 (-0.07 to 0.06) |
| **Honduras** | 52.4 (43.9-62.0) | 2.58 (2.16-3.05) |  | 300.1 (218.3-419.6) | 4.84 (3.47-6.78) |  | 2.1 (1.67 to 2.52) |
| **Hungary** | 1415.8 (1292.4-1541.3) | 9.65 (8.82-10.48) |  | 2070.6 (1790.0-2356.7) | 10.61 (9.19-12.06) |  | 0.35 (0.11 to 0.58) |
| **Iceland** | 25.7 (23.7-27.3) | 8.89 (8.20-9.44) |  | 55.3 (47.7-61.6) | 9.29 (8.14-10.32) |  | 0.11 (-0.71 to 0.93) |
| **India** | 4656.7 (3702.9-5668.8) | 1.01 (0.80-1.23) |  | 17086.9 (14891.6-19179.7) | 1.45 (1.26-1.62) |  | 1.18 (0.98 to 1.39) |
| **Indonesia** | 2128.6 (1644.1-2608.1) | 2.15 (1.64-2.65) |  | 8701.4 (6253.0-11152.9) | 3.68 (2.63-4.71) |  | 1.75 (1.7 to 1.81) |
| **Iran (Islamic Republic of)** | 482.2 (395.4-563.9) | 1.98 (1.62-2.30) |  | 2671.1 (2353.1-2956.3) | 3.57 (3.14-3.96) |  | 1.9 (1.62 to 2.19) |
| **Iraq** | 231.5 (159.3-309.5) | 2.93 (2.01-3.92) |  | 1095.0 (803.5-1408.5) | 4.75 (3.52-6.05) |  | 1.61 (1.37 to 1.85) |
| **Ireland** | 388.5 (365.7-406.9) | 9.42 (8.88-9.87) |  | 615.1 (549.2-679.4) | 7.61 (6.83-8.40) |  | -0.66 (-1.12 to -0.2) |
| **Israel** | 436.1 (408.7-461.4) | 9.00 (8.39-9.53) |  | 1133.3 (996.9-1232.3) | 8.97 (7.94-9.71) |  | 0.02 (-0.48 to 0.52) |
| **Italy** | 7985.7 (7502.3-8288.1) | 8.90 (8.37-9.23) |  | 14413.5 (12499.0-15592.8) | 9.48 (8.44-10.16) |  | 0.16 (-0.07 to 0.4) |
| **Jamaica** | 60.4 (54.6-68.9) | 3.36 (3.04-3.84) |  | 128.4 (100.4-165.6) | 4.15 (3.25-5.35) |  | 0.84 (0.03 to 1.65) |
| **Japan** | 16074.7 (15142.0-16620.0) | 9.52 (8.92-9.86) |  | 46502.1 (39057.0-50709.3) | 11.55 (10.13-12.34) |  | 0.67 (0.56 to 0.77) |
| **Jordan** | 31.0 (25.5-37.4) | 2.40 (1.96-2.89) |  | 233.3 (171.5-314.1) | 3.28 (2.43-4.37) |  | 1.05 (0.55 to 1.55) |
| **Kazakhstan** | 623.6 (502.2-817.4) | 4.90 (3.96-6.37) |  | 981.4 (831.1-1143.3) | 5.38 (4.56-6.26) |  | 0.34 (-0.16 to 0.84) |
| **Kenya** | 119.2 (76.5-168.2) | 1.48 (0.96-2.09) |  | 641.0 (505.2-837.4) | 2.89 (2.26-3.69) |  | 2.16 (1.97 to 2.34) |
| **Kiribati** | 0.3 (0.3-0.4) | 0.93 (0.73-1.12) |  | 0.8 (0.6-1.1) | 1.18 (0.85-1.58) |  | 0.8 (0.74 to 0.86) |
| **Kuwait** | 17.6 (16.0-19.0) | 3.09 (2.78-3.36) |  | 121.8 (100.8-146.8) | 4.39 (3.58-5.31) |  | 1.13 (-0.95 to 3.25) |
| **Kyrgyzstan** | 111.9 (94.1-130.6) | 3.76 (3.16-4.38) |  | 239.7 (195.0-294.3) | 4.93 (4.02-6.04) |  | 0.95 (-0.47 to 2.38) |
| **Lao People's Democratic Republic** | 48.7 (28.7-76.7) | 2.32 (1.39-3.61) |  | 131.4 (93.8-176.8) | 2.87 (2.08-3.80) |  | 0.69 (0.62 to 0.76) |
| **Latvia** | 301.8 (273.9-328.7) | 8.35 (7.59-9.10) |  | 420.5 (360.0-484.9) | 10.67 (9.12-12.43) |  | 0.81 (0.32 to 1.3) |
| **Lebanon** | 72.7 (49.5-94.5) | 3.47 (2.40-4.45) |  | 264.3 (192.7-357.7) | 4.33 (3.16-5.85) |  | 0.75 (0.45 to 1.05) |
| **Lesotho** | 20.4 (15.4-27.4) | 2.48 (1.87-3.34) |  | 60.1 (38.9-86.5) | 5.68 (3.70-8.10) |  | 2.79 (2.5 to 3.09) |
| **Liberia** | 17.5 (12.6-23.0) | 1.55 (1.14-2.04) |  | 51.5 (28.7-78.2) | 2.53 (1.43-3.82) |  | 1.56 (1.28 to 1.84) |
| **Libya** | 94.3 (67.6-124.0) | 5.16 (3.74-6.78) |  | 387.8 (271.8-512.8) | 7.59 (5.36-10.01) |  | 1.24 (0.94 to 1.53) |
| **Lithuania** | 372.8 (339.8-404.1) | 8.22 (7.49-8.88) |  | 562.1 (488.9-631.8) | 9.90 (8.60-11.12) |  | 0.71 (-0.35 to 1.79) |
| **Luxembourg** | 50.2 (47.3-52.8) | 9.17 (8.64-9.63) |  | 93.3 (83.1-102.7) | 8.58 (7.67-9.43) |  | -0.24 (-0.73 to 0.25) |
| **Madagascar** | 90.8 (66.7-113.6) | 1.81 (1.33-2.27) |  | 181.8 (123.4-258.0) | 1.65 (1.13-2.31) |  | -0.33 (-0.54 to -0.12) |
| **Malawi** | 28.2 (21.8-35.1) | 0.75 (0.59-0.94) |  | 71.8 (51.1-99.8) | 1.00 (0.72-1.37) |  | 0.97 (0.81 to 1.12) |
| **Malaysia** | 154.9 (129.0-180.1) | 1.71 (1.41-2.01) |  | 728.1 (622.7-844.6) | 2.63 (2.24-3.05) |  | 1.48 (1.21 to 1.74) |
| **Maldives** | 1.7 (1.1-2.5) | 1.99 (1.32-2.81) |  | 5.7 (4.5-7.0) | 1.79 (1.41-2.18) |  | -0.39 (-0.6 to -0.19) |
| **Mali** | 72.9 (59.4-86.5) | 1.89 (1.54-2.25) |  | 206.6 (153.5-271.3) | 2.42 (1.82-3.13) |  | 0.85 (0.71 to 0.99) |
| **Malta** | 37.4 (34.6-40.1) | 8.75 (8.08-9.39) |  | 90.7 (79.8-101.9) | 9.24 (8.22-10.35) |  | 0.16 (-0.11 to 0.43) |
| **Marshall Islands** | 0.4 (0.3-0.4) | 2.34 (1.92-2.82) |  | 1.2 (0.9-1.7) | 3.51 (2.56-4.75) |  | 1.33 (1.24 to 1.43) |
| **Mauritania** | 18.8 (14.5-23.1) | 1.92 (1.48-2.35) |  | 73.3 (53.0-96.7) | 3.53 (2.55-4.66) |  | 2 (1.79 to 2.2) |
| **Mauritius** | 32.0 (30.2-33.7) | 4.46 (4.22-4.70) |  | 91.5 (84.8-96.6) | 4.97 (4.62-5.23) |  | 0.36 (-0.41 to 1.15) |
| **Mexico** | 2200.0 (2141.3-2249.9) | 5.39 (5.21-5.53) |  | 6026.9 (5314.3-6738.5) | 4.80 (4.23-5.36) |  | -0.41 (-0.76 to -0.06) |
| **Micronesia (Federated States of)** | 1.4 (1.1-1.7) | 2.85 (2.29-3.69) |  | 2.9 (2.1-3.9) | 4.06 (2.88-5.35) |  | 1.16 (1.12 to 1.19) |
| **Monaco** | 8.0 (5.6-10.8) | 11.26 (8.01-15.24) |  | 13.3 (8.8-19.3) | 13.27 (8.78-19.30) |  | 0.53 (0.47 to 0.59) |
| **Mongolia** | 14.9 (11.2-19.5) | 1.41 (1.07-1.86) |  | 172.5 (128.2-228.2) | 7.40 (5.46-9.86) |  | 5.46 (4.61 to 6.32) |
| **Montenegro** | 46.1 (37.6-56.5) | 7.37 (6.00-9.01) |  | 97.7 (76.6-120.4) | 10.02 (7.92-12.26) |  | 1.05 (0.69 to 1.41) |
| **Morocco** | 141.0 (114.6-172.0) | 1.01 (0.82-1.23) |  | 590.5 (433.2-727.7) | 1.74 (1.29-2.12) |  | 1.78 (1.69 to 1.87) |
| **Mozambique** | 27.6 (23.0-33.7) | 0.53 (0.44-0.63) |  | 77.8 (58.2-100.6) | 0.81 (0.61-1.04) |  | 1.41 (1.27 to 1.56) |
| **Myanmar** | 530.7 (351.0-804.9) | 2.27 (1.52-3.41) |  | 1402.7 (1033.2-1839.2) | 2.91 (2.19-3.79) |  | 0.81 (0.76 to 0.85) |
| **Namibia** | 6.7 (5.3-8.4) | 1.05 (0.83-1.29) |  | 20.2 (14.3-26.4) | 1.47 (1.07-1.88) |  | 1.13 (0.96 to 1.3) |
| **Nauru** | 0.2 (0.1-0.3) | 4.36 (2.89-6.66) |  | 0.3 (0.2-0.4) | 5.43 (3.37-7.45) |  | 0.71 (0.57 to 0.85) |
| **Nepal** | 76.4 (39.9-113.7) | 0.84 (0.43-1.23) |  | 287.9 (171.6-430.3) | 1.27 (0.76-1.88) |  | 1.36 (1.24 to 1.47) |
| **Netherlands** | 1628.2 (1528.7-1706.6) | 8.09 (7.61-8.47) |  | 2847.1 (2541.0-3069.2) | 7.91 (7.10-8.48) |  | -0.1 (-0.41 to 0.22) |
| **New Zealand** | 292.6 (271.6-310.0) | 7.39 (6.87-7.84) |  | 680.3 (607.0-734.3) | 7.89 (7.09-8.49) |  | 0.18 (-0.35 to 0.71) |
| **Nicaragua** | 27.7 (24.1-31.8) | 1.84 (1.59-2.13) |  | 140.9 (111.3-174.3) | 2.93 (2.32-3.63) |  | 1.58 (1.23 to 1.93) |
| **Niger** | 27.6 (19.4-35.6) | 1.04 (0.74-1.33) |  | 126.4 (79.7-181.3) | 1.62 (1.07-2.30) |  | 1.48 (1.34 to 1.62) |
| **Nigeria** | 254.7 (173.8-322.6) | 0.61 (0.42-0.76) |  | 872.4 (656.8-1140.2) | 1.03 (0.79-1.31) |  | 1.71 (1.64 to 1.79) |
| **Niue** | 0.1 (0.0-0.1) | 2.92 (2.17-3.70) |  | 0.1 (0.1-0.1) | 4.74 (3.62-6.11) |  | 1.57 (1.51 to 1.63) |
| **North Macedonia** | 118.5 (98.3-141.5) | 6.41 (5.32-7.68) |  | 275.4 (210.4-357.4) | 8.46 (6.52-10.77) |  | 0.92 (0.71 to 1.14) |
| **Northern Mariana Islands** | 0.6 (0.5-0.7) | 3.53 (2.86-4.40) |  | 3.5 (2.9-4.1) | 7.17 (6.01-8.43) |  | 2.22 (1.67 to 2.78) |
| **Norway** | 626.4 (585.7-650.2) | 8.96 (8.45-9.29) |  | 1009.5 (903.6-1073.1) | 9.71 (8.79-10.29) |  | 0.28 (0.09 to 0.48) |
| **Oman** | 8.3 (5.9-11.6) | 1.27 (0.90-1.76) |  | 35.7 (25.7-46.6) | 1.92 (1.42-2.41) |  | 1.37 (0.91 to 1.84) |
| **Pakistan** | 500.9 (421.1-591.4) | 0.91 (0.76-1.07) |  | 1650.3 (1281.3-2203.0) | 1.40 (1.09-1.89) |  | 1.41 (1.3 to 1.52) |
| **Palau** | 0.7 (0.6-0.9) | 8.27 (6.43-10.42) |  | 1.8 (1.4-2.2) | 9.44 (7.57-11.53) |  | 0.44 (0.24 to 0.64) |
| **Palestine** | 36.9 (25.9-50.7) | 4.46 (3.16-6.06) |  | 124.3 (102.5-146.7) | 5.24 (4.33-6.19) |  | 0.5 (0.32 to 0.68) |
| **Panama** | 30.9 (28.9-32.9) | 2.11 (1.97-2.25) |  | 158.8 (125.5-189.2) | 3.60 (2.85-4.29) |  | 1.77 (0.87 to 2.68) |
| **Papua New Guinea** | 24.1 (16.3-35.9) | 1.39 (0.96-2.06) |  | 87.0 (60.8-127.4) | 1.79 (1.24-2.61) |  | 0.83 (0.68 to 0.98) |
| **Paraguay** | 48.5 (40.9-57.9) | 2.24 (1.89-2.68) |  | 298.9 (227.6-390.7) | 5.26 (4.00-6.85) |  | 2.91 (2.64 to 3.18) |
| **Peru** | 532.4 (433.5-662.8) | 4.57 (3.76-5.71) |  | 1729.8 (1269.4-2287.5) | 5.22 (3.83-6.90) |  | 0.52 (-0.31 to 1.36) |
| **Philippines** | 663.1 (581.3-778.1) | 2.23 (1.94-2.65) |  | 2732.8 (2270.9-3257.8) | 3.31 (2.78-3.93) |  | 1.35 (1.19 to 1.52) |
| **Poland** | 3798.3 (3640.2-3931.2) | 8.69 (8.33-9.00) |  | 6141.0 (5583.6-6661.1) | 8.47 (7.72-9.19) |  | -0.2 (-0.28 to -0.12) |
| **Portugal** | 918.7 (865.7-967.7) | 6.69 (6.31-7.06) |  | 1725.2 (1533.8-1865.7) | 6.93 (6.28-7.45) |  | 0.09 (-0.12 to 0.29) |
| **Puerto Rico** | 151.5 (141.8-161.2) | 4.16 (3.90-4.43) |  | 433.2 (355.6-509.3) | 6.10 (5.02-7.20) |  | 1.29 (0.53 to 2.06) |
| **Qatar** | 5.9 (4.8-7.3) | 6.39 (5.28-7.71) |  | 50.2 (36.3-72.0) | 6.40 (4.70-9.00) |  | -0.14 (-0.7 to 0.41) |
| **Republic of Korea** | 2371.0 (2015.8-2728.2) | 8.22 (6.98-9.52) |  | 7784.1 (6114.3-9429.2) | 8.23 (6.45-9.99) |  | -0.05 (-0.3 to 0.21) |
| **Republic of Moldova** | 323.6 (298.8-350.9) | 7.36 (6.79-8.00) |  | 470.2 (417.8-527.2) | 7.85 (6.97-8.80) |  | 0.09 (-0.39 to 0.56) |
| **Romania** | 1696.0 (1569.7-1830.7) | 6.01 (5.55-6.49) |  | 3411.6 (2961.2-3868.2) | 9.35 (8.12-10.60) |  | 1.45 (1.08 to 1.83) |
| **Russian Federation** | 14421.8 (14055.0-14753.2) | 7.93 (7.72-8.13) |  | 21082.7 (19374.2-22839.5) | 8.78 (8.07-9.50) |  | 0.44 (-0.05 to 0.93) |
| **Rwanda** | 81.3 (63.3-99.0) | 2.89 (2.33-3.50) |  | 149.8 (101.5-218.8) | 2.49 (1.72-3.59) |  | -0.47 (-0.58 to -0.37) |
| **Saint Kitts and Nevis** | 2.4 (2.0-2.7) | 6.29 (5.20-7.17) |  | 4.5 (3.7-5.3) | 6.93 (5.81-7.99) |  | 0.32 (-0.14 to 0.77) |
| **Saint Lucia** | 4.6 (4.0-5.1) | 5.51 (4.87-6.08) |  | 14.5 (12.0-17.5) | 6.08 (5.04-7.32) |  | 0.19 (-0.33 to 0.71) |
| **Saint Vincent and the Grenadines** | 3.7 (3.3-4.0) | 5.23 (4.77-5.72) |  | 8.0 (7.0-9.1) | 5.63 (4.99-6.40) |  | 0.21 (-0.26 to 0.68) |
| **Samoa** | 2.6 (2.1-3.1) | 3.17 (2.63-3.75) |  | 5.0 (3.9-6.3) | 3.52 (2.80-4.43) |  | 0.34 (0.25 to 0.42) |
| **San Marino** | 3.1 (2.6-3.6) | 8.59 (7.21-10.12) |  | 4.3 (2.8-6.0) | 5.59 (3.61-8.02) |  | -1.54 (-1.9 to -1.19) |
| **Sao Tome and Principe** | 0.4 (0.3-0.4) | 0.57 (0.47-0.68) |  | 1.0 (0.8-1.4) | 0.95 (0.73-1.29) |  | 1.62 (1.37 to 1.86) |
| **Saudi Arabia** | 90.9 (69.1-116.4) | 1.61 (1.22-2.04) |  | 593.2 (468.3-748.3) | 3.09 (2.51-3.83) |  | 2.12 (2.06 to 2.19) |
| **Senegal** | 47.6 (38.9-57.9) | 1.51 (1.24-1.83) |  | 220.2 (166.1-287.6) | 2.94 (2.24-3.77) |  | 2.18 (1.83 to 2.53) |
| **Serbia** | 817.2 (649.5-1036.0) | 7.65 (6.06-9.63) |  | 1418.3 (1117.9-1737.9) | 8.49 (6.67-10.46) |  | 0.4 (-0.02 to 0.82) |
| **Seychelles** | 2.7 (2.3-3.1) | 4.86 (4.14-5.54) |  | 6.0 (5.0-7.2) | 5.22 (4.35-6.20) |  | 0.09 (-0.23 to 0.41) |
| **Sierra Leone** | 24.1 (18.7-29.8) | 1.20 (0.94-1.47) |  | 77.7 (55.7-102.8) | 2.12 (1.54-2.76) |  | 1.86 (1.74 to 1.99) |
| **Singapore** | 111.1 (104.6-117.3) | 5.18 (4.89-5.47) |  | 469.0 (421.5-505.8) | 5.54 (4.94-5.98) |  | 0.21 (-0.96 to 1.4) |
| **Slovakia** | 560.7 (468.4-673.7) | 9.32 (7.76-11.24) |  | 929.4 (737.7-1177.2) | 9.69 (7.69-12.29) |  | 0.1 (-0.26 to 0.46) |
| **Slovenia** | 200.1 (186.2-214.0) | 8.10 (7.54-8.65) |  | 385.9 (324.2-445.2) | 8.45 (7.15-9.72) |  | 0.03 (-0.61 to 0.67) |
| **Solomon Islands** | 2.4 (1.5-3.3) | 1.82 (1.19-2.49) |  | 9.1 (6.6-12.3) | 2.66 (1.96-3.58) |  | 1.2 (0.98 to 1.43) |
| **Somalia** | 39.8 (23.9-63.7) | 1.69 (1.01-2.69) |  | 82.5 (42.6-135.9) | 1.36 (0.70-2.25) |  | -0.69 (-0.76 to -0.62) |
| **South Africa** | 766.7 (641.5-983.9) | 3.77 (3.13-4.87) |  | 2555.3 (2242.9-2858.2) | 5.67 (4.98-6.33) |  | 1.3 (0.9 to 1.7) |
| **South Sudan** | 57.8 (36.5-89.3) | 2.27 (1.44-3.53) |  | 90.6 (54.1-130.8) | 2.39 (1.45-3.39) |  | 0.16 (0.06 to 0.27) |
| **Spain** | 3838.4 (3592.1-4038.8) | 7.02 (6.58-7.39) |  | 7830.1 (6815.1-8548.7) | 7.89 (6.96-8.54) |  | 0.36 (0.21 to 0.51) |
| **Sri Lanka** | 139.9 (121.5-160.4) | 1.33 (1.16-1.53) |  | 386.8 (251.0-536.0) | 1.43 (0.93-1.97) |  | 0.16 (-0.42 to 0.75) |
| **Sudan** | 120.4 (79.0-184.2) | 1.32 (0.87-1.98) |  | 462.7 (297.9-707.4) | 2.42 (1.57-3.67) |  | 2 (1.92 to 2.08) |
| **Suriname** | 11.9 (10.0-13.8) | 4.73 (3.99-5.48) |  | 36.1 (27.4-46.4) | 5.69 (4.34-7.35) |  | 0.66 (0.25 to 1.07) |
| **Sweden** | 1434.3 (1330.1-1517.8) | 9.36 (8.73-9.87) |  | 1953.2 (1674.9-2190.0) | 8.59 (7.46-9.60) |  | -0.31 (-0.81 to 0.2) |
| **Switzerland** | 671.8 (606.9-744.7) | 6.37 (5.80-7.02) |  | 1573.7 (1368.9-1755.3) | 8.30 (7.36-9.19) |  | 0.77 (0.2 to 1.34) |
| **Syrian Arab Republic** | 119.4 (88.3-152.1) | 2.33 (1.73-2.96) |  | 469.9 (334.7-640.0) | 3.66 (2.63-4.91) |  | 1.4 (1.12 to 1.68) |
| **Taiwan (Province of China)** | 659.8 (628.1-688.5) | 4.10 (3.90-4.31) |  | 3000.8 (2707.6-3222.5) | 7.08 (6.41-7.62) |  | 1.73 (1.24 to 2.22) |
| **Tajikistan** | 44.9 (30.0-63.0) | 1.66 (1.09-2.37) |  | 82.7 (57.1-118.1) | 1.47 (1.02-2.08) |  | -0.45 (-0.84 to -0.05) |
| **Thailand** | 1223.3 (1029.8-1449.6) | 3.51 (2.97-4.14) |  | 4975.3 (3859.7-6445.0) | 4.59 (3.57-5.91) |  | 0.89 (0.7 to 1.08) |
| **Timor-Leste** | 4.2 (2.5-6.1) | 1.53 (0.93-2.19) |  | 17.4 (13.5-22.2) | 2.04 (1.58-2.57) |  | 0.96 (0.76 to 1.16) |
| **Togo** | 15.9 (12.1-20.1) | 1.34 (1.03-1.68) |  | 99.2 (67.8-134.3) | 2.70 (1.86-3.68) |  | 2.3 (2.23 to 2.38) |
| **Tokelau** | 0.0 (0.0-0.0) | 2.41 (1.71-3.31) |  | 0.1 (0.0-0.1) | 3.50 (2.46-4.74) |  | 1.23 (1.18 to 1.28) |
| **Tonga** | 2.1 (1.7-2.7) | 3.97 (3.11-5.04) |  | 4.2 (3.2-5.5) | 5.33 (4.04-6.87) |  | 0.94 (0.65 to 1.24) |
| **Trinidad and Tobago** | 40.9 (38.3-43.8) | 4.99 (4.64-5.35) |  | 98.9 (75.0-125.2) | 5.10 (3.88-6.46) |  | 0.22 (-0.18 to 0.62) |
| **Tunisia** | 94.0 (74.7-114.2) | 1.95 (1.57-2.36) |  | 367.0 (265.8-502.7) | 2.80 (2.02-3.80) |  | 1.15 (1.02 to 1.28) |
| **Turkey** | 2226.6 (1729.7-2852.0) | 6.54 (5.09-8.25) |  | 7418.2 (5761.6-8945.8) | 7.98 (6.22-9.61) |  | 0.64 (0.39 to 0.9) |
| **Turkmenistan** | 3.2 (2.8-3.7) | 0.17 (0.15-0.19) |  | 113.1 (85.3-151.6) | 2.73 (2.08-3.63) |  | 9.57 (7.05 to 12.14) |
| **Tuvalu** | 0.1 (0.1-0.2) | 2.21 (1.86-2.67) |  | 0.3 (0.3-0.4) | 3.38 (2.63-4.29) |  | 1.38 (1.31 to 1.45) |
| **Uganda** | 153.6 (120.5-195.0) | 2.46 (1.95-3.08) |  | 531.9 (373.7-728.7) | 3.73 (2.69-5.06) |  | 1.36 (1.19 to 1.53) |
| **Ukraine** | 3722.5 (2984.6-4731.8) | 5.17 (4.15-6.58) |  | 5097.3 (3803.7-6478.6) | 6.73 (5.01-8.58) |  | 0.82 (0.07 to 1.57) |
| **United Arab Emirates** | 35.5 (22.8-59.7) | 8.41 (5.47-14.18) |  | 319.8 (248.2-398.9) | 10.65 (8.52-13.15) |  | 0.71 (-1.31 to 2.77) |
| **United Kingdom** | 7916.9 (7547.2-8108.3) | 8.62 (8.23-8.82) |  | 11754.7 (10658.9-12283.0) | 8.67 (7.97-9.02) |  | 0.04 (-0.1 to 0.19) |
| **United Republic of Tanzania** | 240.8 (183.0-321.0) | 2.25 (1.74-2.97) |  | 605.9 (428.1-888.9) | 2.44 (1.78-3.52) |  | 0.27 (0.16 to 0.39) |
| **United States of America** | 30071.7 (28100.1-31133.4) | 9.33 (8.77-9.65) |  | 61340.2 (56242.6-64259.2) | 10.33 (9.53-10.80) |  | 0.31 (0.14 to 0.48) |
| **United States Virgin Islands** | 3.3 (2.7-3.8) | 3.98 (3.31-4.72) |  | 8.1 (6.0-10.6) | 4.59 (3.45-6.03) |  | 0.37 (-0.24 to 0.99) |
| **Uruguay** | 392.2 (363.0-419.1) | 9.95 (9.22-10.63) |  | 704.9 (636.7-775.1) | 12.55 (11.38-13.72) |  | 0.81 (0.73 to 0.89) |
| **Uzbekistan** | 168.2 (131.2-223.1) | 1.45 (1.12-1.95) |  | 629.6 (494.6-759.2) | 2.35 (1.87-2.86) |  | 1.64 (1.17 to 2.11) |
| **Vanuatu** | 1.1 (0.8-1.4) | 1.84 (1.44-2.32) |  | 4.5 (3.6-5.5) | 2.62 (2.13-3.21) |  | 1.14 (0.98 to 1.3) |
| **Venezuela (Bolivarian Republic of)** | 131.5 (124.8-137.8) | 1.38 (1.30-1.45) |  | 1370.3 (986.8-1784.6) | 4.60 (3.34-5.97) |  | 3.85 (3.11 to 4.61) |
| **Viet Nam** | 539.9 (431.1-691.6) | 1.36 (1.09-1.74) |  | 2096.2 (1558.2-2573.4) | 2.13 (1.60-2.59) |  | 1.48 (1.37 to 1.59) |
| **Yemen** | 59.7 (34.5-92.3) | 1.24 (0.71-1.86) |  | 247.7 (158.3-343.6) | 1.81 (1.16-2.51) |  | 1.24 (1.1 to 1.38) |
| **Zambia** | 74.1 (61.4-89.5) | 2.65 (2.22-3.18) |  | 212.6 (149.9-287.2) | 3.12 (2.24-4.14) |  | 0.54 (0.36 to 0.72) |
| **Zimbabwe** | 177.9 (143.2-214.0) | 4.66 (3.75-5.58) |  | 491.4 (367.5-628.1) | 7.25 (5.54-9.16) |  | 1.52 (1.13 to 1.92) |

*Abbreviations: ASR, age-standardized rate; AAPC, average annual percentage change; UI, uncertainty interval; CI, confidence interval.*

# Table S6. Case numbers and ASRs of prevalence of pancreatic cancer in 1990 and 2021 for both sexes in 204 countries and AAPC from 1990 to 2021.

| **Location** | **1990** | |  | **2021** | |  | **1990-2021** |
| --- | --- | --- | --- | --- | --- | --- | --- |
|  | **Number**  **(95 % UI)** | **ASR**  **(95 % UI)** |  | **Number**  **(95 % UI)** | **ASR**  **(95 % UI)** |  | **AAPC**  **(95% CI)** |
| **Afghanistan** | 102.1 (51.0-197.2) | 1.42 (0.74-2.69) |  | 219.7 (127.1-390.4) | 2.06 (1.27-3.59) |  | 1.2 (1.1 to 1.3) |
| **Albania** | 61.1 (47.8-75.8) | 2.90 (2.29-3.56) |  | 163.5 (114.3-222.2) | 3.77 (2.63-5.14) |  | 0.78 (0.43 to 1.14) |
| **Algeria** | 108.1 (87.4-133.2) | 0.91 (0.73-1.11) |  | 443.3 (344.4-569.3) | 1.27 (0.99-1.62) |  | 1.07 (0.91 to 1.23) |
| **American Samoa** | 0.6 (0.5-0.7) | 2.51 (2.06-3.01) |  | 1.9 (1.5-2.3) | 3.76 (3.11-4.57) |  | 1.37 (1.08 to 1.66) |
| **Andorra** | 6.1 (4.3-8.6) | 10.54 (7.39-14.74) |  | 12.9 (8.8-17.2) | 8.48 (5.74-11.32) |  | -0.9 (-1.5 to -0.29) |
| **Angola** | 67.8 (49.7-92.4) | 1.61 (1.21-2.17) |  | 249.8 (153.3-360.9) | 1.97 (1.20-2.84) |  | 0.6 (0.52 to 0.68) |
| **Antigua and Barbuda** | 1.7 (1.5-1.8) | 3.13 (2.89-3.36) |  | 4.2 (4.0-4.5) | 3.95 (3.75-4.17) |  | 0.82 (-0.42 to 2.08) |
| **Argentina** | 2363.3 (2213.9-2531.9) | 7.29 (6.83-7.81) |  | 3919.1 (3627.6-4192.0) | 7.07 (6.55-7.56) |  | -0.07 (-0.4 to 0.26) |
| **Armenia** | 156.1 (133.7-184.4) | 5.47 (4.68-6.47) |  | 328.2 (274.8-389.5) | 7.54 (6.32-8.96) |  | 1.11 (0.44 to 1.78) |
| **Australia** | 1356.3 (1286.1-1423.6) | 6.94 (6.57-7.28) |  | 4269.7 (3815.3-4638.0) | 9.49 (8.60-10.24) |  | 1.02 (0.8 to 1.24) |
| **Austria** | 907.9 (859.2-947.0) | 7.85 (7.43-8.18) |  | 1708.8 (1531.7-1839.3) | 9.56 (8.69-10.24) |  | 0.64 (0.26 to 1.02) |
| **Azerbaijan** | 111.7 (82.9-156.7) | 2.09 (1.56-2.92) |  | 354.6 (247.3-514.4) | 3.25 (2.33-4.66) |  | 1.43 (1.11 to 1.74) |
| **Bahamas** | 4.8 (4.3-5.3) | 2.96 (2.64-3.30) |  | 13.9 (11.3-16.9) | 3.32 (2.71-4.03) |  | 0.45 (-0.03 to 0.92) |
| **Bahrain** | 8.1 (6.9-9.3) | 4.56 (3.89-5.20) |  | 43.6 (33.3-60.3) | 4.83 (3.77-6.53) |  | 0.2 (-0.05 to 0.44) |
| **Bangladesh** | 378.9 (220.6-521.9) | 0.77 (0.45-1.05) |  | 1215.2 (817.8-1769.8) | 0.86 (0.59-1.25) |  | 0.47 (0.22 to 0.72) |
| **Barbados** | 14.0 (12.7-15.3) | 4.94 (4.50-5.37) |  | 27.3 (21.5-33.9) | 5.36 (4.23-6.66) |  | 0.28 (0.02 to 0.53) |
| **Belarus** | 572.6 (526.5-618.8) | 4.37 (4.03-4.71) |  | 977.5 (797.4-1194.6) | 6.21 (5.06-7.55) |  | 1.12 (0.67 to 1.56) |
| **Belgium** | 1000.2 (938.0-1051.3) | 6.59 (6.19-6.91) |  | 1670.9 (1494.7-1808.8) | 7.50 (6.83-8.09) |  | 0.32 (-0.44 to 1.09) |
| **Belize** | 2.4 (2.0-3.0) | 2.52 (2.16-3.20) |  | 12.4 (11.0-14.1) | 3.95 (3.50-4.49) |  | 1.52 (0.92 to 2.13) |
| **Benin** | 21.5 (17.7-25.7) | 1.06 (0.88-1.27) |  | 104.5 (73.5-142.3) | 1.94 (1.39-2.58) |  | 2 (1.88 to 2.13) |
| **Bermuda** | 4.9 (3.8-5.6) | 7.82 (6.11-9.05) |  | 8.7 (7.4-10.7) | 6.56 (5.58-8.03) |  | -0.7 (-1.29 to -0.11) |
| **Bhutan** | 1.9 (0.8-3.0) | 0.71 (0.31-1.13) |  | 6.8 (3.7-9.6) | 1.11 (0.61-1.57) |  | 1.47 (1.31 to 1.63) |
| **Bolivia (Plurinational State of)** | 125.5 (83.3-161.9) | 3.83 (2.54-4.91) |  | 431.9 (290.3-609.1) | 4.70 (3.15-6.62) |  | 0.66 (0.5 to 0.83) |
| **Bosnia and Herzegovina** | 216.2 (184.4-246.5) | 5.06 (4.32-5.79) |  | 392.8 (314.5-486.4) | 6.36 (5.07-7.90) |  | 0.69 (0.42 to 0.96) |
| **Botswana** | 14.7 (10.4-19.6) | 2.55 (1.83-3.33) |  | 51.2 (35.2-72.1) | 3.39 (2.40-4.59) |  | 0.87 (0.26 to 1.47) |
| **Brazil** | 3286.7 (3151.5-3390.6) | 3.60 (3.43-3.72) |  | 11450.3 (10764.7-11971.0) | 4.52 (4.24-4.74) |  | 0.8 (0.51 to 1.1) |
| **Brunei Darussalam** | 4.2 (3.3-5.2) | 3.96 (3.11-4.94) |  | 15.5 (12.6-18.7) | 4.36 (3.58-5.27) |  | 0.27 (0.07 to 0.47) |
| **Bulgaria** | 673.7 (613.3-740.4) | 5.50 (5.05-6.04) |  | 1103.2 (924.3-1296.0) | 8.20 (6.91-9.66) |  | 1.3 (0.64 to 1.97) |
| **Burkina Faso** | 40.6 (30.5-52.2) | 0.91 (0.69-1.17) |  | 162.6 (114.3-221.3) | 1.70 (1.21-2.32) |  | 2 (1.71 to 2.29) |
| **Burundi** | 39.3 (29.9-49.9) | 1.61 (1.24-2.03) |  | 64.1 (42.4-98.1) | 1.22 (0.82-1.83) |  | -0.91 (-1.09 to -0.73) |
| **Cabo Verde** | 1.2 (1.0-1.4) | 0.50 (0.41-0.60) |  | 28.5 (21.3-36.0) | 6.35 (4.70-8.08) |  | 8.69 (8.07 to 9.31) |
| **Cambodia** | 87.9 (62.3-128.2) | 1.80 (1.29-2.58) |  | 327.7 (243.8-423.9) | 2.49 (1.88-3.19) |  | 1.07 (0.98 to 1.17) |
| **Cameroon** | 72.7 (57.3-91.6) | 1.57 (1.25-1.97) |  | 377.9 (257.4-524.1) | 2.81 (1.96-3.89) |  | 1.89 (1.8 to 1.97) |
| **Canada** | 2769.6 (2597.2-2944.0) | 8.53 (8.00-9.06) |  | 7842.7 (6959.3-8616.0) | 10.65 (9.57-11.64) |  | 0.68 (0.41 to 0.94) |
| **Central African Republic** | 21.2 (14.4-27.1) | 1.71 (1.23-2.14) |  | 39.6 (28.1-55.6) | 1.60 (1.17-2.19) |  | -0.22 (-0.34 to -0.1) |
| **Chad** | 19.8 (14.8-25.5) | 0.69 (0.52-0.89) |  | 90.9 (68.7-116.7) | 1.49 (1.13-1.90) |  | 2.57 (2.49 to 2.65) |
| **Chile** | 408.3 (384.3-430.1) | 4.08 (3.84-4.30) |  | 1302.3 (1205.1-1404.7) | 5.08 (4.71-5.47) |  | 0.68 (0.19 to 1.17) |
| **China** | 31718.0 (26663.4-36996.8) | 3.55 (2.99-4.14) |  | 95523.6 (75562.6-116662.0) | 4.53 (3.60-5.50) |  | 0.8 (0.65 to 0.95) |
| **Colombia** | 697.2 (662.1-727.7) | 3.82 (3.62-3.99) |  | 1858.6 (1549.9-2208.4) | 3.37 (2.81-4.00) |  | -0.44 (-1.08 to 0.2) |
| **Comoros** | 4.0 (2.9-5.2) | 1.90 (1.42-2.50) |  | 10.3 (7.3-14.3) | 2.04 (1.44-2.82) |  | 0.22 (0.02 to 0.43) |
| **Congo** | 29.4 (18.9-40.2) | 2.63 (1.75-3.52) |  | 79.0 (51.9-114.2) | 2.70 (1.79-3.88) |  | 0.13 (-0.14 to 0.4) |
| **Cook Islands** | 0.3 (0.3-0.4) | 2.62 (2.23-3.08) |  | 0.8 (0.6-1.0) | 2.98 (2.34-3.71) |  | 0.45 (0.27 to 0.62) |
| **Costa Rica** | 52.9 (49.1-56.8) | 2.97 (2.75-3.19) |  | 233.6 (206.3-264.3) | 4.24 (3.75-4.80) |  | 1.13 (0.43 to 1.82) |
| **Coted'Ivoire** | 41.4 (32.4-53.1) | 0.98 (0.78-1.22) |  | 151.4 (105.2-215.7) | 1.27 (0.90-1.77) |  | 0.85 (0.67 to 1.04) |
| **Croatia** | 444.1 (403.9-481.1) | 7.32 (6.66-7.93) |  | 802.1 (693.9-925.6) | 9.02 (7.82-10.36) |  | 0.75 (-0.33 to 1.85) |
| **Cuba** | 429.6 (402.0-457.5) | 4.15 (3.90-4.42) |  | 770.6 (662.5-887.1) | 4.00 (3.44-4.60) |  | -0.16 (-0.72 to 0.39) |
| **Cyprus** | 32.1 (25.5-39.4) | 4.29 (3.40-5.28) |  | 111.4 (87.3-137.5) | 5.46 (4.30-6.68) |  | 0.77 (0.5 to 1.04) |
| **Czechia** | 1211.4 (1121.9-1314.7) | 8.85 (8.20-9.61) |  | 2023.3 (1723.2-2322.6) | 9.59 (8.21-10.92) |  | 0.3 (0.19 to 0.41) |
| **Democratic People's Republic of Korea** | 472.5 (333.1-659.2) | 2.70 (1.93-3.71) |  | 987.7 (631.8-1345.0) | 2.91 (1.86-3.93) |  | 0.25 (0.18 to 0.31) |
| **Democratic Republic of the Congo** | 246.8 (193.3-306.1) | 1.50 (1.18-1.84) |  | 573.5 (354.2-835.9) | 1.46 (0.91-2.14) |  | -0.1 (-0.22 to 0.02) |
| **Denmark** | 426.2 (389.4-473.4) | 5.47 (5.03-6.04) |  | 1029.6 (932.2-1107.6) | 8.74 (8.02-9.40) |  | 1.61 (1.05 to 2.18) |
| **Djibouti** | 2.4 (1.5-3.4) | 1.65 (1.02-2.29) |  | 14.1 (8.5-21.5) | 2.06 (1.28-3.06) |  | 0.73 (0.64 to 0.83) |
| **Dominica** | 2.4 (2.0-3.1) | 4.14 (3.39-5.22) |  | 4.9 (3.8-6.2) | 5.78 (4.60-7.35) |  | 1.1 (1.01 to 1.19) |
| **Dominican Republic** | 90.0 (72.6-111.8) | 2.34 (1.90-2.90) |  | 417.5 (329.8-532.6) | 4.11 (3.24-5.26) |  | 1.93 (1.51 to 2.35) |
| **Ecuador** | 132.9 (123.2-142.6) | 2.51 (2.32-2.69) |  | 542.9 (419.2-678.8) | 3.31 (2.57-4.13) |  | 0.81 (0.12 to 1.5) |
| **Egypt** | 465.5 (408.6-534.5) | 1.60 (1.41-1.82) |  | 2682.1 (2190.3-3261.8) | 4.04 (3.36-4.92) |  | 3.05 (2.84 to 3.25) |
| **El Salvador** | 56.0 (51.1-61.7) | 1.83 (1.66-2.01) |  | 212.4 (171.4-261.6) | 3.48 (2.80-4.29) |  | 2.21 (1.86 to 2.56) |
| **Equatorial Guinea** | 3.6 (2.5-4.7) | 1.74 (1.26-2.24) |  | 18.1 (10.4-26.9) | 3.29 (1.91-4.73) |  | 2.1 (1.75 to 2.44) |
| **Eritrea** | 19.7 (15.2-24.9) | 1.55 (1.21-1.93) |  | 51.9 (31.1-74.7) | 1.73 (1.08-2.45) |  | 0.34 (0.24 to 0.45) |
| **Estonia** | 142.4 (130.1-156.3) | 7.00 (6.37-7.67) |  | 241.4 (203.4-274.6) | 9.38 (7.99-10.64) |  | 0.97 (0.12 to 1.83) |
| **Eswatini** | 10.0 (6.8-14.5) | 3.35 (2.27-4.88) |  | 32.5 (19.4-49.1) | 5.38 (3.30-7.99) |  | 1.58 (1.34 to 1.82) |
| **Ethiopia** | 160.0 (75.6-249.7) | 0.75 (0.35-1.18) |  | 361.9 (217.1-564.5) | 0.80 (0.48-1.25) |  | 0.19 (0.12 to 0.26) |
| **Fiji** | 6.6 (5.4-7.8) | 1.71 (1.39-2.05) |  | 18.6 (13.7-24.6) | 2.33 (1.74-3.07) |  | 1 (0.91 to 1.1) |
| **Finland** | 724.1 (677.1-769.3) | 10.11 (9.46-10.69) |  | 1644.7 (1441.6-1831.9) | 12.78 (11.38-14.14) |  | 0.76 (0.33 to 1.19) |
| **France** | 4731.7 (4417.5-5148.4) | 5.97 (5.60-6.45) |  | 15742.4 (14030.4-17543.5) | 12.54 (11.42-13.87) |  | 2.43 (2.17 to 2.69) |
| **Gabon** | 15.9 (10.8-24.1) | 2.72 (1.83-4.14) |  | 41.8 (29.2-56.5) | 3.81 (2.70-5.09) |  | 1.1 (0.89 to 1.31) |
| **Gambia** | 3.5 (2.7-4.6) | 0.96 (0.74-1.23) |  | 15.1 (10.8-20.1) | 1.47 (1.04-1.93) |  | 1.43 (0.89 to 1.97) |
| **Georgia** | 101.4 (82.3-125.5) | 1.56 (1.27-1.92) |  | 295.5 (256.8-338.5) | 5.17 (4.49-5.92) |  | 4.24 (2.68 to 5.82) |
| **Germany** | 9935.0 (9288.8-10543.3) | 8.05 (7.55-8.53) |  | 25055.1 (22750.8-27293.9) | 14.11 (12.98-15.17) |  | 1.78 (1.55 to 2.02) |
| **Ghana** | 68.0 (46.4-96.0) | 1.03 (0.71-1.46) |  | 506.8 (364.3-708.4) | 2.90 (2.09-4.09) |  | 3.41 (3.29 to 3.52) |
| **Greece** | 1077.9 (1019.4-1125.4) | 7.11 (6.73-7.41) |  | 1845.8 (1682.4-1952.6) | 8.28 (7.67-8.71) |  | 0.47 (0.21 to 0.74) |
| **Greenland** | 5.2 (4.4-6.0) | 14.47 (12.24-16.65) |  | 8.6 (7.0-10.4) | 11.88 (9.75-14.38) |  | -0.58 (-0.84 to -0.32) |
| **Grenada** | 3.4 (3.0-3.9) | 4.90 (4.29-5.58) |  | 7.9 (6.9-8.9) | 6.86 (6.01-7.71) |  | 1.11 (0.7 to 1.54) |
| **Guam** | 2.1 (1.8-2.4) | 2.69 (2.34-3.08) |  | 6.8 (5.8-7.8) | 3.29 (2.82-3.77) |  | 0.59 (-0.39 to 1.57) |
| **Guatemala** | 51.8 (49.7-53.9) | 1.41 (1.35-1.47) |  | 271.5 (233.5-316.1) | 2.41 (2.08-2.80) |  | 1.51 (0.43 to 2.59) |
| **Guinea** | 22.3 (17.4-28.8) | 0.66 (0.52-0.85) |  | 64.1 (47.6-87.7) | 1.08 (0.81-1.46) |  | 1.6 (1.49 to 1.7) |
| **Guinea-Bissau** | 5.8 (3.7-7.8) | 1.38 (0.90-1.84) |  | 18.4 (13.0-24.7) | 2.31 (1.63-3.07) |  | 1.69 (1.62 to 1.76) |
| **Guyana** | 10.2 (9.1-11.5) | 2.57 (2.29-2.90) |  | 25.4 (19.5-32.9) | 3.77 (2.93-4.82) |  | 1.4 (0.76 to 2.03) |
| **Haiti** | 94.9 (61.4-124.4) | 2.82 (1.90-3.65) |  | 211.0 (143.2-294.0) | 2.78 (1.91-3.81) |  | 0.01 (-0.17 to 0.19) |
| **Honduras** | 42.8 (35.9-50.8) | 1.98 (1.66-2.34) |  | 233.6 (170.8-326.4) | 3.60 (2.62-5.04) |  | 1.97 (1.62 to 2.33) |
| **Hungary** | 1080.3 (988.6-1170.3) | 7.47 (6.84-8.08) |  | 1558.6 (1348.4-1772.1) | 8.37 (7.24-9.53) |  | 0.34 (-0.18 to 0.86) |
| **Iceland** | 20.1 (18.7-21.3) | 7.11 (6.62-7.54) |  | 45.5 (39.9-50.7) | 8.05 (7.15-8.96) |  | 0.39 (-0.42 to 1.2) |
| **India** | 3833.4 (3055.9-4660.6) | 0.77 (0.61-0.94) |  | 13559.4 (11819.6-15258.7) | 1.11 (0.96-1.24) |  | 1.17 (1.02 to 1.32) |
| **Indonesia** | 1814.9 (1418.1-2219.1) | 1.68 (1.29-2.06) |  | 7240.6 (5312.9-9255.6) | 2.84 (2.05-3.63) |  | 1.72 (1.64 to 1.79) |
| **Iran (Islamic Republic of)** | 397.6 (326.1-465.5) | 1.48 (1.22-1.72) |  | 2156.2 (1895.4-2384.1) | 2.73 (2.40-3.02) |  | 1.96 (1.72 to 2.21) |
| **Iraq** | 189.0 (131.2-256.2) | 2.28 (1.57-3.07) |  | 906.2 (668.1-1174.5) | 3.62 (2.68-4.64) |  | 1.53 (1.36 to 1.71) |
| **Ireland** | 292.6 (276.9-305.9) | 7.23 (6.84-7.55) |  | 505.4 (458.7-556.5) | 6.48 (5.88-7.12) |  | -0.3 (-0.89 to 0.29) |
| **Israel** | 325.9 (306.2-343.2) | 6.78 (6.35-7.14) |  | 878.5 (785.9-949.3) | 7.22 (6.49-7.75) |  | 0.25 (-0.28 to 0.79) |
| **Italy** | 6236.6 (5912.2-6465.9) | 7.15 (6.79-7.41) |  | 12099.3 (10582.3-13108.8) | 8.63 (7.77-9.22) |  | 0.56 (0.4 to 0.73) |
| **Jamaica** | 44.2 (39.8-51.2) | 2.50 (2.25-2.89) |  | 98.6 (76.1-128.9) | 3.19 (2.46-4.17) |  | 0.94 (0.11 to 1.78) |
| **Japan** | 15223.7 (14402.5-15856.8) | 8.99 (8.47-9.38) |  | 47433.7 (39192.9-52343.5) | 12.28 (10.64-13.21) |  | 1.04 (0.95 to 1.13) |
| **Jordan** | 26.1 (21.4-31.5) | 1.83 (1.50-2.20) |  | 197.8 (144.4-267.9) | 2.52 (1.86-3.36) |  | 1.08 (0.65 to 1.51) |
| **Kazakhstan** | 515.1 (413.4-683.2) | 3.88 (3.11-5.12) |  | 816.1 (685.1-954.0) | 4.30 (3.63-5.01) |  | 0.37 (-0.19 to 0.93) |
| **Kenya** | 95.5 (61.4-134.8) | 1.11 (0.71-1.56) |  | 524.2 (410.9-690.2) | 2.16 (1.69-2.81) |  | 2.16 (1.97 to 2.35) |
| **Kiribati** | 0.3 (0.2-0.3) | 0.69 (0.55-0.83) |  | 0.6 (0.5-0.9) | 0.87 (0.62-1.18) |  | 0.78 (0.72 to 0.84) |
| **Kuwait** | 15.4 (14.1-16.8) | 2.36 (2.13-2.55) |  | 109.4 (90.9-131.6) | 3.40 (2.77-4.12) |  | 1.2 (-0.8 to 3.25) |
| **Kyrgyzstan** | 90.8 (76.3-106.1) | 2.95 (2.48-3.43) |  | 204.7 (167.3-252.1) | 3.93 (3.21-4.82) |  | 0.95 (-0.37 to 2.28) |
| **Lao People's Democratic Republic** | 39.9 (23.1-63.1) | 1.78 (1.05-2.80) |  | 110.1 (78.4-149.8) | 2.21 (1.58-2.96) |  | 0.7 (0.63 to 0.77) |
| **Latvia** | 229.6 (209.4-250.0) | 6.41 (5.85-6.99) |  | 318.4 (271.3-369.7) | 8.55 (7.30-9.95) |  | 0.93 (0.45 to 1.42) |
| **Lebanon** | 57.4 (38.5-74.9) | 2.60 (1.78-3.38) |  | 199.6 (146.1-268.5) | 3.33 (2.44-4.47) |  | 0.82 (0.52 to 1.13) |
| **Lesotho** | 15.5 (11.6-20.8) | 1.81 (1.37-2.45) |  | 46.9 (30.1-67.5) | 4.19 (2.69-5.97) |  | 2.8 (2.54 to 3.06) |
| **Liberia** | 13.4 (9.7-17.9) | 1.14 (0.83-1.51) |  | 43.2 (23.9-66.8) | 1.89 (1.06-2.87) |  | 1.61 (1.35 to 1.88) |
| **Libya** | 76.1 (54.0-101.0) | 3.90 (2.80-5.14) |  | 325.4 (229.4-436.9) | 5.86 (4.07-7.75) |  | 1.3 (1 to 1.6) |
| **Lithuania** | 294.2 (267.9-318.4) | 6.51 (5.95-7.05) |  | 434.4 (377.3-488.9) | 8.09 (7.01-9.08) |  | 0.8 (-0.34 to 1.95) |
| **Luxembourg** | 38.4 (36.3-40.3) | 7.14 (6.74-7.48) |  | 74.7 (67.0-82.5) | 7.08 (6.39-7.81) |  | -0.07 (-0.52 to 0.39) |
| **Madagascar** | 73.2 (54.0-91.0) | 1.36 (1.00-1.70) |  | 154.2 (103.7-220.7) | 1.25 (0.85-1.75) |  | -0.33 (-0.54 to -0.12) |
| **Malawi** | 22.5 (17.3-27.8) | 0.55 (0.43-0.69) |  | 58.3 (41.0-81.9) | 0.74 (0.53-1.03) |  | 0.99 (0.85 to 1.12) |
| **Malaysia** | 125.0 (104.8-145.0) | 1.30 (1.09-1.51) |  | 591.4 (507.6-683.2) | 2.04 (1.75-2.36) |  | 1.58 (1.28 to 1.87) |
| **Maldives** | 1.4 (0.9-2.1) | 1.50 (0.98-2.16) |  | 5.0 (3.9-6.2) | 1.37 (1.07-1.68) |  | -0.35 (-0.55 to -0.15) |
| **Mali** | 58.0 (47.1-69.0) | 1.40 (1.14-1.66) |  | 166.6 (123.2-220.8) | 1.80 (1.34-2.36) |  | 0.85 (0.72 to 0.99) |
| **Malta** | 28.6 (26.7-30.6) | 6.67 (6.20-7.14) |  | 71.1 (63.2-79.6) | 7.78 (6.97-8.68) |  | 0.52 (0.26 to 0.79) |
| **Marshall Islands** | 0.3 (0.2-0.4) | 1.75 (1.44-2.12) |  | 1.0 (0.7-1.4) | 2.68 (1.93-3.66) |  | 1.4 (1.31 to 1.49) |
| **Mauritania** | 14.5 (11.3-17.9) | 1.42 (1.09-1.75) |  | 57.5 (41.5-76.3) | 2.61 (1.89-3.44) |  | 2 (1.78 to 2.21) |
| **Mauritius** | 26.3 (24.9-27.6) | 3.44 (3.25-3.61) |  | 74.3 (68.4-78.6) | 4.04 (3.74-4.26) |  | 0.52 (-0.71 to 1.77) |
| **Mexico** | 1734.2 (1695.0-1769.1) | 4.00 (3.89-4.09) |  | 4766.4 (4200.3-5342.0) | 3.70 (3.27-4.14) |  | -0.27 (-0.6 to 0.06) |
| **Micronesia (Federated States of)** | 1.1 (0.9-1.4) | 2.13 (1.71-2.77) |  | 2.4 (1.7-3.3) | 3.09 (2.21-4.13) |  | 1.2 (1.17 to 1.23) |
| **Monaco** | 6.0 (4.3-8.1) | 9.15 (6.56-12.32) |  | 10.3 (6.7-14.9) | 11.24 (7.30-16.51) |  | 0.66 (0.62 to 0.7) |
| **Mongolia** | 11.7 (8.9-15.2) | 1.07 (0.81-1.40) |  | 145.3 (109.2-191.3) | 5.72 (4.27-7.58) |  | 5.45 (5.12 to 5.79) |
| **Montenegro** | 37.6 (31.0-46.1) | 5.89 (4.83-7.22) |  | 74.6 (58.5-92.7) | 7.68 (6.04-9.52) |  | 0.91 (0.62 to 1.2) |
| **Morocco** | 110.2 (89.7-134.9) | 0.76 (0.62-0.92) |  | 459.2 (334.5-563.5) | 1.30 (0.96-1.60) |  | 1.77 (1.69 to 1.86) |
| **Mozambique** | 20.6 (17.0-25.0) | 0.36 (0.31-0.44) |  | 59.4 (44.3-77.7) | 0.56 (0.42-0.72) |  | 1.42 (1.3 to 1.53) |
| **Myanmar** | 438.2 (282.5-666.8) | 1.76 (1.16-2.65) |  | 1137.5 (831.2-1504.5) | 2.25 (1.66-2.94) |  | 0.82 (0.77 to 0.87) |
| **Namibia** | 5.4 (4.3-6.8) | 0.79 (0.63-0.98) |  | 16.5 (11.5-22.1) | 1.12 (0.80-1.47) |  | 1.16 (0.99 to 1.34) |
| **Nauru** | 0.2 (0.1-0.3) | 3.29 (2.13-5.08) |  | 0.3 (0.2-0.4) | 4.16 (2.54-5.78) |  | 0.76 (0.61 to 0.91) |
| **Nepal** | 61.9 (32.6-92.4) | 0.63 (0.32-0.93) |  | 224.9 (133.3-339.1) | 0.95 (0.57-1.42) |  | 1.35 (1.2 to 1.49) |
| **Netherlands** | 1170.0 (1106.7-1223.1) | 5.95 (5.64-6.22) |  | 2004.5 (1809.4-2145.4) | 5.85 (5.34-6.24) |  | -0.07 (-0.27 to 0.13) |
| **New Zealand** | 254.5 (236.9-269.9) | 6.50 (6.06-6.90) |  | 676.5 (609.8-733.6) | 8.08 (7.33-8.73) |  | 0.68 (0.4 to 0.95) |
| **Nicaragua** | 22.8 (19.7-26.2) | 1.41 (1.22-1.62) |  | 114.2 (90.7-141.0) | 2.27 (1.80-2.81) |  | 1.61 (1.29 to 1.94) |
| **Niger** | 22.4 (15.6-29.1) | 0.77 (0.54-0.99) |  | 101.3 (63.0-147.4) | 1.19 (0.76-1.70) |  | 1.45 (1.3 to 1.6) |
| **Nigeria** | 193.7 (130.9-246.3) | 0.44 (0.30-0.55) |  | 689.0 (512.7-920.1) | 0.74 (0.56-0.96) |  | 1.73 (1.65 to 1.81) |
| **Niue** | 0.0 (0.0-0.1) | 2.19 (1.60-2.79) |  | 0.1 (0.1-0.1) | 3.64 (2.76-4.72) |  | 1.67 (1.6 to 1.73) |
| **North Macedonia** | 93.1 (77.3-111.5) | 4.86 (4.04-5.81) |  | 209.2 (158.7-273.3) | 6.30 (4.82-8.16) |  | 0.86 (0.65 to 1.06) |
| **Northern Mariana Islands** | 0.5 (0.4-0.7) | 2.67 (2.15-3.36) |  | 2.9 (2.4-3.3) | 5.45 (4.58-6.39) |  | 2.29 (2.04 to 2.55) |
| **Norway** | 541.8 (505.2-567.0) | 8.04 (7.59-8.39) |  | 956.9 (858.2-1033.5) | 9.58 (8.68-10.28) |  | 0.59 (0.15 to 1.03) |
| **Oman** | 6.8 (4.8-9.6) | 0.95 (0.67-1.33) |  | 31.3 (22.3-42.3) | 1.44 (1.05-1.84) |  | 1.39 (0.96 to 1.81) |
| **Pakistan** | 389.2 (325.9-462.0) | 0.67 (0.57-0.80) |  | 1343.3 (1037.5-1786.7) | 1.04 (0.81-1.40) |  | 1.42 (1.31 to 1.53) |
| **Palau** | 0.6 (0.4-0.7) | 5.89 (4.53-7.46) |  | 1.4 (1.1-1.7) | 6.69 (5.33-8.22) |  | 0.41 (0.23 to 0.59) |
| **Palestine** | 28.5 (19.8-39.8) | 3.27 (2.29-4.51) |  | 102.2 (83.9-120.5) | 3.92 (3.23-4.61) |  | 0.54 (0.24 to 0.84) |
| **Panama** | 24.5 (23.0-26.0) | 1.61 (1.50-1.71) |  | 122.0 (97.5-145.1) | 2.77 (2.21-3.30) |  | 1.75 (0.99 to 2.52) |
| **Papua New Guinea** | 20.1 (13.4-29.8) | 1.04 (0.71-1.55) |  | 73.5 (51.6-107.4) | 1.35 (0.94-1.98) |  | 0.85 (0.82 to 0.88) |
| **Paraguay** | 37.9 (32.1-45.2) | 1.68 (1.42-2.00) |  | 231.4 (174.5-303.0) | 3.95 (3.00-5.16) |  | 2.91 (2.65 to 3.16) |
| **Peru** | 417.5 (339.0-520.0) | 3.42 (2.78-4.26) |  | 1342.1 (985.2-1792.5) | 3.99 (2.93-5.32) |  | 0.6 (-0.23 to 1.42) |
| **Philippines** | 584.2 (514.0-674.4) | 1.76 (1.54-2.07) |  | 2285.6 (1890.8-2714.8) | 2.60 (2.16-3.09) |  | 1.32 (1.18 to 1.47) |
| **Poland** | 2885.6 (2776.6-2979.7) | 6.58 (6.32-6.80) |  | 4428.6 (4036.4-4809.0) | 6.33 (5.76-6.87) |  | -0.23 (-0.31 to -0.14) |
| **Portugal** | 670.4 (634.2-704.1) | 4.93 (4.67-5.18) |  | 1226.7 (1108.0-1319.3) | 5.34 (4.90-5.74) |  | 0.24 (0 to 0.47) |
| **Puerto Rico** | 111.6 (104.5-118.3) | 3.07 (2.88-3.26) |  | 326.1 (268.5-384.7) | 5.02 (4.12-5.93) |  | 1.65 (0.73 to 2.57) |
| **Qatar** | 5.3 (4.2-6.6) | 4.54 (3.72-5.51) |  | 48.8 (35.3-68.4) | 4.71 (3.44-6.68) |  | -0.01 (-0.5 to 0.49) |
| **Republic of Korea** | 1985.9 (1689.2-2284.7) | 6.41 (5.44-7.41) |  | 7552.4 (5988.2-9197.4) | 8.02 (6.36-9.78) |  | 0.68 (0.42 to 0.93) |
| **Republic of Moldova** | 262.5 (243.3-283.9) | 5.81 (5.39-6.29) |  | 381.1 (338.4-427.6) | 6.46 (5.73-7.22) |  | 0.21 (-0.28 to 0.7) |
| **Romania** | 1355.3 (1250.9-1465.3) | 4.77 (4.41-5.15) |  | 2599.1 (2252.7-2941.4) | 7.46 (6.47-8.46) |  | 1.48 (0.98 to 1.98) |
| **Russian Federation** | 11634.0 (11370.3-11891.8) | 6.35 (6.21-6.50) |  | 16123.8 (14761.3-17434.7) | 6.84 (6.26-7.39) |  | 0.36 (-0.02 to 0.73) |
| **Rwanda** | 65.7 (50.8-80.5) | 2.16 (1.71-2.62) |  | 121.1 (81.7-178.6) | 1.84 (1.26-2.66) |  | -0.62 (-0.9 to -0.35) |
| **Saint Kitts and Nevis** | 1.7 (1.4-1.9) | 4.62 (3.77-5.31) |  | 3.5 (2.9-4.1) | 5.07 (4.20-5.90) |  | 0.5 (0.27 to 0.74) |
| **Saint Lucia** | 3.5 (3.1-3.8) | 4.05 (3.60-4.48) |  | 11.1 (9.2-13.5) | 4.63 (3.82-5.60) |  | 0.37 (-0.16 to 0.91) |
| **Saint Vincent and the Grenadines** | 2.8 (2.5-3.0) | 3.89 (3.56-4.24) |  | 6.2 (5.4-7.0) | 4.31 (3.80-4.92) |  | 0.31 (-0.12 to 0.74) |
| **Samoa** | 2.0 (1.7-2.4) | 2.36 (1.93-2.80) |  | 4.0 (3.1-5.1) | 2.70 (2.13-3.43) |  | 0.44 (0.35 to 0.53) |
| **San Marino** | 2.4 (2.0-2.8) | 6.83 (5.71-8.03) |  | 3.4 (2.2-4.8) | 4.79 (3.01-6.90) |  | -1.13 (-1.53 to -0.73) |
| **Sao Tome and Principe** | 0.3 (0.2-0.3) | 0.43 (0.35-0.50) |  | 0.8 (0.6-1.2) | 0.71 (0.54-0.98) |  | 1.64 (1.38 to 1.9) |
| **Saudi Arabia** | 75.3 (56.6-97.3) | 1.21 (0.92-1.55) |  | 548.3 (424.4-696.5) | 2.39 (1.93-2.95) |  | 2.22 (2.15 to 2.28) |
| **Senegal** | 37.6 (30.3-45.7) | 1.13 (0.92-1.37) |  | 175.4 (130.8-232.8) | 2.19 (1.65-2.86) |  | 2.19 (1.8 to 2.57) |
| **Serbia** | 633.8 (503.8-809.4) | 5.65 (4.49-7.16) |  | 1031.2 (808.2-1270.2) | 6.40 (4.97-7.98) |  | 0.47 (0.04 to 0.89) |
| **Seychelles** | 2.2 (1.9-2.5) | 3.84 (3.29-4.40) |  | 4.9 (4.1-5.9) | 4.12 (3.44-4.88) |  | 0.19 (0.02 to 0.36) |
| **Sierra Leone** | 18.4 (14.1-23.1) | 0.88 (0.68-1.10) |  | 62.7 (44.5-84.0) | 1.58 (1.14-2.08) |  | 1.9 (1.8 to 2) |
| **Singapore** | 93.9 (88.7-99.1) | 4.17 (3.93-4.41) |  | 459.4 (417.4-499.4) | 5.42 (4.92-5.91) |  | 0.91 (0.24 to 1.59) |
| **Slovakia** | 440.2 (366.8-535.7) | 7.35 (6.10-8.96) |  | 720.3 (570.8-912.5) | 7.68 (6.06-9.74) |  | 0.12 (-0.26 to 0.5) |
| **Slovenia** | 157.0 (146.6-167.7) | 6.37 (5.94-6.80) |  | 313.5 (266.8-361.6) | 7.20 (6.12-8.30) |  | 0.34 (-0.22 to 0.91) |
| **Solomon Islands** | 2.0 (1.2-2.8) | 1.36 (0.86-1.88) |  | 7.7 (5.6-10.5) | 2.04 (1.49-2.74) |  | 1.29 (1.04 to 1.54) |
| **Somalia** | 33.4 (19.9-53.6) | 1.26 (0.75-2.02) |  | 69.0 (35.6-114.5) | 1.02 (0.52-1.67) |  | -0.67 (-0.76 to -0.58) |
| **South Africa** | 627.8 (533.2-789.0) | 2.90 (2.44-3.71) |  | 2012.0 (1763.1-2246.0) | 4.24 (3.73-4.74) |  | 1.2 (0.79 to 1.61) |
| **South Sudan** | 44.8 (28.4-70.2) | 1.69 (1.06-2.65) |  | 74.5 (44.0-107.3) | 1.79 (1.07-2.57) |  | 0.22 (0.11 to 0.33) |
| **Spain** | 3230.4 (3028.5-3404.2) | 6.04 (5.67-6.37) |  | 7020.5 (6174.5-7648.5) | 7.49 (6.73-8.08) |  | 0.67 (0.33 to 1) |
| **Sri Lanka** | 116.1 (101.2-132.7) | 1.02 (0.89-1.17) |  | 314.5 (202.8-437.4) | 1.15 (0.75-1.60) |  | 0.35 (-0.24 to 0.93) |
| **Sudan** | 96.0 (61.9-148.4) | 0.99 (0.65-1.51) |  | 383.8 (241.7-591.4) | 1.83 (1.18-2.80) |  | 2.02 (1.92 to 2.11) |
| **Suriname** | 9.4 (7.9-11.0) | 3.59 (3.02-4.19) |  | 28.6 (21.7-36.7) | 4.42 (3.37-5.67) |  | 0.74 (0.36 to 1.11) |
| **Sweden** | 1009.1 (943.0-1062.8) | 6.97 (6.57-7.31) |  | 1321.1 (1149.1-1476.9) | 6.22 (5.44-6.92) |  | -0.39 (-0.88 to 0.1) |
| **Switzerland** | 587.7 (532.4-648.3) | 5.73 (5.24-6.27) |  | 1473.6 (1298.1-1644.7) | 8.12 (7.25-9.03) |  | 1.06 (0.52 to 1.6) |
| **Syrian Arab Republic** | 98.6 (73.4-125.9) | 1.78 (1.32-2.26) |  | 381.9 (270.8-521.6) | 2.80 (2.00-3.77) |  | 1.43 (1.15 to 1.71) |
| **Taiwan (Province of China)** | 580.6 (552.6-606.6) | 3.41 (3.23-3.57) |  | 2544.6 (2293.1-2730.4) | 6.19 (5.59-6.65) |  | 1.89 (1.31 to 2.47) |
| **Tajikistan** | 36.5 (25.1-52.1) | 1.28 (0.86-1.81) |  | 70.2 (48.2-100.3) | 1.11 (0.77-1.59) |  | -0.49 (-0.78 to -0.21) |
| **Thailand** | 1017.6 (857.7-1214.7) | 2.70 (2.28-3.19) |  | 4090.1 (3162.4-5341.1) | 3.87 (3.00-5.01) |  | 1.16 (0.97 to 1.36) |
| **Timor-Leste** | 3.7 (2.2-5.4) | 1.16 (0.71-1.68) |  | 13.8 (10.7-17.4) | 1.56 (1.21-1.97) |  | 0.97 (0.83 to 1.11) |
| **Togo** | 13.0 (9.8-16.7) | 1.00 (0.76-1.26) |  | 82.1 (56.0-111.4) | 2.02 (1.38-2.75) |  | 2.31 (2.22 to 2.4) |
| **Tokelau** | 0.0 (0.0-0.0) | 1.79 (1.25-2.46) |  | 0.0 (0.0-0.1) | 2.72 (1.91-3.68) |  | 1.38 (1.31 to 1.45) |
| **Tonga** | 1.7 (1.3-2.1) | 2.97 (2.33-3.78) |  | 3.3 (2.5-4.3) | 4.06 (3.05-5.25) |  | 1.01 (0.73 to 1.29) |
| **Trinidad and Tobago** | 31.5 (29.5-33.6) | 3.73 (3.49-4.00) |  | 76.7 (57.8-97.5) | 3.97 (3.00-5.05) |  | 0.34 (-0.07 to 0.75) |
| **Tunisia** | 73.5 (58.7-88.8) | 1.45 (1.16-1.75) |  | 288.3 (209.5-397.7) | 2.14 (1.56-2.94) |  | 1.27 (1.15 to 1.38) |
| **Turkey** | 1802.3 (1396.0-2330.8) | 4.98 (3.88-6.36) |  | 5847.4 (4544.9-7098.3) | 6.16 (4.79-7.44) |  | 0.68 (0.45 to 0.91) |
| **Turkmenistan** | 2.8 (2.4-3.1) | 0.13 (0.12-0.15) |  | 96.1 (72.2-129.4) | 2.19 (1.66-2.93) |  | 9.62 (7.08 to 12.21) |
| **Tuvalu** | 0.1 (0.1-0.1) | 1.66 (1.37-2.00) |  | 0.3 (0.2-0.4) | 2.59 (2.03-3.31) |  | 1.45 (1.39 to 1.51) |
| **Uganda** | 119.3 (92.3-153.3) | 1.80 (1.41-2.28) |  | 436.9 (303.8-608.2) | 2.78 (1.97-3.78) |  | 1.43 (1.33 to 1.53) |
| **Ukraine** | 3095.9 (2467.2-3997.1) | 4.35 (3.46-5.66) |  | 4248.2 (3170.3-5430.5) | 5.81 (4.32-7.49) |  | 0.99 (0.28 to 1.7) |
| **United Arab Emirates** | 32.0 (20.9-53.8) | 6.29 (4.05-10.60) |  | 294.3 (225.9-367.6) | 7.46 (5.99-9.21) |  | 0.35 (-1.43 to 2.17) |
| **United Kingdom** | 6138.1 (5891.0-6275.4) | 6.94 (6.69-7.08) |  | 9363.8 (8608.2-9753.0) | 7.30 (6.82-7.56) |  | 0.16 (-0.01 to 0.34) |
| **United Republic of Tanzania** | 191.3 (144.3-257.2) | 1.68 (1.28-2.24) |  | 490.4 (340.6-729.6) | 1.82 (1.30-2.67) |  | 0.27 (0.17 to 0.37) |
| **United States of America** | 25549.5 (24171.9-26355.9) | 8.15 (7.75-8.39) |  | 54302.8 (50464.3-56688.9) | 9.53 (8.94-9.92) |  | 0.49 (0.36 to 0.62) |
| **United States Virgin Islands** | 2.6 (2.2-3.1) | 3.02 (2.52-3.58) |  | 6.0 (4.5-7.9) | 3.68 (2.76-4.83) |  | 0.58 (-0.08 to 1.24) |
| **Uruguay** | 288.9 (268.7-308.1) | 7.45 (6.95-7.95) |  | 517.8 (470.4-564.6) | 9.71 (8.90-10.53) |  | 0.84 (0.63 to 1.04) |
| **Uzbekistan** | 141.1 (113.1-180.0) | 1.16 (0.91-1.51) |  | 535.6 (418.5-649.2) | 1.86 (1.47-2.26) |  | 1.58 (1.23 to 1.94) |
| **Vanuatu** | 0.9 (0.7-1.2) | 1.37 (1.06-1.75) |  | 3.7 (2.9-4.6) | 1.99 (1.60-2.44) |  | 1.17 (0.99 to 1.36) |
| **Venezuela (Bolivarian Republic of)** | 109.6 (104.3-114.6) | 1.06 (1.00-1.11) |  | 1075.4 (769.1-1412.1) | 3.55 (2.55-4.63) |  | 3.88 (3.14 to 4.61) |
| **Viet Nam** | 422.0 (331.9-540.9) | 1.03 (0.82-1.31) |  | 1727.5 (1267.0-2138.7) | 1.68 (1.24-2.06) |  | 1.62 (1.44 to 1.79) |
| **Yemen** | 48.5 (27.8-76.1) | 0.94 (0.54-1.43) |  | 201.9 (128.7-282.8) | 1.36 (0.87-1.89) |  | 1.23 (1.05 to 1.42) |
| **Zambia** | 60.0 (49.3-72.8) | 1.97 (1.64-2.38) |  | 178.1 (122.8-242.9) | 2.34 (1.67-3.15) |  | 0.55 (0.38 to 0.72) |
| **Zimbabwe** | 138.8 (111.5-167.4) | 3.39 (2.71-4.08) |  | 403.3 (298.2-522.9) | 5.42 (4.09-6.89) |  | 1.6 (1.22 to 1.98) |

*Abbreviations: ASR, age-standardized rate; AAPC, average annual percentage change; UI, uncertainty interval; CI, confidence interval.*

# Table S7. Case numbers and ASRs of mortality of pancreatic cancer in 1990 and 2021 for both sexes in 204 countries and AAPC from 1990 to 2021.

| **Location** | **1990** | |  | **2021** | |  | **1990-2021** |
| --- | --- | --- | --- | --- | --- | --- | --- |
|  | **Number**  **(95 % UI)** | **ASR**  **(95 % UI)** |  | **Number**  **(95 % UI)** | **ASR**  **(95 % UI)** |  | **AAPC**  **(95% CI)** |
| **Afghanistan** | 136.2 (70.5-257.0) | 2.00 (1.05-3.66) |  | 265.1 (162.9-466.2) | 2.90 (1.87-4.90) |  | 1.18 (1.11 to 1.26) |
| **Albania** | 82.3 (64.8-101.5) | 4.24 (3.36-5.22) |  | 236.8 (167.5-320.9) | 5.37 (3.81-7.29) |  | 0.7 (0.34 to 1.07) |
| **Algeria** | 144.7 (116.3-180.2) | 1.40 (1.14-1.73) |  | 580.8 (455.0-744.0) | 1.85 (1.47-2.33) |  | 0.91 (0.75 to 1.08) |
| **American Samoa** | 0.7 (0.6-0.9) | 3.57 (2.93-4.29) |  | 2.4 (2.0-2.9) | 5.22 (4.34-6.29) |  | 1.3 (1.03 to 1.56) |
| **Andorra** | 7.8 (5.5-10.9) | 13.78 (9.85-19.36) |  | 16.4 (11.3-21.9) | 10.39 (7.14-13.80) |  | -1.04 (-1.42 to -0.67) |
| **Angola** | 85.0 (63.5-115.3) | 2.32 (1.77-3.07) |  | 308.7 (187.8-447.7) | 2.84 (1.70-4.13) |  | 0.66 (0.49 to 0.82) |
| **Antigua and Barbuda** | 2.5 (2.3-2.7) | 4.52 (4.16-4.88) |  | 5.9 (5.6-6.2) | 5.75 (5.44-6.10) |  | 0.83 (-0.39 to 2.06) |
| **Argentina** | 3357.1 (3124.4-3599.2) | 10.52 (9.79-11.28) |  | 5587.2 (5105.7-5983.6) | 9.83 (9.02-10.51) |  | -0.19 (-0.46 to 0.07) |
| **Armenia** | 199.8 (170.8-238.6) | 7.52 (6.42-9.03) |  | 458.5 (380.3-540.0) | 10.46 (8.68-12.32) |  | 1.14 (0.36 to 1.91) |
| **Australia** | 1461.7 (1383.7-1528.9) | 7.45 (7.04-7.80) |  | 3699.9 (3292.2-3978.5) | 7.82 (7.05-8.38) |  | 0.14 (-0.25 to 0.53) |
| **Austria** | 1205.8 (1137.1-1264.0) | 9.92 (9.37-10.39) |  | 1914.2 (1690.1-2064.1) | 10.04 (9.01-10.80) |  | 0.06 (-0.36 to 0.48) |
| **Azerbaijan** | 137.8 (103.8-190.5) | 2.78 (2.09-3.79) |  | 445.4 (315.9-644.6) | 4.48 (3.25-6.32) |  | 1.56 (1.24 to 1.87) |
| **Bahamas** | 6.1 (5.5-6.8) | 4.03 (3.63-4.48) |  | 17.8 (14.6-21.6) | 4.53 (3.73-5.49) |  | 0.44 (0 to 0.88) |
| **Bahrain** | 10.0 (8.5-11.4) | 6.92 (5.89-7.97) |  | 49.0 (37.4-68.3) | 6.95 (5.44-9.26) |  | 0.02 (-0.3 to 0.33) |
| **Bangladesh** | 495.4 (287.7-673.8) | 1.09 (0.63-1.48) |  | 1636.4 (1112.3-2373.3) | 1.23 (0.84-1.74) |  | 0.54 (0.15 to 0.94) |
| **Barbados** | 21.5 (19.4-23.5) | 7.15 (6.48-7.79) |  | 39.9 (31.4-48.7) | 7.63 (6.01-9.31) |  | 0.21 (-0.11 to 0.53) |
| **Belarus** | 747.9 (690.7-805.7) | 5.73 (5.30-6.17) |  | 1175.5 (962.1-1443.6) | 7.28 (5.96-8.89) |  | 0.77 (0.29 to 1.24) |
| **Belgium** | 1426.4 (1318.3-1501.1) | 9.06 (8.39-9.53) |  | 2243.9 (1957.6-2443.8) | 9.16 (8.16-9.90) |  | -0.01 (-0.67 to 0.65) |
| **Belize** | 3.3 (2.9-4.2) | 3.59 (3.10-4.51) |  | 15.8 (14.0-17.9) | 5.47 (4.84-6.16) |  | 1.4 (0.84 to 1.97) |
| **Benin** | 29.8 (24.7-35.6) | 1.56 (1.29-1.86) |  | 136.6 (98.4-181.5) | 2.85 (2.09-3.75) |  | 1.98 (1.88 to 2.09) |
| **Bermuda** | 6.9 (5.4-8.0) | 11.48 (9.02-13.27) |  | 12.2 (10.3-14.8) | 8.50 (7.22-10.35) |  | -1.09 (-1.58 to -0.61) |
| **Bhutan** | 2.3 (1.0-3.7) | 1.00 (0.43-1.57) |  | 9.4 (5.3-13.3) | 1.61 (0.91-2.28) |  | 1.54 (1.4 to 1.69) |
| **Bolivia (Plurinational State of)** | 171.4 (113.0-220.4) | 5.68 (3.81-7.20) |  | 607.8 (402.3-846.0) | 7.04 (4.68-9.76) |  | 0.7 (0.54 to 0.86) |
| **Bosnia and Herzegovina** | 279.9 (238.1-321.6) | 7.06 (6.00-8.09) |  | 560.7 (451.1-691.4) | 8.81 (7.05-10.89) |  | 0.79 (0.58 to 1) |
| **Botswana** | 19.2 (13.8-25.6) | 3.73 (2.67-4.91) |  | 67.6 (47.5-91.7) | 5.07 (3.65-6.66) |  | 0.95 (0.35 to 1.56) |
| **Brazil** | 4365.1 (4161.2-4512.9) | 5.22 (4.92-5.42) |  | 15638.7 (14426.9-16440.8) | 6.29 (5.78-6.62) |  | 0.63 (0.32 to 0.94) |
| **Brunei Darussalam** | 5.2 (4.1-6.5) | 5.60 (4.39-7.00) |  | 18.2 (14.8-21.8) | 5.84 (4.75-7.08) |  | 0.06 (-0.15 to 0.27) |
| **Bulgaria** | 884.3 (808.5-973.3) | 7.37 (6.78-8.06) |  | 1533.2 (1293.5-1773.2) | 10.70 (9.04-12.38) |  | 1.24 (0.52 to 1.96) |
| **Burkina Faso** | 55.2 (41.7-70.7) | 1.35 (1.03-1.73) |  | 216.3 (153.5-297.0) | 2.51 (1.80-3.42) |  | 2.02 (1.86 to 2.18) |
| **Burundi** | 52.6 (40.7-66.5) | 2.33 (1.82-2.93) |  | 80.8 (53.6-123.9) | 1.78 (1.20-2.68) |  | -0.88 (-1.06 to -0.69) |
| **Cabo Verde** | 1.7 (1.4-2.0) | 0.71 (0.59-0.84) |  | 39.1 (28.4-49.6) | 9.29 (6.73-11.86) |  | 8.82 (8.04 to 9.61) |
| **Cambodia** | 110.1 (78.2-157.8) | 2.51 (1.79-3.55) |  | 411.1 (312.4-527.7) | 3.46 (2.67-4.35) |  | 1.06 (0.98 to 1.14) |
| **Cameroon** | 95.3 (75.4-119.4) | 2.30 (1.85-2.88) |  | 475.1 (327.2-663.7) | 4.07 (2.90-5.70) |  | 1.86 (1.75 to 1.97) |
| **Canada** | 2831.2 (2653.0-2975.5) | 8.66 (8.12-9.11) |  | 5928.7 (5248.2-6414.3) | 7.84 (6.99-8.47) |  | -0.36 (-0.64 to -0.08) |
| **Central African Republic** | 26.8 (18.8-33.8) | 2.47 (1.90-3.02) |  | 48.3 (34.8-67.0) | 2.30 (1.71-3.08) |  | -0.22 (-0.33 to -0.12) |
| **Chad** | 27.7 (21.0-35.7) | 1.01 (0.77-1.31) |  | 117.8 (89.3-150.2) | 2.18 (1.66-2.75) |  | 2.55 (2.45 to 2.64) |
| **Chile** | 583.8 (549.0-617.8) | 6.03 (5.66-6.38) |  | 1747.5 (1603.5-1894.1) | 6.71 (6.16-7.26) |  | 0.31 (-0.27 to 0.89) |
| **China** | 38883.0 (32790.3-45259.8) | 4.83 (4.10-5.61) |  | 119601.9 (95653.6-145218.1) | 5.72 (4.59-6.91) |  | 0.56 (0.33 to 0.79) |
| **Colombia** | 913.0 (866.7-952.9) | 5.45 (5.15-5.70) |  | 2515.0 (2082.9-2957.9) | 4.57 (3.78-5.37) |  | -0.62 (-1.22 to -0.02) |
| **Comoros** | 5.1 (3.9-6.8) | 2.73 (2.10-3.60) |  | 13.8 (9.7-19.2) | 2.97 (2.14-4.07) |  | 0.25 (0.09 to 0.42) |
| **Congo** | 38.3 (25.1-51.6) | 3.79 (2.63-5.05) |  | 97.5 (65.2-140.4) | 3.87 (2.58-5.48) |  | 0.1 (-0.11 to 0.31) |
| **Cook Islands** | 0.4 (0.4-0.5) | 3.77 (3.23-4.43) |  | 1.0 (0.8-1.3) | 3.93 (3.11-4.80) |  | 0.15 (0 to 0.3) |
| **Costa Rica** | 71.2 (65.9-76.5) | 4.19 (3.88-4.51) |  | 313.1 (277.1-352.5) | 5.72 (5.06-6.44) |  | 1.01 (0.31 to 1.71) |
| **Coted'Ivoire** | 51.8 (40.7-65.7) | 1.44 (1.17-1.76) |  | 189.3 (133.6-266.8) | 1.85 (1.36-2.52) |  | 0.82 (0.62 to 1.01) |
| **Croatia** | 514.1 (465.6-555.3) | 8.75 (7.91-9.45) |  | 840.0 (729.5-967.1) | 8.99 (7.81-10.35) |  | 0.12 (-0.74 to 0.99) |
| **Cuba** | 633.9 (594.2-676.1) | 6.15 (5.77-6.54) |  | 1061.5 (921.0-1225.7) | 5.33 (4.62-6.16) |  | -0.52 (-1.01 to -0.03) |
| **Cyprus** | 47.0 (37.5-57.6) | 6.77 (5.36-8.38) |  | 143.4 (112.3-176.9) | 7.06 (5.57-8.61) |  | 0.11 (-0.17 to 0.39) |
| **Czechia** | 1668.0 (1537.2-1821.2) | 11.97 (11.03-13.08) |  | 2610.4 (2226.5-3028.7) | 11.73 (9.99-13.51) |  | -0.06 (-0.16 to 0.04) |
| **Democratic People's Republic of Korea** | 564.8 (405.7-773.0) | 3.54 (2.60-4.80) |  | 1187.1 (756.4-1600.9) | 3.58 (2.27-4.80) |  | 0.03 (-0.02 to 0.08) |
| **Democratic Republic of the Congo** | 317.0 (252.1-393.3) | 2.19 (1.75-2.71) |  | 718.2 (441.5-1065.8) | 2.11 (1.28-3.18) |  | -0.13 (-0.25 to -0.01) |
| **Denmark** | 571.4 (515.6-643.6) | 6.94 (6.31-7.75) |  | 1289.7 (1162.0-1395.6) | 10.22 (9.25-11.02) |  | 1.24 (0.63 to 1.86) |
| **Djibouti** | 3.0 (1.8-4.2) | 2.40 (1.49-3.32) |  | 17.5 (10.8-26.2) | 3.02 (1.93-4.40) |  | 0.74 (0.67 to 0.81) |
| **Dominica** | 3.7 (3.0-4.9) | 6.25 (5.04-8.17) |  | 6.9 (5.5-8.9) | 8.43 (6.76-10.77) |  | 0.98 (0.87 to 1.1) |
| **Dominican Republic** | 117.1 (94.8-144.9) | 3.37 (2.73-4.15) |  | 562.4 (439.9-733.1) | 5.68 (4.44-7.44) |  | 1.77 (1.3 to 2.25) |
| **Ecuador** | 188.8 (175.0-203.4) | 3.79 (3.51-4.09) |  | 768.5 (601.2-952.1) | 4.80 (3.78-5.93) |  | 0.58 (-0.04 to 1.2) |
| **Egypt** | 568.1 (499.9-649.6) | 2.24 (1.98-2.56) |  | 3294.3 (2704.3-4010.0) | 5.66 (4.69-6.87) |  | 3.04 (2.83 to 3.26) |
| **El Salvador** | 74.2 (67.0-82.1) | 2.53 (2.28-2.81) |  | 289.2 (235.0-356.7) | 4.65 (3.77-5.74) |  | 2.07 (1.47 to 2.68) |
| **Equatorial Guinea** | 4.6 (3.3-6.0) | 2.49 (1.88-3.17) |  | 22.0 (13.0-31.9) | 4.71 (2.79-6.67) |  | 2.1 (1.8 to 2.41) |
| **Eritrea** | 23.8 (18.4-29.9) | 2.24 (1.75-2.75) |  | 63.9 (39.7-90.2) | 2.52 (1.60-3.51) |  | 0.38 (0.28 to 0.48) |
| **Estonia** | 180.5 (164.7-198.1) | 8.79 (8.00-9.67) |  | 286.9 (242.6-327.0) | 10.21 (8.67-11.61) |  | 0.55 (-0.47 to 1.58) |
| **Eswatini** | 13.0 (8.8-18.8) | 4.88 (3.31-7.06) |  | 40.8 (24.9-61.6) | 7.66 (4.76-11.28) |  | 1.48 (1.27 to 1.69) |
| **Ethiopia** | 207.0 (95.8-322.4) | 1.10 (0.50-1.77) |  | 476.8 (284.6-747.3) | 1.17 (0.70-1.83) |  | 0.23 (0.15 to 0.31) |
| **Fiji** | 8.1 (6.6-9.7) | 2.43 (1.97-2.93) |  | 24.1 (17.9-31.5) | 3.35 (2.54-4.29) |  | 1.02 (0.92 to 1.13) |
| **Finland** | 768.3 (718.7-807.9) | 10.57 (9.88-11.10) |  | 1498.0 (1318.1-1641.2) | 11.03 (9.84-12.01) |  | 0.13 (-0.32 to 0.59) |
| **France** | 6339.2 (5909.5-6888.4) | 7.57 (7.08-8.16) |  | 14126.3 (12312.7-15804.8) | 9.48 (8.46-10.52) |  | 0.74 (0.53 to 0.95) |
| **Gabon** | 21.8 (14.6-33.1) | 3.95 (2.63-6.01) |  | 53.8 (38.1-71.6) | 5.52 (3.92-7.22) |  | 1.09 (0.9 to 1.29) |
| **Gambia** | 4.6 (3.5-5.8) | 1.39 (1.08-1.73) |  | 19.7 (13.8-26.3) | 2.12 (1.49-2.84) |  | 1.42 (0.97 to 1.88) |
| **Georgia** | 130.0 (105.8-159.4) | 2.03 (1.65-2.48) |  | 413.4 (359.0-475.3) | 6.94 (6.03-7.96) |  | 4.38 (2.72 to 6.06) |
| **Germany** | 11522.8 (10607.5-12170.0) | 8.85 (8.19-9.35) |  | 21479.7 (19328.9-23314.5) | 10.54 (9.58-11.33) |  | 0.56 (0.16 to 0.96) |
| **Ghana** | 86.5 (59.2-122.7) | 1.48 (1.02-2.12) |  | 650.5 (465.7-917.0) | 4.23 (3.06-6.02) |  | 3.44 (3.32 to 3.55) |
| **Greece** | 1453.6 (1368.6-1521.8) | 9.47 (8.91-9.89) |  | 2587.3 (2328.5-2750.9) | 10.26 (9.41-10.82) |  | 0.25 (-0.08 to 0.58) |
| **Greenland** | 6.3 (5.4-7.3) | 20.44 (17.27-23.54) |  | 10.7 (8.7-13.0) | 15.89 (12.86-19.30) |  | -0.79 (-1.14 to -0.45) |
| **Grenada** | 5.1 (4.5-5.8) | 6.97 (6.11-7.90) |  | 10.8 (9.4-12.1) | 9.81 (8.60-10.91) |  | 1.17 (0.63 to 1.72) |
| **Guam** | 2.6 (2.2-3.0) | 3.93 (3.40-4.55) |  | 8.3 (7.0-9.6) | 3.97 (3.35-4.58) |  | -0.08 (-1.44 to 1.29) |
| **Guatemala** | 64.2 (61.4-67.2) | 2.07 (1.98-2.17) |  | 365.4 (314.3-420.9) | 3.40 (2.94-3.91) |  | 1.37 (0.34 to 2.41) |
| **Guinea** | 31.0 (24.2-39.1) | 0.97 (0.76-1.22) |  | 83.8 (62.6-113.0) | 1.56 (1.16-2.09) |  | 1.55 (1.44 to 1.65) |
| **Guinea-Bissau** | 7.5 (4.9-10.1) | 1.98 (1.35-2.63) |  | 22.4 (15.8-30.0) | 3.34 (2.38-4.40) |  | 1.71 (1.62 to 1.79) |
| **Guyana** | 13.5 (12.0-15.2) | 3.70 (3.29-4.16) |  | 32.7 (25.3-41.5) | 5.22 (4.10-6.57) |  | 1.27 (0.65 to 1.89) |
| **Haiti** | 124.6 (83.2-161.7) | 4.13 (2.88-5.32) |  | 272.1 (185.5-373.0) | 4.07 (2.79-5.55) |  | 0 (-0.06 to 0.06) |
| **Honduras** | 55.0 (45.9-65.0) | 2.78 (2.33-3.29) |  | 317.5 (229.1-443.7) | 5.24 (3.74-7.34) |  | 2.11 (1.7 to 2.52) |
| **Hungary** | 1505.7 (1371.5-1638.9) | 10.27 (9.37-11.15) |  | 2195.6 (1904.4-2495.5) | 11.09 (9.61-12.59) |  | 0.29 (0.07 to 0.51) |
| **Iceland** | 26.8 (24.7-28.5) | 9.19 (8.48-9.77) |  | 56.5 (48.6-62.9) | 9.32 (8.15-10.39) |  | 0.01 (-0.81 to 0.84) |
| **India** | 4814.1 (3825.8-5860.1) | 1.08 (0.85-1.32) |  | 17917.6 (15614.9-20103.5) | 1.55 (1.35-1.74) |  | 1.19 (0.96 to 1.42) |
| **Indonesia** | 2192.6 (1686.0-2684.0) | 2.30 (1.74-2.83) |  | 8968.9 (6407.3-11502.9) | 3.95 (2.80-5.08) |  | 1.77 (1.72 to 1.82) |
| **Iran (Islamic Republic of)** | 492.8 (403.7-575.6) | 2.12 (1.73-2.46) |  | 2676.1 (2353.5-2958.3) | 3.65 (3.21-4.04) |  | 1.75 (1.49 to 2.01) |
| **Iraq** | 239.5 (164.2-321.4) | 3.08 (2.11-4.12) |  | 1096.9 (804.2-1406.0) | 4.93 (3.66-6.24) |  | 1.57 (1.33 to 1.82) |
| **Ireland** | 411.0 (386.1-431.0) | 9.97 (9.37-10.44) |  | 630.2 (560.8-697.1) | 7.71 (6.88-8.52) |  | -0.79 (-1.23 to -0.36) |
| **Israel** | 466.4 (436.0-494.1) | 9.66 (8.99-10.25) |  | 1196.4 (1044.5-1300.8) | 9.33 (8.22-10.12) |  | -0.09 (-0.57 to 0.39) |
| **Italy** | 8258.9 (7746.3-8582.4) | 9.16 (8.59-9.51) |  | 14509.3 (12520.6-15729.1) | 9.29 (8.23-9.97) |  | 0 (-0.2 to 0.2) |
| **Jamaica** | 66.0 (59.7-74.8) | 3.64 (3.29-4.13) |  | 137.9 (108.4-174.7) | 4.43 (3.48-5.62) |  | 0.79 (0 to 1.59) |
| **Japan** | 15083.6 (14195.6-15595.1) | 8.96 (8.39-9.28) |  | 42065.0 (35527.2-45866.9) | 10.28 (9.08-10.97) |  | 0.42 (0.22 to 0.62) |
| **Jordan** | 31.6 (25.9-38.1) | 2.54 (2.08-3.04) |  | 231.0 (168.8-316.2) | 3.37 (2.50-4.58) |  | 0.97 (0.57 to 1.37) |
| **Kazakhstan** | 646.9 (521.6-836.7) | 5.16 (4.17-6.63) |  | 1009.0 (852.5-1175.8) | 5.65 (4.79-6.54) |  | 0.33 (-0.16 to 0.82) |
| **Kenya** | 125.5 (80.4-177.7) | 1.61 (1.03-2.28) |  | 668.1 (523.0-871.9) | 3.13 (2.45-4.00) |  | 2.17 (1.98 to 2.35) |
| **Kiribati** | 0.3 (0.3-0.4) | 1.01 (0.79-1.23) |  | 0.8 (0.6-1.1) | 1.29 (0.94-1.73) |  | 0.81 (0.75 to 0.88) |
| **Kuwait** | 17.3 (15.8-18.8) | 3.20 (2.87-3.48) |  | 115.3 (95.4-139.9) | 4.34 (3.57-5.28) |  | 0.97 (-1.09 to 3.06) |
| **Kyrgyzstan** | 116.8 (98.5-137.0) | 3.97 (3.35-4.64) |  | 246.0 (201.4-300.1) | 5.20 (4.28-6.33) |  | 0.66 (-0.51 to 1.85) |
| **Lao People's Democratic Republic** | 50.5 (29.6-79.9) | 2.48 (1.50-3.87) |  | 136.0 (98.4-183.9) | 3.07 (2.25-4.05) |  | 0.7 (0.63 to 0.77) |
| **Latvia** | 316.4 (287.3-345.3) | 8.75 (7.94-9.54) |  | 437.2 (373.9-501.8) | 10.87 (9.30-12.58) |  | 0.72 (0.23 to 1.22) |
| **Lebanon** | 75.5 (51.9-98.0) | 3.69 (2.58-4.75) |  | 272.4 (198.4-368.0) | 4.42 (3.23-5.97) |  | 0.61 (0.32 to 0.9) |
| **Lesotho** | 21.7 (16.4-29.0) | 2.69 (2.02-3.63) |  | 62.6 (40.9-89.8) | 6.11 (4.02-8.71) |  | 2.77 (2.45 to 3.08) |
| **Liberia** | 18.6 (13.5-24.6) | 1.69 (1.26-2.22) |  | 53.5 (29.9-81.4) | 2.75 (1.57-4.16) |  | 1.54 (1.27 to 1.82) |
| **Libya** | 98.1 (70.4-129.0) | 5.47 (3.98-7.18) |  | 389.7 (273.4-514.3) | 7.87 (5.61-10.40) |  | 1.16 (0.88 to 1.44) |
| **Lithuania** | 391.1 (356.8-423.2) | 8.62 (7.87-9.28) |  | 594.4 (515.4-670.8) | 10.24 (8.93-11.52) |  | 0.67 (-0.35 to 1.7) |
| **Luxembourg** | 53.1 (50.1-55.8) | 9.66 (9.12-10.17) |  | 96.8 (86.1-106.7) | 8.79 (7.87-9.69) |  | -0.33 (-0.84 to 0.18) |
| **Madagascar** | 94.9 (70.4-119.0) | 1.95 (1.44-2.45) |  | 186.5 (127.0-264.5) | 1.79 (1.22-2.49) |  | -0.31 (-0.55 to -0.06) |
| **Malawi** | 29.5 (22.9-36.8) | 0.81 (0.64-1.02) |  | 75.0 (53.6-103.4) | 1.09 (0.78-1.47) |  | 0.97 (0.82 to 1.12) |
| **Malaysia** | 163.0 (134.9-190.3) | 1.84 (1.51-2.17) |  | 757.3 (646.5-879.7) | 2.80 (2.37-3.26) |  | 1.43 (1.16 to 1.7) |
| **Maldives** | 1.8 (1.1-2.6) | 2.14 (1.43-3.01) |  | 5.9 (4.6-7.3) | 1.92 (1.52-2.34) |  | -0.41 (-0.61 to -0.2) |
| **Mali** | 76.4 (62.3-91.2) | 2.04 (1.68-2.44) |  | 216.9 (159.9-286.9) | 2.64 (1.99-3.42) |  | 0.86 (0.72 to 1) |
| **Malta** | 39.4 (36.4-42.3) | 9.27 (8.55-9.96) |  | 94.3 (82.6-106.3) | 9.39 (8.34-10.55) |  | 0.08 (-0.16 to 0.32) |
| **Marshall Islands** | 0.4 (0.3-0.5) | 2.52 (2.08-3.04) |  | 1.2 (0.9-1.7) | 3.76 (2.77-5.07) |  | 1.3 (1.21 to 1.4) |
| **Mauritania** | 20.0 (15.4-24.6) | 2.08 (1.60-2.54) |  | 77.8 (56.3-103.3) | 3.84 (2.79-5.11) |  | 2 (1.81 to 2.2) |
| **Mauritius** | 33.3 (31.5-35.1) | 4.78 (4.52-5.04) |  | 95.1 (88.4-100.3) | 5.19 (4.81-5.46) |  | 0.28 (-0.51 to 1.08) |
| **Mexico** | 2343.8 (2279.0-2398.6) | 5.90 (5.71-6.06) |  | 6352.7 (5615.1-7090.2) | 5.12 (4.53-5.71) |  | -0.49 (-0.83 to -0.15) |
| **Micronesia (Federated States of)** | 1.4 (1.1-1.8) | 3.07 (2.47-3.99) |  | 3.0 (2.2-4.0) | 4.36 (3.10-5.78) |  | 1.13 (1.1 to 1.17) |
| **Monaco** | 8.4 (6.0-11.5) | 11.61 (8.29-15.80) |  | 14.0 (9.2-20.4) | 13.49 (8.88-19.65) |  | 0.48 (0.44 to 0.53) |
| **Mongolia** | 15.6 (11.9-20.4) | 1.50 (1.14-1.97) |  | 177.0 (132.4-235.6) | 7.88 (5.86-10.51) |  | 5.41 (5.05 to 5.76) |
| **Montenegro** | 47.8 (38.9-58.4) | 7.72 (6.26-9.44) |  | 103.1 (81.2-126.5) | 10.66 (8.45-12.98) |  | 1.1 (0.73 to 1.47) |
| **Morocco** | 148.2 (121.2-181.1) | 1.08 (0.88-1.31) |  | 610.4 (450.4-748.5) | 1.83 (1.36-2.23) |  | 1.73 (1.64 to 1.82) |
| **Mozambique** | 29.6 (24.8-35.8) | 0.59 (0.50-0.71) |  | 82.8 (61.7-107.6) | 0.90 (0.68-1.17) |  | 1.4 (1.29 to 1.5) |
| **Myanmar** | 551.0 (367.1-831.7) | 2.43 (1.64-3.60) |  | 1466.1 (1086.3-1923.6) | 3.12 (2.36-4.07) |  | 0.81 (0.76 to 0.86) |
| **Namibia** | 7.0 (5.6-8.7) | 1.12 (0.90-1.38) |  | 20.9 (14.8-27.4) | 1.57 (1.16-2.01) |  | 1.12 (0.96 to 1.28) |
| **Nauru** | 0.2 (0.1-0.3) | 4.67 (3.15-6.99) |  | 0.3 (0.2-0.4) | 5.78 (3.61-7.90) |  | 0.69 (0.54 to 0.84) |
| **Nepal** | 79.7 (41.4-118.4) | 0.90 (0.46-1.32) |  | 304.9 (182.3-457.5) | 1.37 (0.82-2.06) |  | 1.37 (1.26 to 1.48) |
| **Netherlands** | 1803.9 (1690.1-1894.8) | 8.89 (8.35-9.33) |  | 3194.4 (2853.4-3452.0) | 8.70 (7.82-9.37) |  | -0.09 (-0.28 to 0.1) |
| **New Zealand** | 285.3 (265.3-301.6) | 7.20 (6.68-7.61) |  | 627.4 (561.0-678.6) | 7.22 (6.49-7.80) |  | -0.03 (-0.51 to 0.45) |
| **Nicaragua** | 29.2 (25.4-33.6) | 1.99 (1.72-2.31) |  | 147.7 (116.5-183.1) | 3.12 (2.48-3.86) |  | 1.46 (1.04 to 1.87) |
| **Niger** | 28.8 (20.4-37.3) | 1.13 (0.80-1.44) |  | 133.2 (84.8-191.2) | 1.78 (1.16-2.51) |  | 1.51 (1.38 to 1.65) |
| **Nigeria** | 273.2 (186.6-343.3) | 0.67 (0.46-0.84) |  | 922.9 (700.5-1193.1) | 1.13 (0.89-1.42) |  | 1.7 (1.63 to 1.78) |
| **Niue** | 0.1 (0.1-0.1) | 3.16 (2.36-4.01) |  | 0.1 (0.1-0.1) | 5.07 (3.88-6.53) |  | 1.54 (1.48 to 1.59) |
| **North Macedonia** | 124.8 (103.5-149.4) | 6.86 (5.69-8.24) |  | 291.2 (222.5-376.7) | 9.12 (7.11-11.57) |  | 0.91 (0.65 to 1.17) |
| **Northern Mariana Islands** | 0.6 (0.5-0.7) | 3.79 (3.08-4.72) |  | 3.6 (3.0-4.2) | 7.66 (6.42-9.03) |  | 2.18 (1.94 to 2.42) |
| **Norway** | 624.2 (583.1-648.3) | 8.82 (8.28-9.14) |  | 966.9 (867.6-1028.8) | 9.17 (8.32-9.72) |  | 0.26 (0.19 to 0.32) |
| **Oman** | 8.5 (6.0-11.8) | 1.34 (0.94-1.85) |  | 34.6 (25.0-44.6) | 1.95 (1.45-2.44) |  | 1.26 (0.82 to 1.71) |
| **Pakistan** | 532.3 (446.4-628.6) | 0.98 (0.82-1.16) |  | 1724.7 (1339.2-2312.2) | 1.52 (1.19-2.05) |  | 1.41 (1.3 to 1.51) |
| **Palau** | 0.8 (0.6-1.0) | 9.18 (7.11-11.67) |  | 1.9 (1.5-2.3) | 10.46 (8.40-12.93) |  | 0.38 (0.14 to 0.63) |
| **Palestine** | 38.9 (27.5-53.2) | 4.81 (3.44-6.52) |  | 125.8 (104.2-148.6) | 5.51 (4.55-6.52) |  | 0.41 (0.23 to 0.6) |
| **Panama** | 32.9 (30.8-35.1) | 2.28 (2.12-2.43) |  | 170.3 (134.3-203.0) | 3.85 (3.04-4.59) |  | 1.75 (0.83 to 2.68) |
| **Papua New Guinea** | 24.8 (16.7-37.1) | 1.50 (1.04-2.24) |  | 89.5 (62.0-132.1) | 1.93 (1.34-2.85) |  | 0.83 (0.69 to 0.97) |
| **Paraguay** | 52.1 (43.9-62.2) | 2.45 (2.07-2.93) |  | 318.8 (242.1-412.9) | 5.68 (4.32-7.34) |  | 2.88 (2.6 to 3.15) |
| **Peru** | 570.3 (467.5-715.2) | 4.97 (4.10-6.23) |  | 1840.1 (1349.0-2433.6) | 5.57 (4.09-7.35) |  | 0.46 (-0.38 to 1.3) |
| **Philippines** | 682.4 (597.6-804.4) | 2.40 (2.09-2.87) |  | 2816.2 (2344.7-3360.1) | 3.51 (2.95-4.16) |  | 1.24 (1.01 to 1.47) |
| **Poland** | 4099.7 (3928.1-4246.1) | 9.43 (9.03-9.77) |  | 6783.9 (6170.2-7341.5) | 9.25 (8.43-10.01) |  | -0.19 (-0.28 to -0.11) |
| **Portugal** | 1006.8 (946.5-1065.1) | 7.39 (6.93-7.82) |  | 1919.8 (1702.5-2084.1) | 7.46 (6.73-8.04) |  | 0.01 (-0.18 to 0.19) |
| **Puerto Rico** | 163.7 (153.2-174.4) | 4.52 (4.23-4.82) |  | 464.2 (382.7-545.2) | 6.32 (5.22-7.44) |  | 1.14 (0.37 to 1.9) |
| **Qatar** | 5.9 (4.8-7.2) | 6.87 (5.65-8.26) |  | 46.3 (33.1-67.2) | 6.47 (4.77-9.10) |  | -0.34 (-0.9 to 0.22) |
| **Republic of Korea** | 2389.7 (2043.1-2752.5) | 8.53 (7.26-9.94) |  | 7101.2 (5595.6-8633.0) | 7.51 (5.88-9.14) |  | -0.44 (-0.57 to -0.31) |
| **Republic of Moldova** | 333.2 (307.2-362.2) | 7.70 (7.08-8.38) |  | 478.2 (423.9-536.0) | 7.96 (7.06-8.91) |  | -0.02 (-0.49 to 0.45) |
| **Romania** | 1771.1 (1638.1-1908.4) | 6.35 (5.85-6.83) |  | 3614.7 (3151.6-4108.5) | 9.76 (8.49-11.08) |  | 1.42 (1.05 to 1.78) |
| **Russian Federation** | 14910.6 (14521.5-15253.3) | 8.25 (8.03-8.46) |  | 21995.8 (20252.0-23826.5) | 9.12 (8.40-9.88) |  | 0.42 (0.08 to 0.76) |
| **Rwanda** | 84.5 (65.9-102.7) | 3.11 (2.52-3.74) |  | 157.1 (106.2-232.9) | 2.72 (1.87-3.96) |  | -0.42 (-0.52 to -0.33) |
| **Saint Kitts and Nevis** | 2.6 (2.2-3.0) | 6.88 (5.74-7.85) |  | 4.8 (3.9-5.5) | 7.57 (6.31-8.66) |  | 0.27 (-0.44 to 0.98) |
| **Saint Lucia** | 5.0 (4.4-5.5) | 6.07 (5.38-6.70) |  | 15.7 (12.9-18.8) | 6.60 (5.44-7.91) |  | 0.12 (-0.41 to 0.66) |
| **Saint Vincent and the Grenadines** | 4.0 (3.6-4.4) | 5.71 (5.21-6.25) |  | 8.5 (7.5-9.7) | 6.07 (5.38-6.87) |  | 0.17 (-0.31 to 0.64) |
| **Samoa** | 2.7 (2.2-3.2) | 3.44 (2.84-4.04) |  | 5.2 (4.1-6.6) | 3.77 (3.00-4.76) |  | 0.29 (0.21 to 0.38) |
| **San Marino** | 3.2 (2.7-3.8) | 8.92 (7.49-10.52) |  | 4.5 (2.9-6.3) | 5.66 (3.67-8.09) |  | -1.61 (-2.01 to -1.21) |
| **Sao Tome and Principe** | 0.4 (0.3-0.5) | 0.63 (0.51-0.74) |  | 1.1 (0.8-1.4) | 1.03 (0.80-1.38) |  | 1.61 (1.36 to 1.86) |
| **Saudi Arabia** | 93.7 (71.1-119.9) | 1.72 (1.31-2.18) |  | 566.2 (446.4-716.7) | 3.16 (2.54-3.92) |  | 1.96 (1.9 to 2.03) |
| **Senegal** | 50.4 (41.4-61.3) | 1.64 (1.35-1.99) |  | 233.2 (177.4-303.9) | 3.21 (2.45-4.11) |  | 2.2 (1.86 to 2.53) |
| **Serbia** | 869.1 (689.3-1100.0) | 8.38 (6.62-10.55) |  | 1533.0 (1207.3-1877.8) | 9.08 (7.15-11.13) |  | 0.34 (-0.08 to 0.77) |
| **Seychelles** | 2.9 (2.5-3.3) | 5.16 (4.39-5.89) |  | 6.3 (5.2-7.4) | 5.54 (4.60-6.56) |  | 0.07 (-0.25 to 0.4) |
| **Sierra Leone** | 25.8 (20.3-31.8) | 1.31 (1.04-1.60) |  | 81.9 (59.0-109.2) | 2.31 (1.68-3.02) |  | 1.86 (1.74 to 1.97) |
| **Singapore** | 110.8 (104.6-117.2) | 5.25 (4.95-5.53) |  | 431.4 (390.7-466.0) | 5.10 (4.60-5.50) |  | 0.08 (-0.12 to 0.28) |
| **Slovakia** | 580.0 (485.3-694.1) | 9.64 (8.05-11.59) |  | 962.3 (763.3-1208.9) | 9.99 (7.93-12.58) |  | 0.08 (-0.27 to 0.44) |
| **Slovenia** | 208.9 (194.4-223.4) | 8.45 (7.86-9.04) |  | 392.7 (330.8-452.8) | 8.45 (7.13-9.73) |  | -0.02 (-0.44 to 0.4) |
| **Solomon Islands** | 2.5 (1.5-3.4) | 1.97 (1.31-2.68) |  | 9.4 (6.9-12.7) | 2.84 (2.12-3.84) |  | 1.18 (0.96 to 1.4) |
| **Somalia** | 40.7 (24.4-65.1) | 1.81 (1.08-2.90) |  | 84.7 (42.9-140.9) | 1.47 (0.75-2.45) |  | -0.67 (-0.74 to -0.6) |
| **South Africa** | 807.4 (672.0-1039.4) | 4.07 (3.36-5.28) |  | 2686.2 (2360.3-3002.2) | 6.12 (5.38-6.84) |  | 1.3 (0.92 to 1.68) |
| **South Sudan** | 60.9 (39.0-93.8) | 2.44 (1.55-3.76) |  | 93.9 (55.6-136.3) | 2.58 (1.55-3.69) |  | 0.18 (0.07 to 0.29) |
| **Spain** | 3877.6 (3625.8-4096.7) | 7.07 (6.62-7.45) |  | 7663.8 (6660.1-8356.8) | 7.58 (6.68-8.19) |  | 0.21 (0.08 to 0.34) |
| **Sri Lanka** | 146.2 (127.0-167.3) | 1.44 (1.25-1.66) |  | 399.6 (261.7-553.8) | 1.49 (0.98-2.07) |  | 0.05 (-0.54 to 0.64) |
| **Sudan** | 125.8 (82.4-191.9) | 1.41 (0.93-2.11) |  | 474.7 (306.3-738.4) | 2.56 (1.66-3.95) |  | 1.96 (1.88 to 2.04) |
| **Suriname** | 12.6 (10.6-14.6) | 5.11 (4.31-5.91) |  | 38.2 (28.9-49.4) | 6.09 (4.61-7.88) |  | 0.64 (0.22 to 1.06) |
| **Sweden** | 1584.6 (1464.9-1681.4) | 10.13 (9.43-10.70) |  | 2211.2 (1906.0-2491.1) | 9.44 (8.20-10.57) |  | -0.26 (-0.77 to 0.25) |
| **Switzerland** | 666.9 (601.6-740.9) | 6.26 (5.69-6.92) |  | 1544.6 (1336.1-1726.7) | 7.97 (7.03-8.86) |  | 0.72 (0.23 to 1.22) |
| **Syrian Arab Republic** | 123.6 (91.5-157.0) | 2.48 (1.84-3.16) |  | 471.5 (335.9-641.0) | 3.80 (2.73-5.11) |  | 1.32 (1.04 to 1.6) |
| **Taiwan (Province of China)** | 666.8 (633.9-696.9) | 4.25 (4.03-4.47) |  | 3030.5 (2748.8-3259.4) | 7.10 (6.43-7.64) |  | 1.61 (1.09 to 2.13) |
| **Tajikistan** | 47.5 (31.4-67.8) | 1.79 (1.16-2.62) |  | 85.7 (59.7-121.8) | 1.59 (1.11-2.24) |  | -0.43 (-0.83 to -0.04) |
| **Thailand** | 1270.2 (1071.8-1501.0) | 3.76 (3.18-4.45) |  | 5143.4 (3994.5-6597.7) | 4.73 (3.69-6.03) |  | 0.76 (0.57 to 0.94) |
| **Timor-Leste** | 4.3 (2.6-6.3) | 1.65 (1.00-2.36) |  | 18.3 (14.2-23.3) | 2.19 (1.71-2.79) |  | 0.95 (0.76 to 1.14) |
| **Togo** | 16.7 (12.7-21.1) | 1.45 (1.12-1.82) |  | 103.0 (69.8-141.0) | 2.93 (2.01-3.99) |  | 2.3 (2.23 to 2.37) |
| **Tokelau** | 0.0 (0.0-0.0) | 2.61 (1.87-3.60) |  | 0.1 (0.0-0.1) | 3.74 (2.65-5.06) |  | 1.18 (1.13 to 1.22) |
| **Tonga** | 2.2 (1.8-2.9) | 4.28 (3.38-5.46) |  | 4.5 (3.4-5.8) | 5.70 (4.35-7.27) |  | 0.92 (0.61 to 1.22) |
| **Trinidad and Tobago** | 43.6 (40.7-46.7) | 5.42 (5.04-5.82) |  | 105.1 (79.9-132.6) | 5.45 (4.15-6.87) |  | 0.1 (-0.6 to 0.8) |
| **Tunisia** | 97.9 (78.4-119.2) | 2.09 (1.68-2.52) |  | 372.6 (269.6-509.1) | 2.87 (2.09-3.88) |  | 1.02 (0.89 to 1.16) |
| **Turkey** | 2312.3 (1798.8-2941.9) | 6.97 (5.41-8.74) |  | 7532.2 (5904.6-9102.5) | 8.19 (6.44-9.86) |  | 0.51 (0.26 to 0.76) |
| **Turkmenistan** | 3.4 (2.9-3.8) | 0.18 (0.15-0.20) |  | 115.9 (88.1-155.1) | 2.87 (2.20-3.81) |  | 9.54 (7.04 to 12.1) |
| **Tuvalu** | 0.2 (0.1-0.2) | 2.39 (2.00-2.88) |  | 0.4 (0.3-0.5) | 3.63 (2.80-4.62) |  | 1.35 (1.29 to 1.42) |
| **Uganda** | 162.0 (127.3-204.0) | 2.66 (2.11-3.34) |  | 556.3 (392.9-769.3) | 4.06 (2.92-5.54) |  | 1.36 (1.29 to 1.42) |
| **Ukraine** | 3786.4 (3042.3-4794.3) | 5.25 (4.22-6.65) |  | 5149.7 (3866.4-6549.9) | 6.73 (5.04-8.60) |  | 0.77 (0.05 to 1.49) |
| **United Arab Emirates** | 35.4 (23.0-59.1) | 8.92 (5.88-14.84) |  | 306.8 (240.5-380.8) | 11.40 (9.11-14.14) |  | 0.74 (-1.36 to 2.89) |
| **United Kingdom** | 8059.9 (7680.4-8263.5) | 8.69 (8.30-8.90) |  | 11883.6 (10768.6-12431.8) | 8.62 (7.92-8.97) |  | 0.01 (-0.13 to 0.14) |
| **United Republic of Tanzania** | 252.8 (192.7-336.6) | 2.44 (1.87-3.22) |  | 636.8 (451.8-936.5) | 2.65 (1.92-3.82) |  | 0.28 (0.21 to 0.35) |
| **United States of America** | 29015.6 (27075.4-30096.0) | 8.93 (8.38-9.25) |  | 57098.1 (52181.8-59928.4) | 9.50 (8.74-9.95) |  | 0.17 (-0.04 to 0.38) |
| **United States Virgin Islands** | 3.4 (2.8-4.0) | 4.31 (3.57-5.11) |  | 8.8 (6.5-11.4) | 4.91 (3.67-6.41) |  | 0.33 (-0.26 to 0.94) |
| **Uruguay** | 423.0 (389.9-452.7) | 10.71 (9.89-11.44) |  | 762.4 (688.6-840.7) | 13.27 (12.06-14.54) |  | 0.76 (0.68 to 0.84) |
| **Uzbekistan** | 175.6 (135.5-237.6) | 1.53 (1.17-2.12) |  | 645.2 (507.8-785.2) | 2.48 (1.98-3.02) |  | 1.65 (1.24 to 2.06) |
| **Vanuatu** | 1.1 (0.9-1.5) | 1.99 (1.56-2.51) |  | 4.6 (3.7-5.6) | 2.83 (2.29-3.48) |  | 1.13 (0.99 to 1.27) |
| **Venezuela (Bolivarian Republic of)** | 138.8 (131.2-145.5) | 1.49 (1.40-1.57) |  | 1447.2 (1049.3-1883.1) | 4.91 (3.58-6.38) |  | 3.8 (3.03 to 4.57) |
| **Viet Nam** | 574.0 (459.6-738.5) | 1.46 (1.18-1.88) |  | 2158.3 (1605.5-2658.9) | 2.24 (1.69-2.73) |  | 1.4 (1.3 to 1.51) |
| **Yemen** | 61.6 (35.4-93.4) | 1.33 (0.76-1.97) |  | 254.8 (164.6-352.0) | 1.94 (1.25-2.68) |  | 1.25 (1.1 to 1.4) |
| **Zambia** | 77.2 (64.4-93.1) | 2.85 (2.39-3.41) |  | 219.6 (155.4-296.2) | 3.37 (2.44-4.46) |  | 0.55 (0.36 to 0.73) |
| **Zimbabwe** | 187.9 (150.4-227.8) | 5.11 (4.07-6.16) |  | 507.9 (382.6-647.9) | 7.83 (5.98-9.82) |  | 1.49 (1.07 to 1.91) |

*Abbreviations: ASR, age-standardized rate; AAPC, average annual percentage change; UI, uncertainty interval; CI, confidence interval.*

# Table S8. Case numbers and ASRs of DALYs of pancreatic cancer in 1990 and 2021 for both sexes in 204 countries and AAPC from 1990 to 2021.

| **Location** | **1990** | |  | **2021** | |  | **1990-2021** |
| --- | --- | --- | --- | --- | --- | --- | --- |
|  | **Number**  **(95 % UI)** | **ASR**  **(95 % UI)** |  | **Number**  **(95 % UI)** | **ASR**  **(95 % UI)** |  | **AAPC**  **(95% CI)** |
| **Afghanistan** | 3742.5 (1860.9-7331.8) | 50.94 (26.19-98.10) |  | 8014.9 (4517.6-14520.2) | 72.17 (43.69-126.80) |  | 1.12 (1.07 to 1.18) |
| **Albania** | 2083.4 (1638.5-2605.8) | 97.51 (77.01-120.64) |  | 5087.8 (3581.0-6906.0) | 117.79 (82.88-160.08) |  | 0.54 (0.16 to 0.92) |
| **Algeria** | 3670.1 (2956.0-4575.1) | 29.99 (24.07-36.80) |  | 13971.5 (10832.8-18047.2) | 38.75 (30.24-49.71) |  | 0.84 (0.75 to 0.94) |
| **American Samoa** | 21.0 (17.0-25.3) | 86.47 (70.59-103.94) |  | 63.3 (51.6-78.0) | 125.54 (103.13-153.38) |  | 1.27 (0.99 to 1.55) |
| **Andorra** | 185.1 (127.1-259.4) | 315.81 (218.37-443.89) |  | 348.8 (235.5-468.2) | 228.21 (153.73-306.69) |  | -1.18 (-1.52 to -0.84) |
| **Angola** | 2520.3 (1851.0-3471.6) | 58.22 (43.73-78.68) |  | 8990.6 (5453.9-13219.3) | 68.52 (41.53-99.76) |  | 0.47 (0.39 to 0.55) |
| **Antigua and Barbuda** | 53.9 (49.9-58.1) | 103.68 (95.56-111.40) |  | 136.9 (129.2-144.8) | 125.39 (118.68-132.55) |  | 0.68 (-0.5 to 1.87) |
| **Argentina** | 78779.1 (73781.6-84345.7) | 241.72 (226.27-259.06) |  | 122742.6 (114011.7-131209.6) | 222.58 (207.14-237.67) |  | -0.24 (-0.58 to 0.11) |
| **Armenia** | 5361.5 (4578.8-6354.6) | 186.56 (159.62-221.48) |  | 10645.6 (8816.1-12570.7) | 246.15 (203.97-290.83) |  | 1.01 (0.46 to 1.57) |
| **Australia** | 32261.9 (30752.4-33697.2) | 165.57 (157.85-172.98) |  | 72186.4 (66230.5-77060.2) | 166.21 (154.38-176.95) |  | -0.02 (-0.29 to 0.25) |
| **Austria** | 25107.8 (23885.8-26084.8) | 220.57 (210.13-229.23) |  | 37055.9 (33732.9-39715.3) | 214.64 (198.02-229.22) |  | -0.08 (-0.44 to 0.28) |
| **Azerbaijan** | 3946.5 (2920.0-5550.4) | 73.78 (54.99-103.06) |  | 12250.3 (8376.9-18057.8) | 110.39 (76.55-160.89) |  | 1.3 (1 to 1.61) |
| **Bahamas** | 166.9 (148.0-185.7) | 101.75 (90.11-113.54) |  | 468.0 (379.5-574.1) | 109.57 (89.27-133.90) |  | 0.31 (-0.17 to 0.8) |
| **Bahrain** | 277.6 (239.1-318.4) | 151.48 (129.72-173.61) |  | 1366.8 (1029.6-1915.5) | 142.43 (110.21-194.85) |  | -0.2 (-0.4 to -0.01) |
| **Bangladesh** | 13660.0 (7936.9-18727.1) | 27.43 (15.93-37.32) |  | 41013.9 (27588.9-60618.1) | 28.82 (19.50-42.23) |  | 0.25 (0.01 to 0.49) |
| **Barbados** | 449.7 (408.0-489.9) | 161.90 (147.51-176.17) |  | 852.2 (671.4-1051.8) | 167.81 (132.40-207.21) |  | 0.13 (-0.13 to 0.39) |
| **Belarus** | 18799.1 (17251.2-20357.9) | 143.70 (132.53-155.01) |  | 29059.0 (23530.8-36056.8) | 185.62 (150.67-228.77) |  | 0.78 (0.31 to 1.25) |
| **Belgium** | 29381.9 (27762.8-30784.5) | 195.67 (185.47-204.81) |  | 43324.8 (39340.0-46671.6) | 198.66 (182.59-213.05) |  | -0.06 (-0.85 to 0.74) |
| **Belize** | 80.7 (68.8-103.4) | 85.25 (72.76-108.76) |  | 418.4 (371.2-476.9) | 131.19 (116.40-149.10) |  | 1.46 (0.86 to 2.06) |
| **Benin** | 763.6 (630.2-915.9) | 37.23 (30.87-44.58) |  | 3645.6 (2568.0-4993.4) | 66.35 (47.45-88.92) |  | 1.89 (1.77 to 2.02) |
| **Bermuda** | 155.7 (121.0-179.7) | 248.29 (193.59-286.63) |  | 237.7 (201.9-290.5) | 181.75 (153.47-222.02) |  | -1.16 (-1.77 to -0.54) |
| **Bhutan** | 67.1 (28.2-107.7) | 25.05 (10.61-40.02) |  | 227.6 (125.0-324.5) | 36.80 (20.34-52.08) |  | 1.26 (1.09 to 1.43) |
| **Bolivia (Plurinational State of)** | 4459.7 (2960.4-5758.7) | 133.78 (88.75-172.40) |  | 14683.5 (9810.5-20567.8) | 157.09 (104.72-219.48) |  | 0.53 (0.44 to 0.61) |
| **Bosnia and Herzegovina** | 7453.1 (6350.5-8510.6) | 172.14 (147.16-197.10) |  | 12451.8 (9893.5-15415.3) | 203.30 (160.43-253.60) |  | 0.54 (0.27 to 0.81) |
| **Botswana** | 532.2 (379.6-724.0) | 89.93 (64.84-120.82) |  | 1821.0 (1232.4-2594.9) | 116.72 (81.70-159.61) |  | 0.78 (0.17 to 1.4) |
| **Brazil** | 113826.6 (109410.2-117443.3) | 122.30 (116.98-126.28) |  | 374379.0 (353625.1-390429.7) | 146.98 (138.84-153.46) |  | 0.63 (0.35 to 0.9) |
| **Brunei Darussalam** | 139.2 (109.0-173.5) | 129.34 (101.26-161.43) |  | 486.4 (395.8-588.3) | 130.42 (106.02-156.87) |  | -0.01 (-0.2 to 0.18) |
| **Bulgaria** | 22992.9 (20911.3-25366.0) | 186.28 (170.19-205.21) |  | 34731.2 (29182.2-40642.0) | 262.82 (220.97-308.54) |  | 1.11 (0.44 to 1.78) |
| **Burkina Faso** | 1455.4 (1088.3-1876.8) | 32.17 (24.19-41.24) |  | 5683.3 (3987.5-7851.4) | 58.22 (41.16-80.28) |  | 1.93 (1.63 to 2.22) |
| **Burundi** | 1441.3 (1095.7-1828.9) | 58.47 (44.69-74.02) |  | 2308.0 (1506.0-3591.5) | 42.76 (28.33-65.63) |  | -1.02 (-1.19 to -0.84) |
| **Cabo Verde** | 38.1 (31.5-45.5) | 16.79 (13.91-20.19) |  | 924.0 (689.4-1167.8) | 204.85 (151.66-260.34) |  | 8.55 (7.93 to 9.19) |
| **Cambodia** | 3185.0 (2244.7-4671.2) | 64.29 (45.90-92.31) |  | 11227.8 (8333.7-14648.5) | 84.24 (63.37-108.40) |  | 0.89 (0.8 to 0.99) |
| **Cameroon** | 2596.2 (2037.7-3273.2) | 55.01 (43.52-68.92) |  | 13268.0 (8916.5-18632.3) | 96.52 (66.29-134.84) |  | 1.83 (1.75 to 1.9) |
| **Canada** | 62976.8 (59535.9-65925.3) | 195.43 (184.80-204.79) |  | 116504.5 (105824.7-125100.5) | 166.92 (153.33-179.03) |  | -0.6 (-1.02 to -0.19) |
| **Central African Republic** | 800.4 (541.7-1026.6) | 62.84 (44.89-79.05) |  | 1490.0 (1057.4-2093.7) | 58.07 (42.57-79.55) |  | -0.26 (-0.41 to -0.12) |
| **Chad** | 704.1 (531.2-906.6) | 24.25 (18.33-31.23) |  | 3242.2 (2453.9-4172.4) | 51.97 (39.45-66.38) |  | 2.53 (2.43 to 2.62) |
| **Chile** | 13511.2 (12745.3-14230.1) | 133.57 (125.82-140.68) |  | 38145.0 (35459.7-40987.6) | 149.04 (138.47-159.96) |  | 0.32 (-0.17 to 0.81) |
| **China** | 1120352.9 (941075.6-1306508.7) | 123.16 (103.69-143.27) |  | 2930317.0 (2301048.7-3575078.9) | 137.23 (108.15-166.74) |  | 0.36 (0.18 to 0.53) |
| **Colombia** | 23884.8 (22648.7-24953.4) | 129.06 (122.32-134.73) |  | 58320.6 (48337.6-69145.9) | 105.63 (87.55-125.02) |  | -0.7 (-1.37 to -0.02) |
| **Comoros** | 143.7 (105.0-188.4) | 67.29 (50.53-88.54) |  | 364.1 (251.7-518.1) | 70.42 (49.16-99.21) |  | 0.13 (-0.08 to 0.35) |
| **Congo** | 1085.9 (692.2-1488.2) | 94.77 (62.24-127.71) |  | 2844.8 (1875.7-4103.6) | 93.76 (62.69-135.01) |  | 0.01 (-0.26 to 0.29) |
| **Cook Islands** | 11.5 (9.7-13.7) | 88.80 (75.03-105.09) |  | 24.2 (19.1-30.1) | 93.11 (73.33-115.53) |  | 0.18 (0.06 to 0.29) |
| **Costa Rica** | 1723.5 (1599.4-1850.9) | 96.38 (89.18-103.69) |  | 7190.9 (6331.9-8151.7) | 130.45 (114.86-147.77) |  | 0.93 (0.23 to 1.63) |
| **Coted'Ivoire** | 1498.4 (1158.1-1938.4) | 34.34 (27.15-43.41) |  | 5345.4 (3669.4-7731.3) | 43.64 (30.77-61.49) |  | 0.78 (0.59 to 0.96) |
| **Croatia** | 12471.6 (11346.5-13487.9) | 202.00 (183.48-217.89) |  | 17031.3 (14743.4-19552.3) | 198.27 (172.09-226.44) |  | -0.01 (-1.05 to 1.04) |
| **Cuba** | 13689.3 (12875.4-14569.1) | 132.24 (124.15-140.74) |  | 23702.4 (20232.9-27535.6) | 123.85 (105.64-143.88) |  | -0.25 (-0.81 to 0.32) |
| **Cyprus** | 996.4 (789.6-1217.0) | 129.35 (102.61-159.03) |  | 2901.7 (2270.9-3588.5) | 142.46 (112.16-176.10) |  | 0.29 (0.05 to 0.53) |
| **Czechia** | 39220.6 (36394.8-42546.3) | 287.54 (266.66-312.56) |  | 54379.3 (46541.0-62613.4) | 263.92 (225.81-302.30) |  | -0.25 (-0.36 to -0.14) |
| **Democratic People's Republic of Korea** | 16717.0 (11730.1-23329.5) | 93.62 (66.55-128.98) |  | 33123.9 (21164.4-45864.0) | 96.83 (61.86-132.78) |  | 0.1 (0.06 to 0.14) |
| **Democratic Republic of the Congo** | 9020.4 (7068.1-11229.9) | 53.38 (42.13-66.01) |  | 20687.6 (12656.4-30561.7) | 51.17 (31.36-76.16) |  | -0.16 (-0.28 to -0.03) |
| **Denmark** | 12251.9 (11249.4-13556.9) | 160.19 (147.80-176.16) |  | 24634.7 (22524.8-26479.7) | 212.72 (196.55-228.18) |  | 0.99 (0.36 to 1.62) |
| **Djibouti** | 88.9 (53.6-128.2) | 58.07 (35.43-81.50) |  | 505.5 (305.6-772.4) | 71.09 (43.90-105.97) |  | 0.65 (0.55 to 0.74) |
| **Dominica** | 81.9 (67.3-103.3) | 138.85 (114.64-173.20) |  | 162.6 (127.7-207.2) | 192.00 (152.05-243.59) |  | 1.06 (0.97 to 1.14) |
| **Dominican Republic** | 3111.6 (2522.3-3860.8) | 79.52 (64.56-98.70) |  | 14023.7 (11036.4-17854.8) | 137.41 (108.24-175.55) |  | 1.88 (1.45 to 2.31) |
| **Ecuador** | 4533.6 (4210.5-4861.6) | 84.26 (78.09-90.63) |  | 17524.0 (13478.7-22079.2) | 106.17 (81.87-133.52) |  | 0.64 (-0.05 to 1.34) |
| **Egypt** | 16606.1 (14585.3-19041.1) | 55.31 (48.71-63.19) |  | 90795.4 (73689.1-110571.8) | 132.62 (109.26-160.95) |  | 2.86 (2.65 to 3.08) |
| **El Salvador** | 1950.8 (1777.4-2145.0) | 63.14 (57.26-69.56) |  | 6865.7 (5494.1-8463.3) | 112.94 (90.02-139.15) |  | 1.99 (1.62 to 2.36) |
| **Equatorial Guinea** | 132.8 (91.8-175.6) | 63.07 (45.18-81.31) |  | 627.9 (364.6-941.2) | 111.35 (65.73-162.12) |  | 1.87 (1.53 to 2.21) |
| **Eritrea** | 752.8 (576.1-953.6) | 57.12 (44.32-71.44) |  | 1895.0 (1137.9-2709.9) | 60.93 (38.05-85.74) |  | 0.18 (0.06 to 0.31) |
| **Estonia** | 4345.6 (3975.6-4772.4) | 213.01 (194.40-234.62) |  | 5769.3 (4880.6-6549.8) | 229.88 (195.03-262.13) |  | 0.21 (-0.21 to 0.63) |
| **Eswatini** | 363.6 (244.9-533.2) | 118.48 (80.45-172.92) |  | 1187.5 (701.9-1816.1) | 191.34 (116.43-288.51) |  | 1.58 (1.36 to 1.8) |
| **Ethiopia** | 6005.8 (2813.0-9376.2) | 27.56 (12.77-42.98) |  | 12676.6 (7569.1-19767.1) | 27.27 (16.29-42.95) |  | -0.03 (-0.1 to 0.05) |
| **Fiji** | 236.3 (193.2-283.0) | 59.91 (48.92-71.85) |  | 647.3 (472.8-854.7) | 79.37 (58.60-103.81) |  | 0.91 (0.81 to 1.01) |
| **Finland** | 16554.4 (15643.3-17370.6) | 235.80 (223.23-247.19) |  | 28028.4 (25251.5-30380.9) | 233.76 (214.21-252.60) |  | 0 (-0.48 to 0.48) |
| **France** | 134614.3 (125968.7-146102.2) | 172.30 (161.97-186.04) |  | 268044.4 (241742.9-295720.0) | 208.69 (190.88-229.36) |  | 0.62 (0.42 to 0.82) |
| **Gabon** | 566.4 (384.3-867.7) | 96.30 (65.05-147.17) |  | 1468.2 (1024.9-1986.4) | 130.40 (92.18-173.99) |  | 0.99 (0.82 to 1.15) |
| **Gambia** | 126.2 (95.3-165.0) | 33.64 (25.64-43.20) |  | 529.7 (371.3-721.7) | 50.76 (35.50-68.55) |  | 1.39 (0.83 to 1.94) |
| **Georgia** | 3510.5 (2846.3-4345.7) | 53.89 (43.89-66.52) |  | 9833.3 (8493.2-11247.4) | 173.74 (149.69-198.81) |  | 4.13 (2.58 to 5.7) |
| **Germany** | 242930.4 (227459.6-256576.8) | 197.01 (184.76-208.08) |  | 411634.9 (376654.0-441155.8) | 228.68 (211.05-242.62) |  | 0.45 (0.11 to 0.8) |
| **Ghana** | 2419.2 (1646.5-3407.0) | 35.86 (24.53-50.78) |  | 17562.5 (12575.4-24639.6) | 98.23 (70.33-138.61) |  | 3.32 (3.13 to 3.51) |
| **Greece** | 31153.5 (29454.3-32536.7) | 205.68 (194.75-214.53) |  | 48759.5 (45012.2-51337.4) | 226.75 (211.88-238.22) |  | 0.28 (-0.05 to 0.62) |
| **Greenland** | 180.2 (152.6-208.1) | 489.10 (415.27-562.62) |  | 279.6 (226.0-338.9) | 374.93 (306.07-455.02) |  | -0.81 (-1.11 to -0.52) |
| **Grenada** | 114.0 (100.6-129.2) | 167.66 (146.75-191.42) |  | 265.7 (231.4-301.0) | 226.07 (197.19-254.87) |  | 1 (0.59 to 1.4) |
| **Guam** | 70.2 (61.3-79.2) | 87.91 (76.40-100.44) |  | 224.6 (192.7-259.9) | 108.47 (93.48-125.12) |  | 0.62 (-0.25 to 1.5) |
| **Guatemala** | 1848.1 (1771.1-1927.9) | 48.73 (46.58-50.91) |  | 9228.9 (7898.4-10746.4) | 81.14 (69.62-94.31) |  | 1.4 (0.31 to 2.5) |
| **Guinea** | 794.6 (620.1-1021.1) | 23.26 (18.17-29.75) |  | 2267.4 (1666.8-3111.1) | 37.62 (28.01-51.12) |  | 1.57 (1.45 to 1.69) |
| **Guinea-Bissau** | 213.4 (137.0-289.2) | 49.79 (32.33-67.09) |  | 662.8 (464.4-895.5) | 81.26 (57.21-108.59) |  | 1.6 (1.53 to 1.68) |
| **Guyana** | 365.1 (325.2-412.3) | 90.64 (80.80-102.30) |  | 897.8 (683.8-1158.4) | 130.72 (100.49-166.98) |  | 1.35 (0.69 to 2.01) |
| **Haiti** | 3473.5 (2241.8-4555.6) | 100.80 (67.41-130.92) |  | 7610.6 (5142.2-10612.0) | 97.40 (66.31-133.47) |  | -0.06 (-0.13 to 0.02) |
| **Honduras** | 1513.8 (1263.5-1793.9) | 68.94 (57.56-81.61) |  | 8065.4 (5903.4-11342.1) | 122.23 (89.39-171.53) |  | 1.89 (1.54 to 2.24) |
| **Hungary** | 36397.4 (33293.2-39453.4) | 252.25 (230.71-273.04) |  | 48218.3 (41437.2-55080.9) | 263.17 (226.67-300.75) |  | 0.13 (-0.4 to 0.67) |
| **Iceland** | 567.1 (529.3-601.8) | 202.87 (189.89-216.01) |  | 1107.8 (978.1-1228.0) | 197.45 (175.57-218.46) |  | -0.09 (-0.69 to 0.52) |
| **India** | 138579.8 (110346.0-168061.9) | 27.34 (21.77-33.23) |  | 464574.9 (405698.9-522935.6) | 37.37 (32.60-41.99) |  | 1.02 (0.88 to 1.16) |
| **Indonesia** | 64703.0 (50554.4-78965.1) | 58.94 (45.49-72.04) |  | 249025.4 (181624.1-319398.0) | 95.32 (68.76-122.09) |  | 1.57 (1.5 to 1.63) |
| **Iran (Islamic Republic of)** | 13588.9 (11145.2-15922.1) | 49.18 (40.29-57.49) |  | 66294.1 (58493.7-73345.9) | 82.59 (72.75-91.36) |  | 1.65 (1.4 to 1.91) |
| **Iraq** | 6596.7 (4525.2-8956.2) | 78.72 (53.77-106.62) |  | 29946.9 (21880.6-38863.2) | 116.13 (84.97-149.01) |  | 1.28 (1.1 to 1.47) |
| **Ireland** | 8836.3 (8363.6-9227.6) | 218.89 (207.34-228.77) |  | 12617.8 (11451.8-13883.2) | 162.51 (147.66-178.38) |  | -0.93 (-1.46 to -0.4) |
| **Israel** | 9986.6 (9415.0-10522.9) | 208.18 (195.75-218.89) |  | 23600.9 (21130.3-25423.8) | 196.05 (177.08-210.45) |  | -0.15 (-0.68 to 0.4) |
| **Italy** | 180899.8 (173087.3-186533.5) | 208.53 (200.08-214.88) |  | 267042.6 (239836.8-285248.6) | 197.65 (181.55-209.36) |  | -0.24 (-0.52 to 0.05) |
| **Jamaica** | 1444.3 (1294.4-1674.6) | 82.36 (74.01-95.47) |  | 3184.8 (2440.2-4112.3) | 103.32 (79.16-133.36) |  | 1.04 (-0.76 to 2.88) |
| **Japan** | 338688.0 (323968.3-347693.5) | 197.54 (188.57-202.98) |  | 709065.4 (625626.7-755943.9) | 215.31 (196.04-225.71) |  | 0.31 (0.2 to 0.42) |
| **Jordan** | 903.3 (738.8-1091.9) | 61.47 (50.52-73.93) |  | 6263.8 (4512.0-8678.5) | 77.16 (55.97-105.52) |  | 0.77 (0.36 to 1.19) |
| **Kazakhstan** | 18050.7 (14446.8-23830.0) | 135.29 (108.39-177.64) |  | 27751.5 (23162.8-32397.7) | 144.25 (121.00-167.99) |  | 0.24 (-0.31 to 0.8) |
| **Kenya** | 3392.0 (2170.7-4801.2) | 38.62 (24.75-54.67) |  | 18441.1 (14422.8-24298.4) | 73.99 (57.94-96.74) |  | 2.1 (1.92 to 2.28) |
| **Kiribati** | 9.5 (7.7-11.5) | 24.35 (19.59-29.54) |  | 23.0 (16.1-31.5) | 29.95 (21.31-40.61) |  | 0.69 (0.63 to 0.75) |
| **Kuwait** | 502.2 (458.9-546.9) | 74.70 (67.85-80.83) |  | 3180.4 (2639.5-3861.0) | 95.48 (78.44-116.17) |  | 0.78 (-1.22 to 2.82) |
| **Kyrgyzstan** | 3190.4 (2677.6-3718.5) | 103.48 (87.04-120.46) |  | 7033.1 (5749.7-8602.7) | 132.86 (108.87-161.67) |  | 0.81 (-0.48 to 2.12) |
| **Lao People's Democratic Republic** | 1466.0 (840.7-2335.8) | 64.58 (37.52-102.56) |  | 3832.8 (2737.5-5236.3) | 75.57 (54.34-102.59) |  | 0.52 (0.47 to 0.57) |
| **Latvia** | 7483.7 (6833.5-8168.2) | 209.49 (191.12-228.46) |  | 9328.1 (7977.5-10836.4) | 260.71 (222.62-303.61) |  | 0.68 (0.19 to 1.18) |
| **Lebanon** | 1939.3 (1291.3-2550.7) | 86.32 (58.62-112.51) |  | 5785.8 (4225.1-7806.1) | 97.89 (71.51-131.73) |  | 0.43 (0.14 to 0.72) |
| **Lesotho** | 552.4 (415.4-746.0) | 63.76 (48.06-85.90) |  | 1730.1 (1111.0-2506.2) | 150.99 (97.33-217.95) |  | 2.88 (2.63 to 3.13) |
| **Liberia** | 477.3 (342.6-642.6) | 40.02 (28.94-53.17) |  | 1520.8 (842.0-2342.9) | 64.78 (36.21-99.19) |  | 1.52 (1.24 to 1.81) |
| **Libya** | 2574.2 (1816.3-3418.1) | 130.19 (92.63-171.86) |  | 10667.5 (7526.2-14402.1) | 186.50 (130.07-247.52) |  | 1.15 (0.9 to 1.4) |
| **Lithuania** | 9614.2 (8779.8-10402.2) | 213.61 (195.21-230.64) |  | 12855.9 (11146.7-14460.8) | 247.30 (213.72-277.98) |  | 0.56 (-0.19 to 1.31) |
| **Luxembourg** | 1171.8 (1109.6-1229.2) | 219.20 (207.72-229.47) |  | 1920.8 (1722.2-2113.7) | 184.20 (165.20-202.79) |  | -0.61 (-1.06 to -0.15) |
| **Madagascar** | 2643.1 (1950.3-3285.0) | 48.39 (35.68-60.27) |  | 5560.4 (3743.4-7970.6) | 43.43 (29.63-61.26) |  | -0.39 (-0.6 to -0.17) |
| **Malawi** | 819.1 (633.4-1013.5) | 19.73 (15.37-24.56) |  | 2080.9 (1454.0-2914.7) | 25.84 (18.40-35.68) |  | 0.9 (0.74 to 1.06) |
| **Malaysia** | 4332.0 (3630.9-5050.3) | 44.54 (37.20-51.79) |  | 19175.7 (16529.4-22239.7) | 65.31 (56.16-75.53) |  | 1.35 (1.07 to 1.63) |
| **Maldives** | 51.5 (31.1-75.2) | 52.39 (33.61-75.27) |  | 151.6 (116.5-193.2) | 41.55 (32.17-51.69) |  | -0.81 (-1.01 to -0.61) |
| **Mali** | 2096.4 (1701.2-2515.1) | 49.51 (40.29-59.11) |  | 5910.4 (4297.2-7961.9) | 62.51 (46.08-82.97) |  | 0.79 (0.65 to 0.92) |
| **Malta** | 875.2 (815.8-936.5) | 203.36 (189.03-217.87) |  | 1883.6 (1687.0-2108.0) | 209.45 (188.59-233.49) |  | 0.09 (-0.18 to 0.36) |
| **Marshall Islands** | 10.7 (8.8-13.0) | 62.00 (50.98-75.65) |  | 36.6 (26.0-50.7) | 93.07 (67.57-127.19) |  | 1.33 (1.24 to 1.43) |
| **Mauritania** | 515.3 (397.8-636.6) | 49.74 (38.09-61.40) |  | 1953.1 (1415.8-2615.0) | 87.39 (63.21-116.56) |  | 1.84 (1.63 to 2.06) |
| **Mauritius** | 896.9 (851.1-942.4) | 116.35 (110.16-122.02) |  | 2400.7 (2206.0-2542.5) | 129.08 (118.83-135.92) |  | 0.34 (-0.88 to 1.56) |
| **Mexico** | 59473.6 (58251.0-60630.3) | 134.45 (131.37-137.32) |  | 157206.6 (138594.5-176605.7) | 120.88 (106.66-135.61) |  | -0.37 (-0.71 to -0.03) |
| **Micronesia (Federated States of)** | 38.5 (30.4-50.2) | 75.98 (60.34-98.76) |  | 86.0 (62.2-117.2) | 106.61 (77.36-143.25) |  | 1.1 (1.07 to 1.13) |
| **Monaco** | 167.0 (120.2-226.3) | 262.15 (189.57-351.82) |  | 261.1 (172.6-378.0) | 290.87 (189.90-425.59) |  | 0.33 (0.27 to 0.39) |
| **Mongolia** | 423.7 (322.4-553.6) | 38.61 (29.32-50.70) |  | 5188.6 (3889.4-6826.8) | 198.98 (149.16-263.83) |  | 5.34 (4.66 to 6.03) |
| **Montenegro** | 1222.8 (1002.4-1489.5) | 190.58 (155.62-232.50) |  | 2304.2 (1812.9-2850.2) | 236.01 (186.01-290.85) |  | 0.75 (0.44 to 1.06) |
| **Morocco** | 3834.7 (3137.3-4677.0) | 26.03 (21.26-31.77) |  | 15329.1 (11127.3-18883.1) | 42.85 (31.27-53.00) |  | 1.63 (1.54 to 1.71) |
| **Mozambique** | 737.5 (605.2-891.9) | 12.60 (10.53-15.19) |  | 2131.5 (1571.2-2817.6) | 19.30 (14.37-25.11) |  | 1.41 (1.28 to 1.54) |
| **Myanmar** | 15847.7 (10158.6-23965.9) | 62.81 (41.17-94.87) |  | 38887.9 (28316.4-52321.3) | 75.71 (55.63-100.19) |  | 0.61 (0.56 to 0.65) |
| **Namibia** | 195.6 (154.7-246.4) | 28.03 (22.18-34.96) |  | 584.2 (405.7-786.8) | 38.85 (27.24-51.40) |  | 1.09 (0.9 to 1.27) |
| **Nauru** | 6.0 (3.7-9.3) | 117.06 (75.43-180.44) |  | 9.2 (5.5-13.0) | 144.42 (88.41-201.04) |  | 0.68 (0.6 to 0.77) |
| **Nepal** | 2242.1 (1197.6-3368.4) | 22.23 (11.64-33.15) |  | 7724.1 (4602.7-11674.4) | 32.03 (19.12-48.25) |  | 1.19 (1.05 to 1.34) |
| **Netherlands** | 38731.1 (36734.3-40461.7) | 198.35 (188.44-206.91) |  | 63156.0 (57700.1-67525.4) | 186.42 (171.54-199.49) |  | -0.23 (-0.57 to 0.12) |
| **New Zealand** | 6265.0 (5863.3-6644.9) | 160.66 (150.63-170.47) |  | 12876.8 (11752.9-13843.4) | 157.68 (144.68-169.39) |  | -0.1 (-0.64 to 0.43) |
| **Nicaragua** | 785.7 (682.2-904.4) | 47.66 (41.49-54.95) |  | 3759.4 (2989.4-4655.7) | 74.19 (58.96-91.60) |  | 1.47 (1.15 to 1.79) |
| **Niger** | 812.1 (564.0-1062.3) | 27.18 (19.21-35.12) |  | 3581.3 (2203.2-5228.0) | 41.05 (26.07-59.04) |  | 1.37 (1.22 to 1.53) |
| **Nigeria** | 6835.3 (4594.3-8689.7) | 15.07 (10.23-19.04) |  | 24053.3 (17819.5-32148.9) | 25.25 (19.07-32.88) |  | 1.67 (1.59 to 1.74) |
| **Niue** | 1.6 (1.2-2.1) | 74.90 (54.31-95.66) |  | 2.6 (2.0-3.4) | 118.87 (90.22-155.97) |  | 1.51 (1.43 to 1.6) |
| **North Macedonia** | 3212.1 (2670.6-3852.3) | 165.71 (137.78-198.37) |  | 6782.3 (5121.4-8874.0) | 201.15 (152.68-262.16) |  | 0.62 (0.42 to 0.83) |
| **Northern Mariana Islands** | 18.3 (14.2-23.7) | 89.63 (71.94-112.85) |  | 95.3 (79.3-111.1) | 173.54 (145.21-203.11) |  | 2.11 (1.8 to 2.43) |
| **Norway** | 12673.2 (12075.1-13097.1) | 195.83 (187.16-202.06) |  | 18523.7 (17075.6-19478.7) | 190.47 (176.65-199.61) |  | -0.06 (-0.3 to 0.18) |
| **Oman** | 235.2 (166.8-329.9) | 32.36 (22.99-45.24) |  | 983.6 (701.6-1348.1) | 44.15 (32.00-56.73) |  | 1.03 (0.61 to 1.45) |
| **Pakistan** | 13891.3 (11554.3-16523.0) | 23.65 (19.73-27.98) |  | 47648.1 (36765.7-63676.7) | 36.11 (27.98-48.43) |  | 1.37 (1.25 to 1.49) |
| **Palau** | 19.1 (14.4-24.8) | 195.20 (148.11-249.80) |  | 44.6 (34.4-56.6) | 209.62 (164.45-260.78) |  | 0.23 (0.08 to 0.37) |
| **Palestine** | 965.6 (668.1-1352.2) | 109.26 (76.25-151.68) |  | 3306.7 (2728.7-3917.0) | 123.39 (102.06-145.68) |  | 0.37 (0.2 to 0.54) |
| **Panama** | 820.3 (770.9-872.3) | 53.35 (50.06-56.79) |  | 3805.3 (2997.6-4539.2) | 86.31 (67.99-103.02) |  | 1.55 (0.78 to 2.33) |
| **Papua New Guinea** | 726.8 (480.8-1097.8) | 36.88 (24.86-55.62) |  | 2608.2 (1820.7-3882.6) | 46.58 (32.26-68.97) |  | 0.77 (0.75 to 0.8) |
| **Paraguay** | 1281.4 (1078.4-1543.0) | 56.19 (47.29-67.42) |  | 7596.9 (5711.1-9977.1) | 128.35 (96.44-168.30) |  | 2.84 (2.62 to 3.07) |
| **Peru** | 14375.4 (11636.6-17940.6) | 116.33 (95.11-145.36) |  | 42491.7 (30997.3-57105.7) | 126.07 (92.16-169.27) |  | 0.35 (-0.48 to 1.18) |
| **Philippines** | 20454.8 (17960.1-23683.3) | 60.54 (53.00-71.28) |  | 78994.7 (65347.4-94198.1) | 88.40 (73.43-105.65) |  | 1.27 (1.12 to 1.42) |
| **Poland** | 101201.1 (97449.6-104404.7) | 230.97 (222.43-238.31) |  | 146784.6 (133801.4-159033.2) | 212.27 (193.27-230.02) |  | -0.37 (-0.45 to -0.28) |
| **Portugal** | 22086.8 (20877.6-23165.2) | 162.02 (153.02-169.96) |  | 37215.4 (33872.9-40006.8) | 165.82 (152.68-178.02) |  | 0.05 (-0.21 to 0.31) |
| **Puerto Rico** | 3554.9 (3323.7-3773.0) | 97.66 (91.42-103.59) |  | 9409.9 (7742.1-11133.1) | 148.90 (121.71-176.61) |  | 1.43 (0.48 to 2.38) |
| **Qatar** | 181.3 (144.6-226.1) | 148.02 (121.09-179.59) |  | 1456.8 (1036.8-2124.3) | 133.17 (95.85-190.57) |  | -0.46 (-0.95 to 0.03) |
| **Republic of Korea** | 65347.3 (55924.6-75064.4) | 204.18 (174.62-234.83) |  | 146128.9 (116596.0-177361.0) | 155.42 (124.26-188.67) |  | -0.92 (-1.04 to -0.79) |
| **Republic of Moldova** | 8958.7 (8309.4-9659.9) | 196.75 (182.36-212.49) |  | 12178.4 (10782.5-13615.6) | 207.25 (183.99-231.49) |  | 0.04 (-0.47 to 0.56) |
| **Romania** | 47118.4 (43475.7-50850.9) | 165.09 (152.41-178.06) |  | 83300.6 (72056.7-94875.9) | 242.58 (209.98-276.65) |  | 1.26 (0.76 to 1.76) |
| **Russian Federation** | 402056.6 (393092.7-410881.4) | 219.58 (214.62-224.64) |  | 520896.3 (475672.6-563227.9) | 222.07 (202.76-240.13) |  | 0.16 (-0.37 to 0.69) |
| **Rwanda** | 2423.2 (1859.9-2970.0) | 78.31 (61.02-95.47) |  | 4282.1 (2859.9-6426.0) | 63.16 (42.74-93.77) |  | -0.68 (-0.8 to -0.56) |
| **Saint Kitts and Nevis** | 57.3 (47.1-66.1) | 158.55 (129.48-182.27) |  | 117.8 (95.5-139.2) | 164.78 (134.93-193.50) |  | 0.3 (0.07 to 0.54) |
| **Saint Lucia** | 117.7 (104.1-130.2) | 136.12 (120.54-150.45) |  | 362.1 (294.7-437.3) | 149.91 (122.30-180.98) |  | 0.26 (-0.24 to 0.77) |
| **Saint Vincent and the Grenadines** | 93.4 (85.3-102.1) | 131.06 (119.86-143.07) |  | 206.9 (180.8-235.5) | 143.87 (125.91-163.57) |  | 0.29 (-0.21 to 0.81) |
| **Samoa** | 71.4 (57.1-85.9) | 81.28 (65.47-96.81) |  | 135.3 (105.1-172.7) | 90.06 (70.65-113.90) |  | 0.33 (0.24 to 0.41) |
| **San Marino** | 66.0 (55.0-77.6) | 191.61 (159.71-224.07) |  | 84.7 (54.5-121.9) | 123.34 (76.80-178.64) |  | -1.56 (-1.91 to -1.21) |
| **Sao Tome and Principe** | 9.7 (7.8-11.5) | 14.67 (11.88-17.33) |  | 28.2 (21.4-39.2) | 23.67 (18.19-32.43) |  | 1.57 (1.35 to 1.78) |
| **Saudi Arabia** | 2629.8 (1962.5-3431.3) | 41.07 (31.13-52.95) |  | 17903.7 (13746.3-23060.6) | 74.67 (59.69-94.32) |  | 1.94 (1.86 to 2.01) |
| **Senegal** | 1335.3 (1081.9-1630.9) | 39.41 (32.15-48.14) |  | 6065.3 (4522.0-8131.7) | 74.49 (56.18-97.98) |  | 2.08 (1.69 to 2.47) |
| **Serbia** | 22061.8 (17609.9-28159.6) | 193.24 (154.01-244.57) |  | 33645.7 (26159.4-41665.8) | 210.71 (162.33-262.94) |  | 0.33 (-0.1 to 0.77) |
| **Seychelles** | 74.5 (64.2-85.1) | 132.24 (113.52-151.38) |  | 162.8 (134.8-194.5) | 133.12 (110.62-158.60) |  | -0.01 (-0.19 to 0.17) |
| **Sierra Leone** | 652.4 (501.5-822.4) | 31.03 (24.07-38.82) |  | 2193.1 (1555.6-2969.2) | 54.42 (38.98-73.03) |  | 1.84 (1.74 to 1.94) |
| **Singapore** | 2871.0 (2709.1-3036.4) | 123.74 (116.65-130.78) |  | 9262.1 (8548.3-9957.7) | 106.62 (98.29-114.43) |  | -0.26 (-0.46 to -0.06) |
| **Slovakia** | 14457.1 (12068.1-17696.8) | 242.25 (200.98-297.16) |  | 21877.5 (17166.6-27675.0) | 234.45 (182.90-297.93) |  | -0.14 (-0.54 to 0.26) |
| **Slovenia** | 4908.7 (4579.6-5249.2) | 199.52 (186.37-213.06) |  | 7793.2 (6611.2-8952.9) | 183.85 (156.60-210.45) |  | -0.41 (-1.08 to 0.28) |
| **Solomon Islands** | 72.7 (42.0-101.8) | 48.83 (30.20-67.52) |  | 278.3 (201.3-378.8) | 71.35 (52.30-96.68) |  | 1.22 (0.97 to 1.46) |
| **Somalia** | 1259.9 (750.0-2033.6) | 45.95 (27.57-73.48) |  | 2576.5 (1309.6-4302.7) | 36.62 (18.70-60.82) |  | -0.71 (-0.8 to -0.63) |
| **South Africa** | 21948.3 (18625.0-27512.8) | 99.95 (84.09-127.63) |  | 69539.8 (60852.0-77636.4) | 143.79 (126.03-160.48) |  | 1.14 (0.7 to 1.58) |
| **South Sudan** | 1617.6 (1020.1-2484.0) | 60.04 (37.97-92.40) |  | 2718.2 (1579.5-3953.9) | 63.07 (37.43-91.59) |  | 0.2 (0.09 to 0.3) |
| **Spain** | 85452.7 (80735.2-89758.4) | 161.29 (152.70-169.16) |  | 152767.8 (136455.0-163810.8) | 170.74 (155.30-182.51) |  | 0.14 (-0.05 to 0.34) |
| **Sri Lanka** | 3995.6 (3473.9-4556.0) | 34.66 (30.15-39.69) |  | 9884.3 (6359.6-13993.4) | 35.75 (23.07-50.63) |  | 0.21 (-0.35 to 0.77) |
| **Sudan** | 3422.6 (2213.1-5299.3) | 34.79 (22.56-53.47) |  | 13149.5 (8264.5-20565.2) | 61.43 (39.54-95.28) |  | 1.87 (1.79 to 1.94) |
| **Suriname** | 328.3 (274.0-383.4) | 123.67 (103.76-144.58) |  | 977.8 (735.9-1263.0) | 149.80 (113.27-193.41) |  | 0.67 (0.26 to 1.09) |
| **Sweden** | 31744.7 (29794.4-33416.0) | 223.22 (210.72-233.27) |  | 39706.5 (34660.2-44354.4) | 191.25 (169.05-213.19) |  | -0.53 (-1.03 to -0.02) |
| **Switzerland** | 13974.6 (12875.1-15238.8) | 140.54 (130.01-152.40) |  | 28689.1 (25813.5-31828.8) | 164.08 (149.08-181.07) |  | 0.38 (-0.25 to 1.03) |
| **Syrian Arab Republic** | 3413.9 (2535.3-4369.1) | 60.62 (45.06-77.37) |  | 12370.3 (8727.2-17042.6) | 87.64 (62.49-119.07) |  | 1.16 (0.87 to 1.45) |
| **Taiwan (Province of China)** | 18573.8 (17626.7-19379.4) | 108.68 (102.99-113.79) |  | 69552.0 (63195.5-74401.6) | 167.99 (152.47-179.37) |  | 1.36 (0.88 to 1.85) |
| **Tajikistan** | 1269.6 (874.4-1821.6) | 44.34 (30.32-63.31) |  | 2459.1 (1704.1-3505.9) | 38.22 (26.63-54.28) |  | -0.52 (-0.9 to -0.14) |
| **Thailand** | 35169.7 (29684.8-42230.8) | 91.87 (77.50-109.30) |  | 127012.1 (97429.5-165950.8) | 118.81 (91.59-154.14) |  | 0.83 (0.64 to 1.03) |
| **Timor-Leste** | 132.8 (79.3-197.2) | 41.09 (24.61-59.54) |  | 475.8 (366.2-609.3) | 53.17 (41.02-67.73) |  | 0.86 (0.74 to 0.98) |
| **Togo** | 464.5 (349.0-598.9) | 34.96 (26.49-44.52) |  | 2901.1 (1975.7-3934.7) | 69.46 (47.18-94.83) |  | 2.26 (2.17 to 2.35) |
| **Tokelau** | 0.8 (0.6-1.2) | 61.91 (42.85-85.22) |  | 1.3 (0.9-1.8) | 89.05 (62.21-120.23) |  | 1.2 (1.15 to 1.25) |
| **Tonga** | 58.5 (46.1-74.4) | 102.20 (80.53-130.09) |  | 110.6 (82.9-142.7) | 135.51 (101.55-174.38) |  | 0.9 (0.64 to 1.16) |
| **Trinidad and Tobago** | 1083.7 (1017.6-1158.3) | 127.06 (119.03-135.95) |  | 2529.6 (1891.4-3235.7) | 130.53 (97.75-167.03) |  | 0.2 (-0.22 to 0.63) |
| **Tunisia** | 2455.7 (1974.0-2971.8) | 47.46 (38.23-57.53) |  | 8941.5 (6451.1-12436.3) | 65.59 (47.20-90.89) |  | 1.04 (0.92 to 1.16) |
| **Turkey** | 62638.0 (48312.5-80804.1) | 170.28 (131.89-218.36) |  | 179791.0 (139606.4-219876.3) | 187.05 (145.34-227.76) |  | 0.29 (0.03 to 0.55) |
| **Turkmenistan** | 97.1 (85.1-109.6) | 4.68 (4.09-5.28) |  | 3376.6 (2536.7-4537.3) | 75.86 (57.40-101.51) |  | 9.55 (6.99 to 12.17) |
| **Tuvalu** | 4.2 (3.3-5.1) | 58.78 (48.63-71.33) |  | 9.5 (7.4-12.4) | 88.05 (68.64-113.78) |  | 1.32 (1.28 to 1.37) |
| **Uganda** | 4332.2 (3355.6-5559.5) | 63.87 (50.04-81.22) |  | 15499.8 (10661.8-21886.1) | 96.08 (67.46-133.03) |  | 1.33 (1.23 to 1.43) |
| **Ukraine** | 101509.3 (80906.3-132061.0) | 142.83 (113.67-187.03) |  | 133708.9 (98385.7-171299.9) | 184.35 (135.33-238.20) |  | 0.88 (0.13 to 1.64) |
| **United Arab Emirates** | 1126.9 (727.3-1897.4) | 213.52 (137.50-355.05) |  | 9946.7 (7691.4-12473.9) | 231.98 (186.54-287.96) |  | 0.08 (-1.47 to 1.65) |
| **United Kingdom** | 167428.0 (161951.6-170676.6) | 193.11 (187.34-196.60) |  | 223758.6 (209238.1-231688.6) | 180.17 (170.63-185.95) |  | -0.22 (-0.37 to -0.06) |
| **United Republic of Tanzania** | 6878.2 (5229.9-9287.6) | 59.29 (45.17-79.20) |  | 17289.0 (11988.7-26336.7) | 62.85 (44.20-93.61) |  | 0.19 (0.1 to 0.27) |
| **United States of America** | 629218.1 (601298.1-646647.8) | 203.64 (195.36-208.90) |  | 1192434.2 (1124834.4-1238029.1) | 210.01 (199.54-217.40) |  | 0.08 (-0.15 to 0.31) |
| **United States Virgin Islands** | 89.5 (74.1-107.1) | 100.26 (83.36-119.00) |  | 192.2 (142.3-254.4) | 117.70 (87.55-154.83) |  | 0.46 (-0.16 to 1.07) |
| **Uruguay** | 9357.1 (8749.3-9962.9) | 242.49 (227.13-258.47) |  | 15524.6 (14148.9-16876.4) | 297.06 (272.60-321.45) |  | 0.7 (0.62 to 0.78) |
| **Uzbekistan** | 4901.3 (3956.0-6162.7) | 40.27 (32.18-51.65) |  | 18767.0 (14683.3-22943.3) | 64.29 (50.59-78.71) |  | 1.56 (1.21 to 1.91) |
| **Vanuatu** | 32.8 (25.0-43.0) | 48.55 (37.46-62.47) |  | 131.6 (102.9-164.9) | 69.14 (55.34-85.00) |  | 1.12 (0.92 to 1.31) |
| **Venezuela (Bolivarian Republic of)** | 3746.3 (3574.9-3915.8) | 35.69 (33.93-37.41) |  | 35409.7 (25215.4-46696.1) | 115.41 (82.32-151.79) |  | 3.72 (2.97 to 4.47) |
| **Viet Nam** | 14525.3 (11367.1-18795.6) | 35.21 (27.87-45.46) |  | 55890.4 (40539.1-69738.8) | 53.39 (38.86-66.41) |  | 1.37 (1.25 to 1.48) |
| **Yemen** | 1747.0 (1006.1-2734.9) | 32.94 (18.98-50.04) |  | 7031.4 (4491.8-9839.1) | 46.06 (29.57-64.18) |  | 1.11 (0.92 to 1.3) |
| **Zambia** | 2196.2 (1806.2-2667.9) | 70.60 (58.54-85.56) |  | 6392.5 (4417.1-8679.3) | 81.69 (57.80-110.21) |  | 0.46 (0.29 to 0.63) |
| **Zimbabwe** | 4906.7 (3922.3-5938.3) | 116.65 (93.17-141.28) |  | 14647.0 (10837.9-19047.4) | 190.96 (144.23-243.08) |  | 1.69 (1.33 to 2.04) |

*Abbreviations: ASR, age-standardized rate; DALYs, disability-adjusted life-years; AAPC, average annual percentage change; UI, uncertainty interval; CI, confidence interval.*

# Table S9. Case numbers and ASRs of incidence and prevalence of pancreatic cancer from 1990 to 2021 for both sexes globally.

| **Year** | **Incidence** | | | |  | **Prevalence** | | | |
| --- | --- | --- | --- | --- | --- | --- | --- | --- | --- |
|  | **Female** | | **Male** | |  | **Female** | | **Male** | |
|  | **Number**  **(95% UI)** | **ASR**  **(95% UI)** | **Number**  **(95% UI)** | **ASR**  **(95% UI)** |  | **Number**  **(95% UI)** | **ASR**  **(95% UI)** | **Number**  **(95% UI)** | **ASR**  **(95% UI)** |
| **1990** | 97509.7 (90497.6-103233.0) | 4.71 (4.34-4.99) | 110395.6 (104541.9-116510.2) | 6.30 (5.95-6.62) |  | 78554.0 (73543.4-83240.0) | 3.73 (3.47-3.95) | 94254.8 (89253.3-99391.3) | 5.09 (4.83-5.34) |
| **1991** | 100023.8 (93335.9-106364.0) | 4.72 (4.39-5.02) | 113351.2 (107673.9-119222.3) | 6.31 (6.00-6.62) |  | 80673.0 (75797.7-85608.1) | 3.74 (3.50-3.97) | 96851.7 (92061.2-101858.5) | 5.11 (4.86-5.36) |
| **1992** | 102799.2 (95278.3-108604.8) | 4.74 (4.38-5.01) | 116615.0 (110940.6-122485.9) | 6.34 (6.03-6.64) |  | 82973.8 (77376.3-87519.8) | 3.76 (3.50-3.97) | 99709.8 (94989.6-104704.7) | 5.14 (4.90-5.38) |
| **1993** | 106259.6 (98832.2-111992.4) | 4.79 (4.44-5.05) | 120083.8 (114378.5-125300.5) | 6.37 (6.07-6.64) |  | 85837.7 (80308.0-90377.5) | 3.81 (3.55-4.01) | 102673.5 (97991.1-106981.7) | 5.17 (4.92-5.38) |
| **1994** | 109167.4 (101532.7-114259.7) | 4.82 (4.46-5.05) | 123150.0 (118142.8-127704.7) | 6.38 (6.10-6.61) |  | 88232.3 (82758.3-92192.4) | 3.84 (3.58-4.01) | 105299.0 (101176.3-109103.3) | 5.18 (4.97-5.36) |
| **1995** | 111225.4 (103637.0-116730.9) | 4.80 (4.45-5.04) | 126036.8 (121032.7-130984.1) | 6.38 (6.13-6.63) |  | 89924.1 (84146.4-94255.9) | 3.83 (3.57-4.01) | 107811.8 (103585.6-111933.0) | 5.19 (4.99-5.38) |
| **1996** | 113669.7 (105554.5-119114.8) | 4.80 (4.44-5.04) | 128485.4 (123652.8-133461.6) | 6.37 (6.10-6.62) |  | 91992.5 (86000.7-96306.7) | 3.83 (3.57-4.02) | 109877.5 (105877.0-113816.6) | 5.18 (4.99-5.38) |
| **1997** | 116228.3 (108056.2-121733.0) | 4.80 (4.44-5.04) | 131122.1 (126173.3-136000.2) | 6.35 (6.10-6.61) |  | 94212.2 (88318.0-98701.4) | 3.84 (3.58-4.03) | 112231.7 (108319.9-116128.3) | 5.18 (4.99-5.37) |
| **1998** | 118913.2 (110355.1-124518.2) | 4.80 (4.43-5.03) | 134556.6 (129503.0-139298.2) | 6.37 (6.09-6.61) |  | 96858.4 (90573.7-101238.3) | 3.86 (3.59-4.04) | 115362.7 (111204.3-119179.9) | 5.21 (5.01-5.39) |
| **1999** | 122030.4 (114060.5-127647.4) | 4.81 (4.47-5.04) | 138189.5 (132490.4-143111.3) | 6.39 (6.09-6.62) |  | 100286.0 (94226.2-104644.0) | 3.91 (3.66-4.08) | 119137.2 (114513.5-123151.5) | 5.26 (5.04-5.44) |
| **2000** | 125653.7 (116429.4-132111.6) | 4.84 (4.46-5.09) | 142594.6 (137415.0-147572.8) | 6.43 (6.17-6.67) |  | 104555.2 (97840.1-109839.8) | 3.98 (3.71-4.19) | 123892.2 (119581.0-128081.8) | 5.35 (5.15-5.53) |
| **2001** | 128568.6 (119007.4-134460.6) | 4.83 (4.45-5.06) | 146390.6 (140747.3-151642.6) | 6.45 (6.16-6.68) |  | 108425.9 (101158.1-113412.6) | 4.03 (3.75-4.22) | 128222.8 (123587.9-132185.6) | 5.41 (5.20-5.59) |
| **2002** | 131548.4 (121419.8-138126.9) | 4.82 (4.43-5.06) | 150636.9 (144977.5-155457.7) | 6.47 (6.21-6.68) |  | 112028.5 (104287.2-117289.0) | 4.07 (3.77-4.26) | 132722.5 (128252.2-136617.7) | 5.46 (5.25-5.63) |
| **2003** | 135198.3 (123895.7-141985.9) | 4.83 (4.41-5.08) | 155694.1 (149752.0-160645.9) | 6.52 (6.26-6.72) |  | 115771.2 (106977.6-121495.4) | 4.10 (3.78-4.31) | 137562.0 (132829.5-141884.4) | 5.53 (5.33-5.70) |
| **2004** | 139413.7 (128551.4-146172.5) | 4.86 (4.46-5.10) | 160957.7 (154899.9-165979.2) | 6.57 (6.28-6.78) |  | 119713.2 (110708.1-125325.6) | 4.14 (3.81-4.34) | 142394.9 (137049.6-146548.9) | 5.58 (5.35-5.75) |
| **2005** | 144416.1 (133141.4-151434.1) | 4.90 (4.50-5.14) | 166909.3 (160026.8-172135.6) | 6.63 (6.34-6.85) |  | 124223.7 (114891.0-130295.7) | 4.19 (3.86-4.40) | 147738.3 (142012.6-152111.6) | 5.65 (5.40-5.81) |
| **2006** | 148501.7 (136517.0-155854.0) | 4.90 (4.50-5.16) | 171542.2 (164947.9-176961.8) | 6.64 (6.36-6.86) |  | 127975.7 (118448.2-133868.3) | 4.20 (3.88-4.40) | 152104.1 (146862.0-156713.6) | 5.67 (5.45-5.84) |
| **2007** | 153583.5 (140695.2-161799.9) | 4.93 (4.51-5.20) | 177566.1 (170977.3-184453.1) | 6.69 (6.38-6.94) |  | 132591.8 (122157.5-138932.4) | 4.23 (3.89-4.44) | 157502.9 (152024.8-163583.1) | 5.72 (5.49-5.94) |
| **2008** | 159108.1 (145080.2-167317.9) | 4.97 (4.52-5.23) | 184459.8 (177580.3-191242.8) | 6.75 (6.46-7.00) |  | 137439.2 (126202.4-144103.8) | 4.27 (3.91-4.48) | 163535.4 (157551.7-169065.0) | 5.78 (5.55-5.98) |
| **2009** | 164137.5 (148866.2-172161.9) | 4.98 (4.51-5.23) | 190714.3 (183121.1-197727.2) | 6.79 (6.48-7.04) |  | 142022.9 (129863.4-148773.2) | 4.29 (3.92-4.50) | 169035.9 (162845.7-175064.7) | 5.81 (5.57-6.02) |
| **2010** | 169586.5 (154495.9-178917.9) | 5.00 (4.55-5.28) | 198062.8 (189548.7-205642.2) | 6.85 (6.52-7.11) |  | 146790.0 (134275.0-154609.5) | 4.31 (3.94-4.55) | 175340.9 (168459.7-181832.9) | 5.87 (5.62-6.08) |
| **2011** | 174912.4 (158127.7-184921.9) | 5.01 (4.52-5.30) | 204510.4 (195838.4-213762.9) | 6.88 (6.55-7.18) |  | 151224.8 (137993.1-159066.9) | 4.32 (3.94-4.55) | 180617.8 (173130.3-188571.4) | 5.88 (5.64-6.13) |
| **2012** | 179843.2 (162103.2-189961.6) | 5.00 (4.51-5.29) | 211534.6 (200814.9-221200.0) | 6.91 (6.53-7.23) |  | 155323.1 (141684.2-163737.9) | 4.31 (3.93-4.55) | 186541.7 (177738.9-194991.3) | 5.91 (5.60-6.18) |
| **2013** | 184422.8 (165561.1-195714.4) | 4.98 (4.47-5.29) | 217734.6 (206533.8-227770.9) | 6.91 (6.54-7.23) |  | 159280.4 (144641.1-168596.0) | 4.30 (3.90-4.55) | 191884.9 (182730.6-200492.8) | 5.91 (5.62-6.18) |
| **2014** | 190192.4 (170339.2-200910.0) | 4.99 (4.47-5.27) | 223639.4 (211341.4-234528.3) | 6.90 (6.51-7.24) |  | 164153.4 (148451.3-173063.9) | 4.31 (3.89-4.54) | 196953.4 (186715.7-206133.6) | 5.91 (5.58-6.18) |
| **2015** | 196774.2 (176819.1-208476.7) | 5.02 (4.51-5.32) | 230812.7 (218984.6-243030.1) | 6.93 (6.54-7.30) |  | 169950.0 (153140.5-179432.0) | 4.34 (3.91-4.58) | 203223.0 (193330.6-213459.0) | 5.94 (5.63-6.23) |
| **2016** | 204732.2 (182194.5-217819.8) | 5.07 (4.51-5.39) | 239332.9 (223786.3-253101.2) | 6.98 (6.53-7.38) |  | 176853.4 (158641.1-187469.5) | 4.38 (3.94-4.65) | 210526.6 (198004.9-222184.4) | 5.99 (5.62-6.32) |
| **2017** | 211254.0 (188709.8-227741.5) | 5.08 (4.54-5.47) | 246977.6 (231267.8-263596.4) | 7.00 (6.54-7.46) |  | 182319.5 (163555.9-195589.4) | 4.39 (3.94-4.71) | 217206.5 (203912.8-231444.0) | 6.01 (5.64-6.39) |
| **2018** | 218776.3 (193682.9-233709.9) | 5.11 (4.52-5.45) | 255395.9 (238217.1-273298.5) | 7.03 (6.54-7.52) |  | 188609.2 (167229.9-200699.6) | 4.42 (3.92-4.70) | 224366.5 (209644.2-239406.3) | 6.03 (5.64-6.43) |
| **2019** | 226296.9 (198579.8-243419.6) | 5.13 (4.50-5.51) | 263565.1 (243615.5-284416.1) | 7.04 (6.50-7.57) |  | 194609.6 (172407.3-208843.2) | 4.43 (3.93-4.75) | 230912.3 (214133.5-249424.7) | 6.04 (5.60-6.50) |
| **2020** | 228998.2 (201700.6-248237.9) | 5.05 (4.45-5.47) | 267976.1 (246470.3-288245.8) | 6.97 (6.41-7.49) |  | 195960.2 (173441.3-212532.7) | 4.34 (3.85-4.71) | 233786.9 (215394.9-251520.1) | 5.96 (5.49-6.40) |
| **2021** | 234915.6 (205148.7-255434.6) | 5.05 (4.41-5.49) | 273617.1 (250808.5-299347.6) | 6.96 (6.37-7.59) |  | 200700.4 (176302.0-216717.9) | 4.34 (3.82-4.68) | 238300.2 (219016.1-260332.5) | 5.95 (5.47-6.49) |

*Abbreviations: ASR, age-standardized rate; UI, uncertainty interval.*

# Table S10. Case numbers and ASRs of mortality and DALYs of pancreatic cancer from 1990 to 2021 for both sexes globally.

| **Year** | **Mortality** | | | |  | **DALYs** | | | |
| --- | --- | --- | --- | --- | --- | --- | --- | --- | --- |
|  | **Female** | | **Male** | |  | **Female** | | **Male** | |
|  | **Number**  **(95% UI)** | **ASR**  **(95% UI)** | **Number**  **(95% UI)** | **ASR**  **(95% UI)** |  | **Number**  **(95% UI)** | **ASR**  **(95% UI)** | **Number**  **(95% UI)** | **ASR**  **(95% UI)** |
| **1990** | 100589.7 (93631.4-106565.1) | 4.90 (4.52-5.19) | 111023.0 (105146.9-117266.5) | 6.48 (6.13-6.82) |  | 2254144.6 (2113304.6-2406618.2) | 105.71 (98.83-112.86) | 2956334.2 (2782367.4-3138634.9) | 154.20 (145.54-163.26) |
| **1991** | 103137.2 (96223.5-109753.1) | 4.90 (4.56-5.22) | 113937.8 (108152.9-119940.2) | 6.49 (6.17-6.82) |  | 2305724.3 (2180870.7-2452144.9) | 105.80 (99.87-112.48) | 3029610.3 (2863012.6-3209535.1) | 154.37 (146.35-162.99) |
| **1992** | 105948.5 (98106.2-111852.0) | 4.92 (4.54-5.20) | 117123.2 (111300.0-123162.5) | 6.51 (6.19-6.83) |  | 2361051.8 (2224099.4-2496108.4) | 106.05 (99.72-112.14) | 3109907.7 (2954841.3-3280855.6) | 154.81 (147.23-162.85) |
| **1993** | 109401.4 (101704.5-115284.9) | 4.97 (4.60-5.24) | 120488.4 (114717.2-125856.9) | 6.53 (6.22-6.82) |  | 2434792.5 (2296852.8-2569742.1) | 107.09 (100.85-113.05) | 3196473.7 (3042758.6-3347133.0) | 155.52 (148.12-162.71) |
| **1994** | 112218.4 (104413.4-117488.7) | 4.99 (4.61-5.23) | 123391.4 (118270.8-128055.8) | 6.53 (6.24-6.77) |  | 2491531.7 (2357346.3-2609233.0) | 107.31 (101.33-112.33) | 3268286.0 (3127309.9-3401453.3) | 155.50 (148.94-161.56) |
| **1995** | 114105.6 (106195.8-119821.9) | 4.96 (4.59-5.21) | 126071.0 (120950.7-131114.8) | 6.52 (6.25-6.77) |  | 2524936.0 (2381289.9-2650962.1) | 106.55 (100.28-111.80) | 3333635.0 (3192858.6-3479034.7) | 155.26 (148.69-161.62) |
| **1996** | 116341.6 (108090.5-122033.4) | 4.95 (4.57-5.20) | 128343.2 (123443.0-133446.3) | 6.49 (6.21-6.75) |  | 2565262.3 (2427941.4-2680460.6) | 105.98 (100.13-110.80) | 3381429.9 (3247004.9-3513485.5) | 154.19 (148.25-160.26) |
| **1997** | 118803.4 (110409.8-124485.5) | 4.94 (4.56-5.19) | 130845.5 (125949.8-135946.0) | 6.47 (6.20-6.74) |  | 2611066.0 (2481330.9-2730271.0) | 105.55 (100.07-110.45) | 3436107.0 (3308898.5-3567652.9) | 153.28 (147.48-159.21) |
| **1998** | 121401.1 (112544.7-127177.1) | 4.93 (4.54-5.17) | 134172.1 (129039.3-139083.0) | 6.48 (6.20-6.72) |  | 2663041.0 (2513054.3-2785441.2) | 105.29 (99.14-110.08) | 3507695.3 (3368564.7-3635572.1) | 153.11 (147.01-158.82) |
| **1999** | 124487.2 (116200.5-130272.1) | 4.93 (4.58-5.17) | 137678.7 (132096.2-142723.2) | 6.49 (6.19-6.73) |  | 2724719.7 (2585232.2-2841096.2) | 105.32 (99.80-109.88) | 3589757.3 (3446342.9-3723378.3) | 153.13 (146.98-158.61) |
| **2000** | 128188.8 (118838.3-134843.9) | 4.96 (4.56-5.22) | 142104.3 (136835.1-147268.5) | 6.53 (6.26-6.78) |  | 2796684.0 (2653463.5-2925056.5) | 105.69 (100.16-110.59) | 3692650.5 (3557965.5-3822532.7) | 154.01 (148.17-159.40) |
| **2001** | 131114.7 (121386.1-137238.4) | 4.94 (4.55-5.18) | 145885.0 (140126.5-151326.5) | 6.55 (6.25-6.79) |  | 2852522.7 (2701428.7-2972766.7) | 105.30 (99.50-109.87) | 3775596.1 (3635412.1-3913629.9) | 153.88 (147.94-159.63) |
| **2002** | 134044.0 (123648.3-140750.8) | 4.93 (4.52-5.18) | 150026.4 (144433.1-154953.6) | 6.56 (6.29-6.78) |  | 2910174.5 (2744651.7-3045946.3) | 104.87 (98.69-109.80) | 3874104.1 (3731437.1-4004884.5) | 154.05 (148.13-159.16) |
| **2003** | 137589.6 (125806.5-144630.5) | 4.93 (4.49-5.19) | 154931.5 (148751.2-159930.0) | 6.60 (6.33-6.81) |  | 2979184.4 (2801991.5-3126426.8) | 104.83 (98.39-109.93) | 3988099.4 (3843802.0-4129620.8) | 154.79 (148.93-160.12) |
| **2004** | 141630.2 (130763.6-148572.5) | 4.95 (4.55-5.20) | 159924.0 (153884.1-165058.4) | 6.64 (6.34-6.85) |  | 3055477.3 (2871511.0-3192280.3) | 104.92 (98.48-109.67) | 4103141.9 (3950001.7-4238355.1) | 155.38 (149.55-160.42) |
| **2005** | 146309.1 (134850.5-153516.3) | 4.98 (4.57-5.23) | 165450.2 (158564.2-170733.3) | 6.69 (6.38-6.90) |  | 3152014.3 (2987637.1-3289503.8) | 105.60 (99.89-110.20) | 4234208.3 (4079548.6-4370657.2) | 156.43 (150.36-161.48) |
| **2006** | 150032.8 (137765.3-157605.2) | 4.97 (4.55-5.23) | 169607.0 (163081.2-175194.3) | 6.67 (6.37-6.90) |  | 3220641.5 (3031494.7-3355850.9) | 105.15 (98.79-109.62) | 4326341.5 (4190508.5-4474696.2) | 155.76 (150.59-161.08) |
| **2007** | 154862.5 (141808.3-163155.3) | 4.98 (4.55-5.26) | 175285.5 (168979.7-182082.0) | 6.70 (6.41-6.97) |  | 3311208.1 (3114293.0-3459990.9) | 105.26 (98.83-110.06) | 4448804.6 (4301903.4-4618172.9) | 156.04 (150.38-161.91) |
| **2008** | 160239.4 (145969.4-168570.3) | 5.01 (4.55-5.28) | 181938.1 (175106.5-188644.9) | 6.76 (6.48-7.02) |  | 3411321.2 (3200957.8-3552337.8) | 105.59 (98.99-110.03) | 4597930.8 (4432693.6-4771351.0) | 157.06 (151.16-163.06) |
| **2009** | 165075.6 (150006.8-173092.2) | 5.02 (4.55-5.27) | 187987.3 (180141.3-195065.6) | 6.79 (6.48-7.04) |  | 3505299.9 (3274367.7-3641929.3) | 105.59 (98.50-109.73) | 4729778.9 (4558106.3-4900469.0) | 157.31 (151.35-163.02) |
| **2010** | 170398.7 (155084.3-179735.7) | 5.03 (4.57-5.31) | 195150.4 (186977.0-202753.0) | 6.85 (6.51-7.11) |  | 3605892.7 (3345987.9-3764213.6) | 105.73 (98.02-110.42) | 4889133.9 (4708005.9-5082318.7) | 158.40 (152.19-164.51) |
| **2011** | 175625.8 (158968.4-185537.8) | 5.04 (4.55-5.33) | 201482.5 (192811.5-210606.9) | 6.87 (6.55-7.18) |  | 3699569.3 (3462097.7-3870215.1) | 105.52 (98.71-110.39) | 5023864.4 (4798742.6-5276543.0) | 158.49 (151.67-166.11) |
| **2012** | 180473.4 (162998.4-190748.6) | 5.03 (4.53-5.31) | 208331.9 (197725.0-218098.2) | 6.90 (6.52-7.22) |  | 3788240.2 (3513728.0-3965696.2) | 105.07 (97.43-110.01) | 5175130.5 (4937805.8-5434733.9) | 158.85 (151.44-166.66) |
| **2013** | 184934.2 (166306.3-196252.7) | 5.00 (4.49-5.31) | 214360.7 (203465.2-224200.5) | 6.89 (6.52-7.20) |  | 3870721.6 (3598958.2-4063799.3) | 104.41 (97.05-109.61) | 5299867.8 (5044747.6-5561356.9) | 158.34 (150.97-165.85) |
| **2014** | 190627.9 (170994.0-201303.0) | 5.01 (4.49-5.29) | 220174.1 (207962.3-231023.1) | 6.88 (6.48-7.21) |  | 3977277.0 (3686119.0-4169033.3) | 104.31 (96.64-109.34) | 5420768.9 (5139478.8-5712005.4) | 157.65 (149.16-165.88) |
| **2015** | 197191.2 (177082.1-208917.2) | 5.03 (4.52-5.33) | 227287.2 (215510.2-239561.2) | 6.91 (6.53-7.29) |  | 4103962.9 (3803157.5-4312279.9) | 104.70 (97.03-110.03) | 5570218.6 (5277976.5-5884689.6) | 157.79 (149.66-166.64) |
| **2016** | 205131.9 (182838.8-218177.8) | 5.08 (4.53-5.40) | 235797.4 (220781.5-249374.7) | 6.96 (6.50-7.37) |  | 4258570.3 (3915733.7-4490970.5) | 105.64 (97.15-111.40) | 5745985.5 (5408990.9-6101679.4) | 158.51 (149.34-168.00) |
| **2017** | 211740.7 (189483.4-228327.2) | 5.09 (4.55-5.48) | 243343.5 (227722.7-259765.6) | 6.97 (6.52-7.44) |  | 4386309.6 (4062621.2-4696379.6) | 105.79 (98.03-113.26) | 5908564.1 (5520403.0-6337362.0) | 158.64 (148.53-169.93) |
| **2018** | 219270.5 (194394.5-234326.2) | 5.11 (4.54-5.46) | 251695.0 (235238.8-269457.6) | 7.00 (6.53-7.49) |  | 4532572.9 (4137693.4-4801792.2) | 106.31 (97.14-112.60) | 6087111.8 (5649294.0-6550676.5) | 159.11 (147.98-171.10) |
| **2019** | 226911.4 (199954.1-244203.5) | 5.13 (4.53-5.52) | 259957.8 (240083.1-280345.0) | 7.01 (6.47-7.55) |  | 4675081.4 (4256553.9-5007608.7) | 106.62 (97.10-114.18) | 6261225.4 (5816150.9-6819162.0) | 159.33 (147.99-173.18) |
| **2020** | 229915.0 (203050.4-249567.6) | 5.06 (4.47-5.49) | 264592.8 (243477.8-284622.2) | 6.95 (6.41-7.47) |  | 4730999.2 (4264395.4-5114256.3) | 105.11 (94.79-113.59) | 6347215.7 (5840107.0-6893641.9) | 157.64 (145.14-170.81) |
| **2021** | 235714.7 (206198.6-256636.8) | 5.06 (4.43-5.51) | 270037.4 (247469.9-295172.9) | 6.93 (6.35-7.55) |  | 4854242.7 (4346324.1-5249520.9) | 105.24 (94.33-113.82) | 6462720.6 (5913394.6-7103691.3) | 157.12 (143.87-172.29) |

*Abbreviations: ASR, age-standardized rate; DALYs, disability-adjusted life-years; UI, uncertainty interval*

# Table S11. Age distribution of pancreatic cancer case numbers and rates per 100,000 population of incidence by sex in 2021.

| **Age groups** | **Male** | |  | | **Female** | | |
| --- | --- | --- | --- | --- | --- | --- | --- |
|  | **Number**  **(95% UI)** | **Rate/10,0000 population**  **(95% UI)** |  | **Number**  **(95% UI)** | | **Rate/10,0000 population**  **(95% UI)** |  |
| **<5 years** | 0 | 0 |  | 0 | | 0 |  |
| **5-9 years** | 0 | 0 |  | 0 | | 0 |  |
| **10-14 years** | 0 | 0 |  | 0 | | 0 |  |
| **15-19 years** | 68.1 (59.5-79.3) | 0.02 (0.02-0.02) |  | 69.5 (61.6-80.7) | | 0.02 (0.02-0.03) |  |
| **20-24 years** | 156.9 (140.3-175.8) | 0.05 (0.05-0.06) |  | 132.3 (115.8-154.1) | | 0.05 (0.04-0.05) |  |
| **25-29 years** | 401.4 (360.2-445.2) | 0.14 (0.12-0.15) |  | 282.6 (251.6-314.0) | | 0.10 (0.09-0.11) |  |
| **30-34 years** | 1433.3 (1275.0-1606.3) | 0.47 (0.42-0.53) |  | 656.4 (585.4-733.4) | | 0.22 (0.20-0.25) |  |
| **35-39 years** | 3145.9 (2791.3-3523.7) | 1.11 (0.99-1.24) |  | 1380.4 (1259.8-1502.7) | | 0.50 (0.45-0.54) |  |
| **40-44 years** | 5710.4 (5097.1-6393.4) | 2.26 (2.02-2.54) |  | 2661.4 (2441.0-2890.7) | | 1.07 (0.98-1.17) |  |
| **45-49 years** | 10254.7 (9119.6-11618.6) | 4.31 (3.83-4.88) |  | 5177.3 (4741.2-5626.9) | | 2.20 (2.01-2.39) |  |
| **50-54 years** | 18529.6 (16481.9-20885.1) | 8.35 (7.42-9.41) |  | 10089.9 (9160.9-11156.1) | | 4.53 (4.11-5.00) |  |
| **55-59 years** | 27816.4 (25094.4-30974.1) | 14.28 (12.89-15.91) |  | 16713.0 (15384.0-18206.6) | | 8.31 (7.65-9.06) |  |
| **60-64 years** | 35042.0 (31988.4-38355.3) | 22.53 (20.57-24.66) |  | 24106.6 (22229.2-25951.1) | | 14.65 (13.51-15.77) |  |
| **65-69 years** | 42896.4 (38974.3-47136.7) | 32.54 (29.56-35.75) |  | 32481.6 (29396.1-35193.8) | | 22.56 (20.41-24.44) |  |
| **70-74 years** | 44343.2 (40918.5-48326.9) | 46.00 (42.45-50.14) |  | 37392.6 (33442.1-40332.9) | | 34.16 (30.56-36.85) |  |
| **75-79 years** | 33797.0 (30829.0-36700.0) | 56.53 (51.56-61.38) |  | 33048.1 (28405.5-35829.8) | | 45.84 (39.40-49.70) |  |
| **80-84 years** | 26397.3 (23436.9-28414.3) | 72.02 (63.94-77.52) |  | 31097.0 (25232.7-34405.5) | | 61.06 (49.54-67.55) |  |
| **85-89 years** | 16574.6 (14297.0-17956.1) | 96.07 (82.87-104.08) |  | 23232.5 (17348.8-26459.2) | | 81.61 (60.94-92.94) |  |
| **90-94 years** | 5885.1 (4942.3-6440.9) | 100.97 (84.80-110.51) |  | 12648.0 (9205.4-14589.3) | | 104.87 (76.32-120.96) |  |
| **95+ years** | 1164.7 (896.8-1305.3) | 77.03 (59.31-86.32) |  | 3746.5 (2574.2-4429.1) | | 95.13 (65.36-112.46) |  |

*Abbreviations:UI, uncertainty interval.*

# Table S12. Age distribution of pancreatic cancer case numbers and rates per 100,000 population of prevalence by sex in 2021.

| **Age groups** | **Male** | |  | | **Female** | |
| --- | --- | --- | --- | --- | --- | --- |
|  | **Number**  **(95% UI)** | **Rate/10,0000 population**  **(95% UI)** |  | **Number**  **(95% UI)** | | **Rate/10,0000 population**  **(95% UI)** |
| **<5 years** | 0 | 0 |  | 0 | | 0 |
| **5-9 years** | 0 | 0 |  | 0 | | 0 |
| **10-14 years** | 0 | 0 |  | 0 | | 0 |
| **15-19 years** | 102.5 (90.1-118.6) | 0.03 (0.03-0.04) |  | 111.1 (99.3-127.9) | | 0.04 (0.03-0.04) |
| **20-24 years** | 236.3 (212.7-263.7) | 0.08 (0.07-0.09) |  | 214.7 (189.8-246.9) | | 0.07 (0.06-0.08) |
| **25-29 years** | 910.0 (819.5-1007.6) | 0.31 (0.28-0.34) |  | 714.8 (639.6-790.4) | | 0.25 (0.22-0.27) |
| **30-34 years** | 3466.2 (3092.1-3873.8) | 1.13 (1.01-1.27) |  | 1764.7 (1585.1-1954.2) | | 0.59 (0.53-0.65) |
| **35-39 years** | 5360.3 (4809.3-5964.9) | 1.89 (1.70-2.11) |  | 2636.9 (2423.9-2850.4) | | 0.95 (0.87-1.03) |
| **40-44 years** | 6499.9 (5864.9-7200.0) | 2.58 (2.33-2.86) |  | 3517.3 (3258.3-3774.9) | | 1.42 (1.31-1.52) |
| **45-49 years** | 10942.9 (9771.3-12328.6) | 4.60 (4.11-5.18) |  | 5776.6 (5334.7-6231.1) | | 2.45 (2.26-2.64) |
| **50-54 years** | 19565.2 (17506.8-21933.5) | 8.81 (7.89-9.88) |  | 11128.2 (10154.1-12221.9) | | 4.99 (4.55-5.48) |
| **55-59 years** | 28654.6 (26053.0-31701.9) | 14.72 (13.38-16.28) |  | 17697.1 (16325.4-19190.9) | | 8.80 (8.12-9.55) |
| **60-64 years** | 32330.2 (29624.2-35311.1) | 20.79 (19.05-22.70) |  | 22741.6 (21069.6-24395.0) | | 13.82 (12.81-14.83) |
| **65-69 years** | 35987.9 (32843.1-39539.4) | 27.30 (24.91-29.99) |  | 27921.8 (25438.1-30211.9) | | 19.39 (17.66-20.98) |
| **70-74 years** | 34012.8 (31431.8-36945.6) | 35.29 (32.61-38.33) |  | 29936.0 (26759.3-32330.9) | | 27.35 (24.45-29.54) |
| **75-79 years** | 23910.0 (21804.9-25846.4) | 39.99 (36.47-43.23) |  | 24456.0 (20884.6-26717.9) | | 33.92 (28.97-37.06) |
| **80-84 years** | 21065.7 (18375.7-23335.7) | 57.48 (50.14-63.67) |  | 25303.8 (20071.6-28724.4) | | 49.68 (39.41-56.40) |
| **85-89 years** | 10889.2 (9240.4-12102.7) | 63.12 (53.56-70.15) |  | 16071.6 (12052.4-19059.0) | | 56.45 (42.33-66.95) |
| **90-94 years** | 3782.8 (3177.1-4149.7) | 64.90 (54.51-71.20) |  | 8830.7 (6305.3-10450.9) | | 73.22 (52.28-86.65) |
| **95+ years** | 583.7 (449.4-654.1) | 38.61 (29.72-43.26) |  | 1877.5 (1289.6-2219.5) | | 47.67 (32.75-56.36) |

*Abbreviations: UI, uncertainty interval*

# Table S13. Age distribution of pancreatic cancer case numbers and rates per 100,000 population of mortality by sex in 2021.

| **Age groups** | **Male** | |  | **Female** | |
| --- | --- | --- | --- | --- | --- |
|  | **Number**  **(95% UI)** | **Rate/10,0000 population**  **(95% UI)** |  | **Number**  **(95% UI)** | **Rate/10,0000 population**  **(95% UI)** |
| **<5 years** | 0 | 0 |  | 0 | 0 |
| **5-9 years** | 0 | 0 |  | 0 | 0 |
| **10-14 years** | 0 | 0 |  | 0 | 0 |
| **15-19 years** | 63.2 (55.1-73.6) | 0.02 (0.02-0.02) |  | 64.3 (56.7-75.3) | 0.02 (0.02-0.02) |
| **20-24 years** | 150.9 (134.5-169.4) | 0.05 (0.04-0.06) |  | 126.2 (109.7-148.1) | 0.04 (0.04-0.05) |
| **25-29 years** | 351.3 (314.4-390.5) | 0.12 (0.11-0.13) |  | 244.1 (216.9-271.5) | 0.08 (0.07-0.09) |
| **30-34 years** | 1232.8 (1096.1-1383.5) | 0.40 (0.36-0.45) |  | 550.9 (489.5-615.7) | 0.18 (0.16-0.21) |
| **35-39 years** | 2554.6 (2260.1-2859.7) | 0.90 (0.80-1.01) |  | 1089.7 (991.4-1187.3) | 0.39 (0.36-0.43) |
| **40-44 years** | 4829.3 (4309.6-5393.2) | 1.92 (1.71-2.14) |  | 2178.2 (1993.9-2368.5) | 0.88 (0.80-0.95) |
| **45-49 years** | 9069.4 (8047.5-10286.5) | 3.81 (3.38-4.32) |  | 4490.7 (4095.0-4892.6) | 1.91 (1.74-2.08) |
| **50-54 years** | 16749.6 (14891.3-18886.8) | 7.55 (6.71-8.51) |  | 8997.4 (8159.3-9966.7) | 4.04 (3.66-4.47) |
| **55-59 years** | 26016.8 (23432.4-28919.2) | 13.36 (12.03-14.85) |  | 15459.2 (14210.9-16865.5) | 7.69 (7.07-8.39) |
| **60-64 years** | 33479.8 (30552.0-36628.6) | 21.53 (19.64-23.55) |  | 22857.1 (21040.0-24660.1) | 13.89 (12.79-14.99) |
| **65-69 years** | 42104.9 (38223.5-46279.6) | 31.94 (28.99-35.10) |  | 31747.0 (28814.6-34467.4) | 22.05 (20.01-23.93) |
| **70-74 years** | 44680.3 (41108.5-48726.0) | 46.35 (42.65-50.55) |  | 37609.0 (33681.8-40677.7) | 34.36 (30.77-37.17) |
| **75-79 years** | 35227.5 (32246.9-38287.8) | 58.92 (53.94-64.04) |  | 34483.3 (29981.0-37418.8) | 47.83 (41.58-51.90) |
| **80-84 years** | 27672.9 (24701.3-29791.6) | 75.50 (67.39-81.28) |  | 32631.7 (27038.2-35987.4) | 64.07 (53.09-70.66) |
| **85-89 years** | 17451.9 (15241.9-18900.4) | 101.15 (88.35-109.55) |  | 24135.0 (18409.0-27321.7) | 84.78 (64.66-95.97) |
| **90-94 years** | 6749.7 (5719.6-7335.7) | 115.81 (98.13-125.86) |  | 13831.5 (10149.9-15923.4) | 114.68 (84.16-132.03) |
| **95+ years** | 1652.6 (1266.6-1848.6) | 109.30 (83.77-122.26) |  | 5219.5 (3611.3-6150.2) | 132.53 (91.70-156.17) |

*Abbreviations: UI, uncertainty interval.*

# Table S14. Age distribution of pancreatic cancer case numbers and rates per 100,000 population of DALYs by sex in 2021.

| **Age groups** | **Male** | |  | **Female** | |
| --- | --- | --- | --- | --- | --- |
|  | **Number**  **(95% UI)** | **Rate/10,0000 population**  **(95% UI)** |  | **Number**  **(95% UI)** | **Rate/10,0000 population**  **(95% UI)** |
| **<5 years** | 0 | 0 |  | 0 | 0 |
| **5-9 years** | 0 | 0 |  | 0 | 0 |
| **10-14 years** | 0 | 0 |  | 0 | 0 |
| **15-19 years** | 4593.2 (4004.3-5348.8) | 1.43 (1.25-1.67) |  | 4684.3 (4125.5-5481.2) | 1.54 (1.36-1.81) |
| **20-24 years** | 10239.8 (9132.0-11488.8) | 3.37 (3.01-3.79) |  | 8569.9 (7445.0-10049.6) | 2.92 (2.53-3.42) |
| **25-29 years** | 22114.7 (19777.2-24584.9) | 7.44 (6.65-8.27) |  | 15374.3 (13661.0-17104.0) | 5.28 (4.69-5.88) |
| **30-34 years** | 71480.1 (63593.8-80188.7) | 23.39 (20.81-26.24) |  | 31953.1 (28433.4-35687.6) | 10.69 (9.51-11.94) |
| **35-39 years** | 135677.7 (119985.9-151915.1) | 47.93 (42.39-53.67) |  | 57864.8 (52662.6-63065.3) | 20.83 (18.96-22.70) |
| **40-44 years** | 232330.9 (207343.4-259523.9) | 92.14 (82.23-102.92) |  | 104791.6 (95884.4-113726.4) | 42.24 (38.65-45.84) |
| **45-49 years** | 391592.6 (347390.9-443986.3) | 164.63 (146.05-186.66) |  | 193907.0 (176728.2-211058.2) | 82.29 (75.00-89.57) |
| **50-54 years** | 643595.2 (571993.0-726064.1) | 289.93 (257.68-327.08) |  | 345608.6 (313226.9-381980.0) | 155.02 (140.50-171.34) |
| **55-59 years** | 877981.9 (790629.0-976011.3) | 450.88 (406.02-501.23) |  | 521523.5 (478971.6-569830.1) | 259.46 (238.29-283.49) |
| **60-64 years** | 973818.6 (888372.0-1065037.7) | 626.10 (571.16-684.75) |  | 664546.5 (612097.4-716758.9) | 403.95 (372.07-435.69) |
| **65-69 years** | 1032284.6 (937361.6-1135456.7) | 783.02 (711.02-861.28) |  | 777620.8 (704678.5-844261.6) | 539.98 (489.33-586.26) |
| **70-74 years** | 901047.8 (828033.1-982605.8) | 934.78 (859.03-1019.39) |  | 757587.0 (679093.5-817054.6) | 692.19 (620.47-746.52) |
| **75-79 years** | 568151.3 (518981.2-617669.0) | 950.30 (868.05-1033.12) |  | 554191.9 (482074.8-601836.4) | 768.67 (668.64-834.75) |
| **80-84 years** | 349748.9 (311810.8-376970.2) | 954.25 (850.74-1028.52) |  | 410841.0 (341666.7-452896.3) | 806.66 (670.84-889.23) |
| **85-89 years** | 175464.2 (153344.8-189906.1) | 1017.03 (888.82-1100.74) |  | 241698.3 (184983.7-273438.5) | 848.98 (649.77-960.47) |
| **90-94 years** | 59155.3 (50109.1-64293.4) | 1014.93 (859.72-1103.09) |  | 121156.1 (88639.2-139361.5) | 1004.54 (734.93-1155.48) |
| **95+ years** | 13443.9 (10307.9-15034.8) | 889.12 (681.72-994.34) |  | 42324.2 (29335.8-49889.8) | 1074.69 (744.89-1266.80) |

*Abbreviations: DALYs, disability-adjusted life years; UI, uncertainty interval.*

# Table S15. Case numbers and percentage contribution of risk factors to all-age DALYs of pancreatic cancer in 2021, for both sexes, globally and by regions.

| **Location** | **Smoking** | |  | **High BMI** | |  | **High fasting plasma glucose** | |
| --- | --- | --- | --- | --- | --- | --- | --- | --- |
|  | **Number**  **(95 % UI)** | **Percent**  **(95 % UI)** |  | **Number**  **(95 % UI)** | **Percent**  **(95 % UI)** |  | **Number**  **(95 % UI)** | **Percent**  **(95 % UI)** |
| **Global** | 1789502.8 (1567220.8-2042056.6) | 15.81% (14.23%-17.37%) |  | 223367.7 (-47781.5-626549.7) | 1.98% (-0.41%-5.58%) |  | 2751644.1 (315351.3-5201444.2) | 24.31% (2.81%-46.09%) |
| **Andean Latin America** | 5405.3 (4023.7-7082.6) | 7.24% (6.36%-8.24%) |  | 2178.1 (-169.6-5783.5) | 2.92% (-0.22%-7.64%) |  | 16534.6 (1785.7-34252.7) | 22.05% (2.36%-43.23%) |
| **Australasia** | 8877.0 (7537.4-10408.5) | 10.44% (9.04%-12.07%) |  | 3413.1 (-73.9-8009.9) | 4.03% (-0.09%-9.69%) |  | 20342.9 (2571.5-35621.1) | 23.93% (3.01%-42.59%) |
| **Caribbean** | 8629.1 (7272.4-10391.7) | 12.68% (11.29%-14.14%) |  | 1732.7 (-165.8-4685.1) | 2.55% (-0.23%-6.54%) |  | 18427.6 (2151.8-36337.3) | 27.04% (3.15%-51.38%) |
| **Central Asia** | 14922.0 (12859.7-17248.0) | 15.34% (14.09%-16.68%) |  | 2939.8 (-222.9-7593.3) | 3.01% (-0.22%-7.78%) |  | 20187.5 (2119.6-40591.3) | 20.71% (2.14%-42.14%) |
| **Central Europe** | 82013.2 (71556.7-92023.0) | 17.03% (15.48%-18.55%) |  | 17599.3 (-598.5-43000.7) | 3.66% (-0.12%-9.05%) |  | 136586.5 (16621.5-254704.0) | 28.35% (3.40%-52.22%) |
| **Central Latin America** | 25143.2 (21172.5-29255.2) | 8.67% (7.70%-9.72%) |  | 11548.1 (-260.4-28149.5) | 3.99% (-0.10%-9.56%) |  | 83642.1 (10371.1-159644.0) | 28.82% (3.29%-53.95%) |
| **Central Sub-Saharan Africa** | 2814.0 (1955.1-4005.1) | 7.84% (6.68%-9.03%) |  | 261.6 (-290.4-1152.4) | 0.73% (-0.73%-3.15%) |  | 7840.8 (931.0-15962.9) | 21.78% (2.48%-42.32%) |
| **East Asia** | 617563.9 (467039.3-787817.4) | 20.33% (17.74%-22.91%) |  | 7696.9 (-33871.8-78317.0) | 0.26% (-1.09%-2.79%) |  | 629247.4 (71584.3-1197418.9) | 20.72% (2.40%-39.88%) |
| **Eastern Europe** | 121954.4 (106677.5-137712.2) | 16.85% (15.25%-18.53%) |  | 28244.2 (-684.2-68757.8) | 3.91% (-0.10%-9.45%) |  | 133011.1 (14146.2-263603.4) | 18.37% (1.99%-37.23%) |
| **Eastern Sub-Saharan Africa** | 6757.6 (5119.8-9102.1) | 7.13% (6.20%-8.06%) |  | 179.5 (-945.1-1973.2) | 0.19% (-0.92%-2.15%) |  | 12720.7 (1243.9-27909.7) | 13.47% (1.28%-28.55%) |
| **High-income Asia Pacific** | 110310.9 (96130.0-125424.1) | 12.76% (11.24%-14.31%) |  | -7517.7 (-17946.1-6049.6) | -0.85% (-1.96%-0.80%) |  | 219416.5 (27003.8-397222.3) | 25.39% (3.18%-45.86%) |
| **High-income North America** | 196657.3 (169599.4-227339.2) | 15.02% (13.05%-17.17%) |  | 64349.1 (421.2-142173.1) | 4.93% (0.03%-11.12%) |  | 437630.5 (54347.1-788873.4) | 33.43% (4.21%-60.49%) |
| **North Africa and Middle East** | 80277.1 (67441.1-93215.4) | 15.83% (14.33%-17.34%) |  | 25982.1 (515.5-58488.2) | 5.14% (0.11%-11.52%) |  | 143350.7 (16496.5-271338.0) | 28.28% (3.33%-52.41%) |
| **Oceania** | 703.0 (538.0-935.1) | 14.81% (13.15%-16.46%) |  | 106.8 (-13.9-293.6) | 2.27% (-0.32%-6.32%) |  | 1350.3 (159.3-2671.9) | 28.49% (3.54%-52.66%) |
| **South Asia** | 64073.2 (53827.4-74888.2) | 11.42% (10.12%-12.70%) |  | -186.7 (-5210.2-8367.7) | -0.03% (-0.95%-1.42%) |  | 142674.0 (15821.9-271951.1) | 25.44% (2.93%-47.98%) |
| **Southeast Asia** | 96075.6 (80159.6-114510.5) | 16.08% (14.32%-17.91%) |  | -244.8 (-6506.2-10152.5) | -0.04% (-1.08%-1.68%) |  | 122391.6 (12430.6-254200.0) | 20.46% (2.08%-41.67%) |
| **Southern Latin America** | 26129.1 (22977.3-29697.8) | 14.81% (13.25%-16.57%) |  | 7225.5 (-159.4-17548.5) | 4.11% (-0.09%-9.93%) |  | 46081.5 (5387.0-87772.9) | 26.13% (3.03%-49.63%) |
| **Southern Sub-Saharan Africa** | 10989.6 (9322.8-12750.1) | 12.28% (11.06%-13.60%) |  | 3535.4 (3.2-8154.1) | 3.95% (0.00%-9.09%) |  | 19492.1 (1830.7-39985.0) | 21.76% (2.12%-44.30%) |
| **Tropical Latin America** | 48804.2 (42173.3-55816.0) | 12.78% (11.14%-14.55%) |  | 11589.1 (-908.1-29503.1) | 3.05% (-0.24%-7.90%) |  | 94294.1 (10886.9-177957.2) | 24.70% (2.81%-46.84%) |
| **Western Europe** | 256629.6 (226671.5-290103.6) | 14.77% (13.17%-16.41%) |  | 41535.2 (-6867.4-117850.2) | 2.40% (-0.40%-6.96%) |  | 427164.3 (44747.4-845571.0) | 24.57% (2.62%-48.49%) |
| **Western Sub-Saharan Africa** | 4773.4 (3811.2-5868.2) | 4.71% (4.15%-5.34%) |  | 1200.3 (-566.2-4198.2) | 1.18% (-0.57%-3.94%) |  | 19257.1 (1999.5-39693.8) | 18.95% (2.00%-38.01%) |

*Abbreviations: ASR, age-standardized rate; DALYs, disability-adjusted life-years; UI, uncertainty interval; BMI, body mass index.*

# Table S16. The slope index of inequality and concentration index of pancreatic cancer mortality and DALYs related to SDI.

| **Measures** | **Health inequality metrics** | **Year** | **Value** | **95% CI** |
| --- | --- | --- | --- | --- |
| **DALYs** | Slope index of inequality | 1990 | 193.9 | 162.0 to 225.9 |
|  |  | 2019 | 328.0 | 290.7 to 365.2 |
|  | Health concentration index | 1990 | 0.46 | 0.42 to 0.51 |
|  |  | 2019 | 0.47 | 0.43 to 0.51 |
| **mortality** | Slope index of inequality | 1990 | 8.4 | 6.9 to 9.8 |
|  |  | 2019 | 15.8 | 13.9 to 17.6 |
|  | Health concentration index | 1990 | 0.51 | 0.46 to 0.56 |
|  |  | 2019 | 0.51 | 0.47 to 0.56 |

*Abbreviations: SDI, sociodemographic index; CI, confidence interval; DALYs, disability-adjusted life years*

**
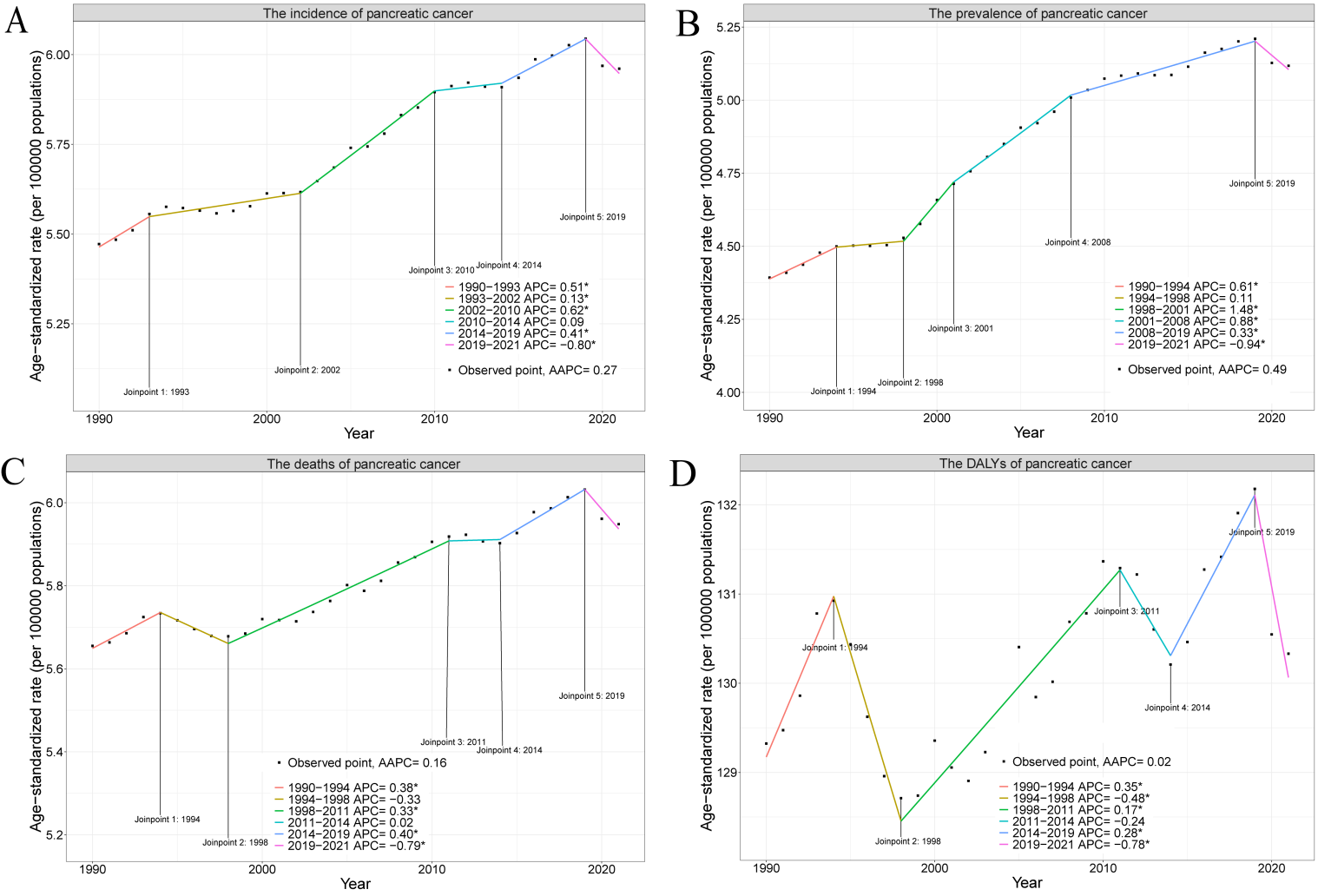
**

# **Figure S1.** The APC and AAPC of ASR for incidence (A), prevalence (B), mortality (C) and DALYs (D) pancreatic cancer at the global level based on the joinpoint analysis.

*Abbreviations: APC, Annual percentage change; AAPC, average annual percentage change; DALYs, disability-adjusted life years.*


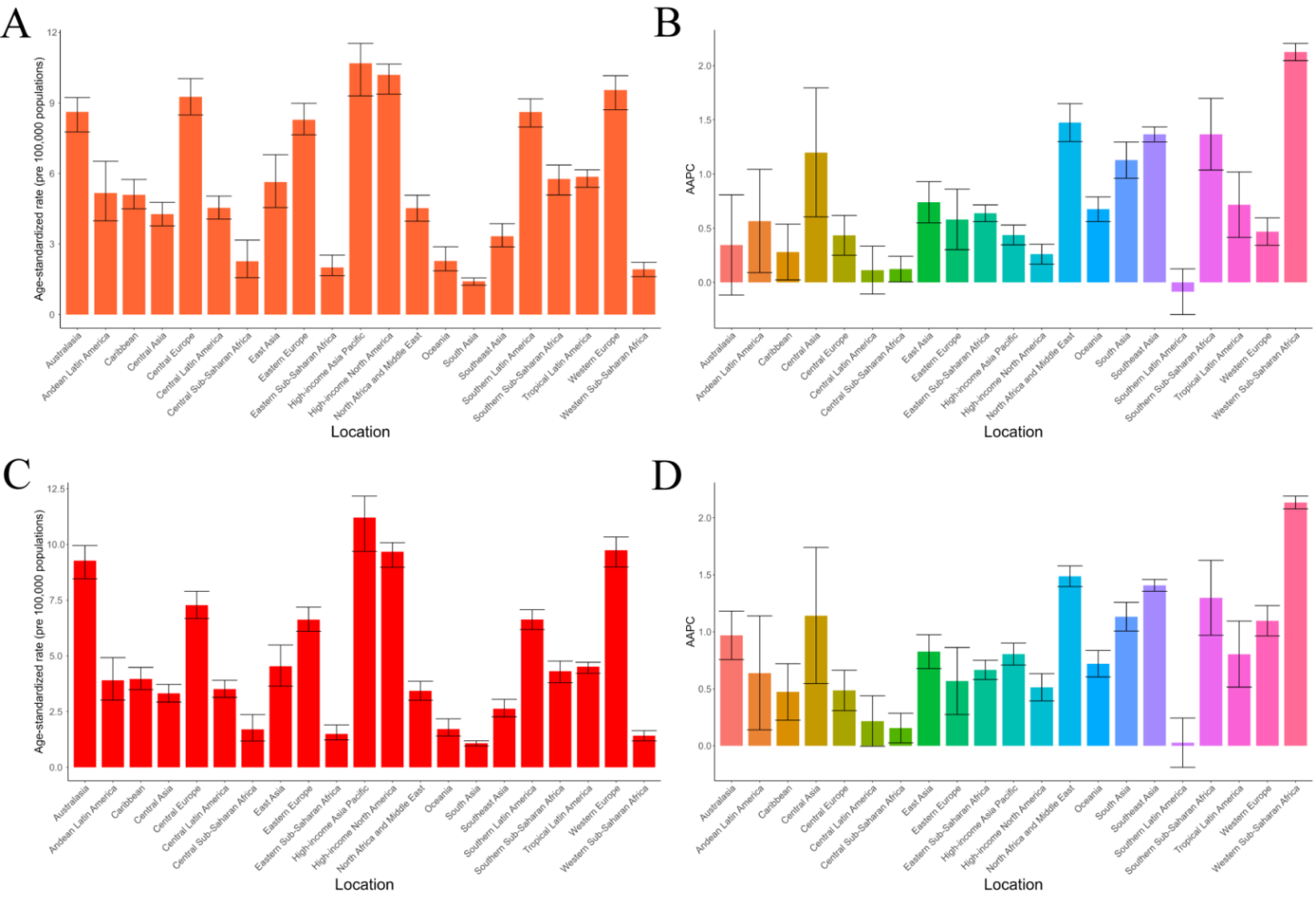


# Figure S2. The ASRs burden of pancreatic cancer incidence (A) and prevalence (C) in 2021 across the 21 GBD regions globally and their AAPCs (B, D) from 1990 to 2021.

*Abbreviations: ASR, age-standardized rate; GBD, Global Burden of Disease; AAPC, average annual percentage change.*


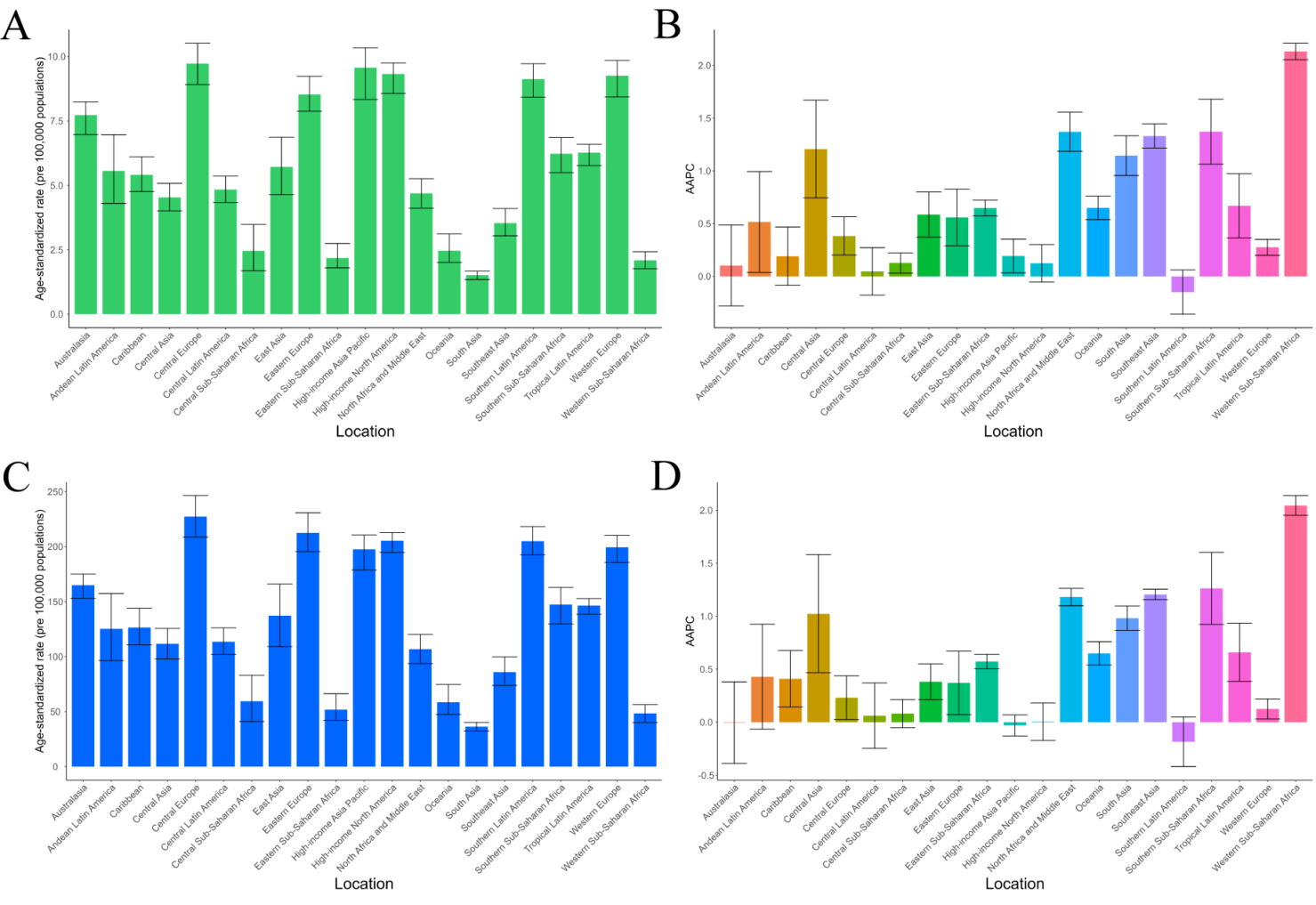


# Figure S3. The ASRs burden of pancreatic cancer mortality (A) and DALYs (C) in 2021 across the 21 GBD regions globally and their AAPCs (B, D) from 1990 to 2021.

*Abbreviations: ASR, age-standardized rate; GBD, Global Burden of Disease; AAPC, average annual percentage change; DALYs, disability-adjusted life years.*


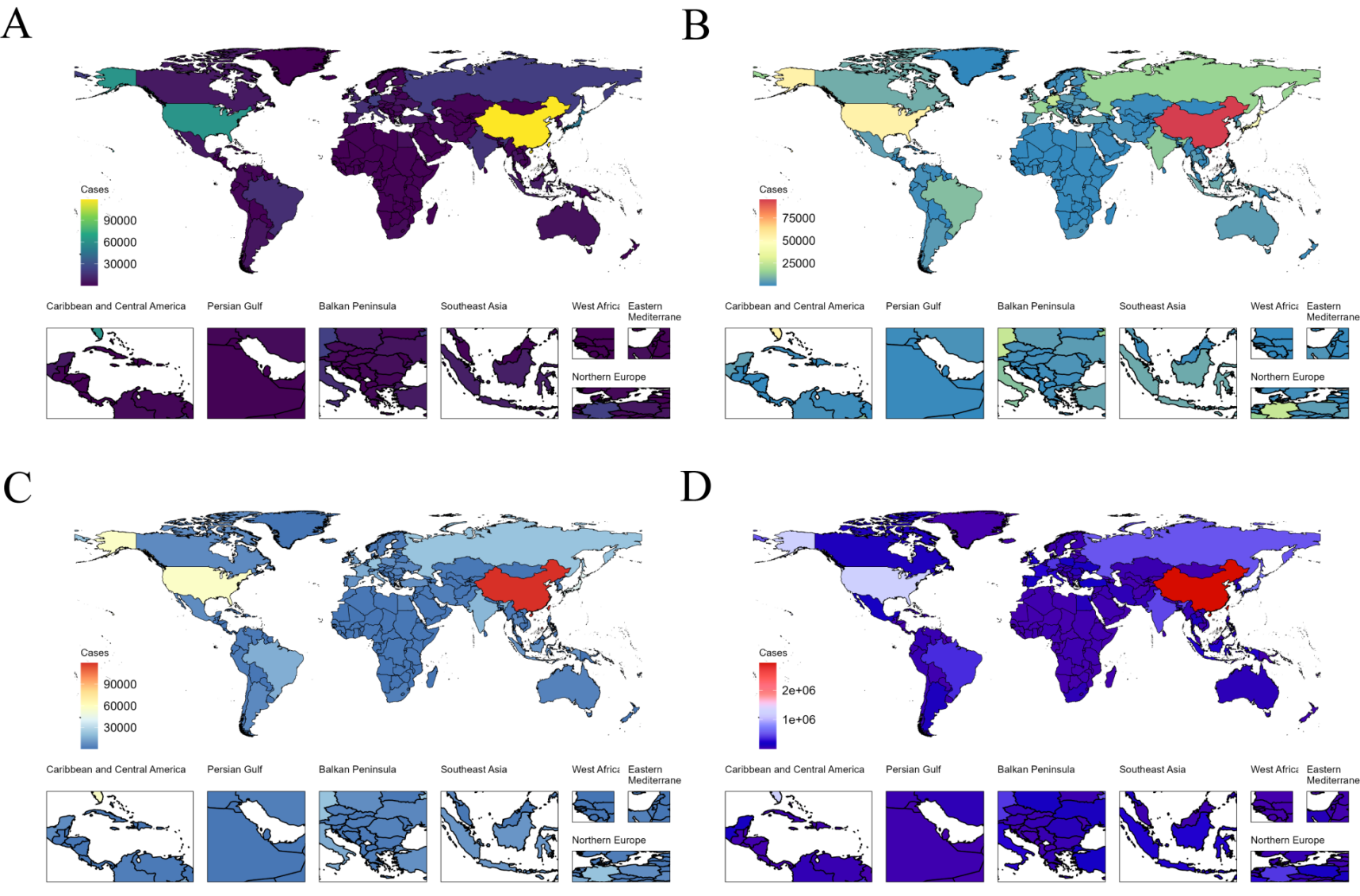


# Figure S4. The numbers of incidence (A), prevalence (B), mortality (C), and DALYs (D) for pancreatic cancer worldwide in 2021.

*Abbreviations: DALYs, disability-adjusted life years.*


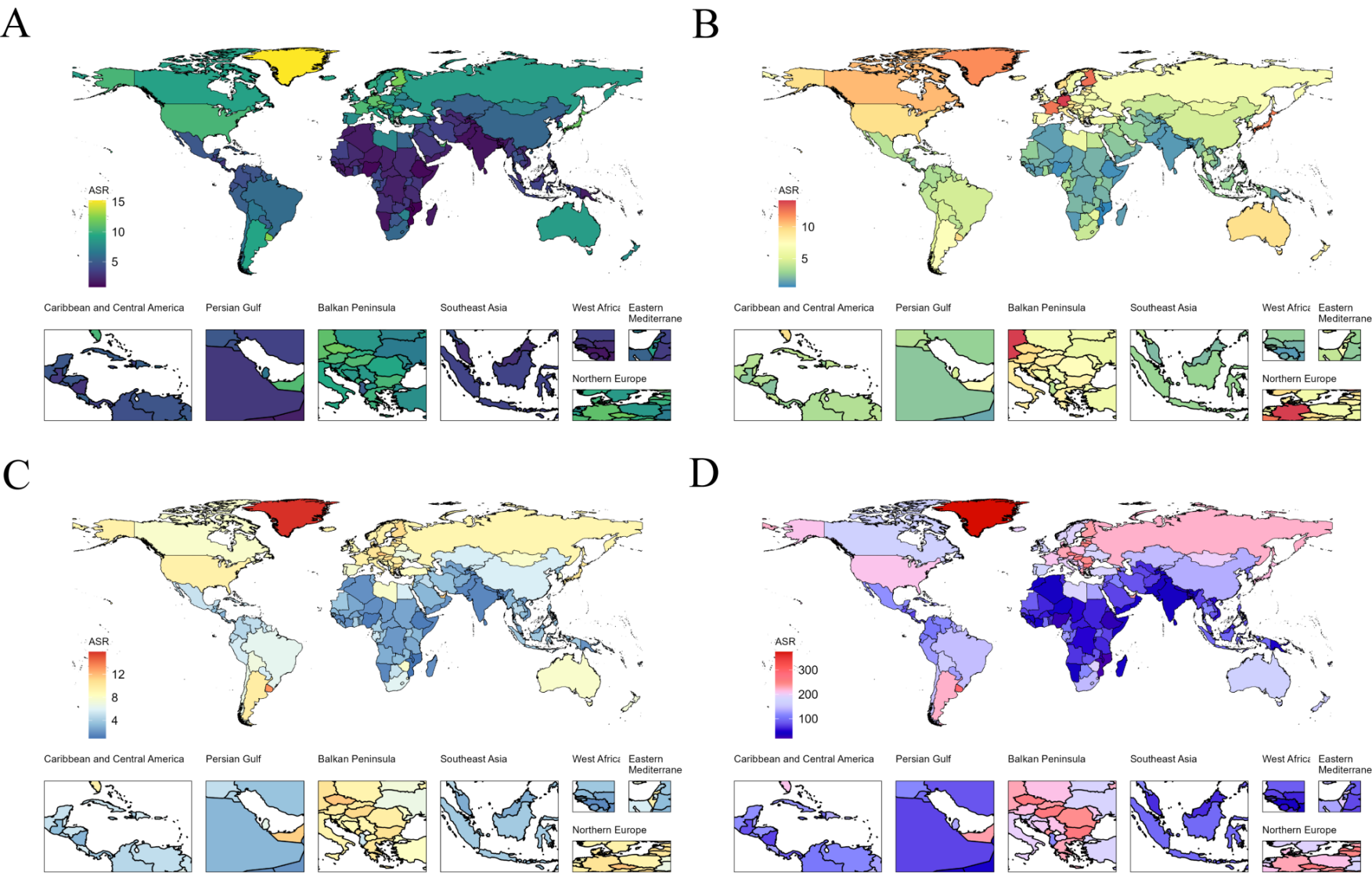


# Figure S5. The ASRs of incidence (A), prevalence (B), mortality (C), and DALYs (D) for pancreatic cancer worldwide in 2021.

*Abbreviations: ASR, age-standardized rate; DALYs, disability-adjusted life years.*


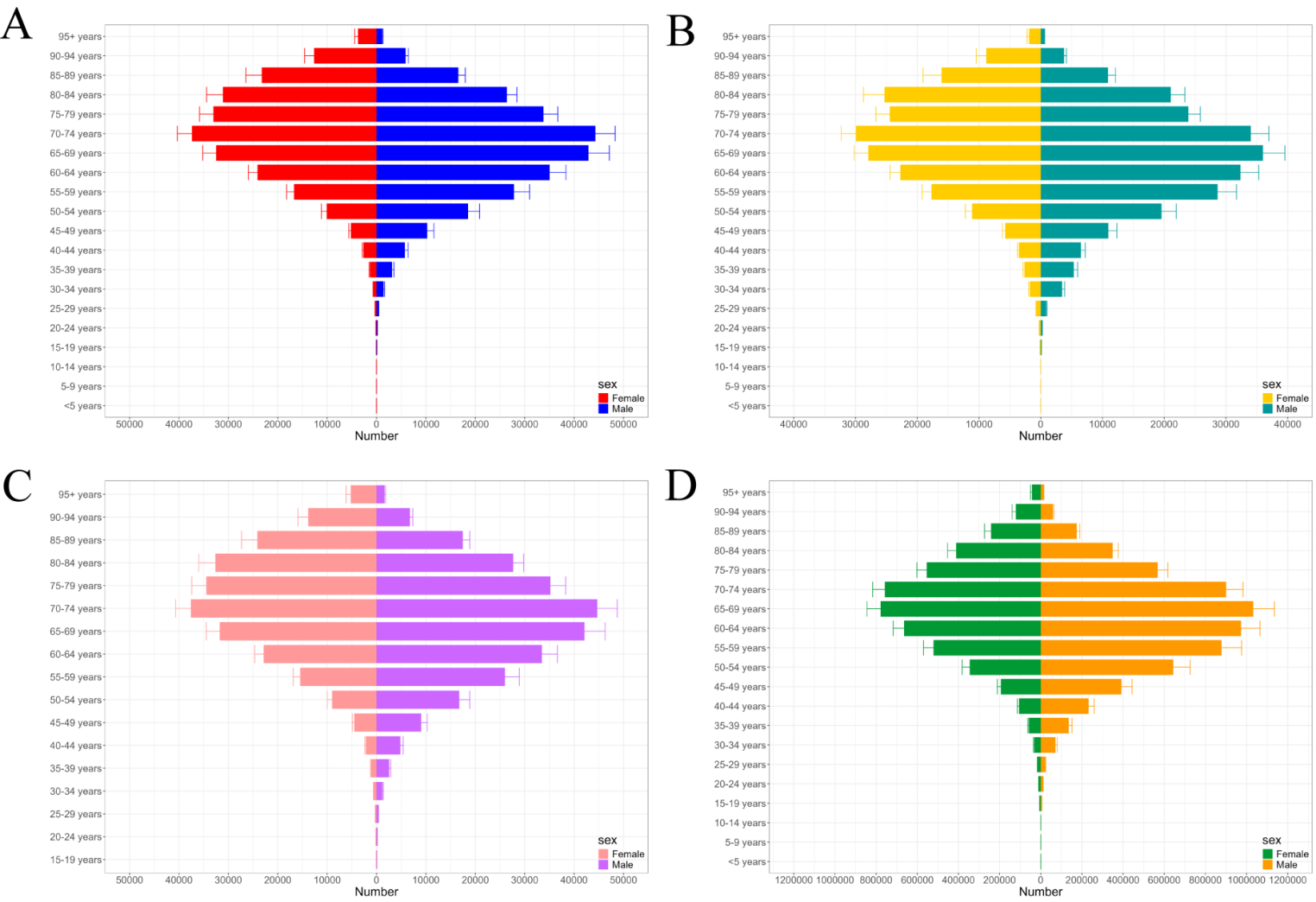


# **Figure S6.** The trends in numbers of incidence (A), prevalence (B), mortality (C), and DALYs (D) for pancreatic cancer across different genders by age groups ranging from under 5 years to 95+ years.

*Abbreviations: DALYs, disability-adjusted life years.*
